# Comparative effectiveness of statins and exercise in high-risk individuals: systematic review and network meta-analysis

**DOI:** 10.3389/fcvm.2025.1617799

**Published:** 2025-07-11

**Authors:** Jia-Li Chen, Jin-Tao Zhang, Xue-Hui Li, Ruo-Shan Wu, Hong-Tao Ma

**Affiliations:** ^1^ School of Education, Beijing Sport University, Beijing, China; ^2^ School of Physical Education, Hunan University of Science and Technology, Xiangtan, China

**Keywords:** arterial stiffness, high-risk populations for cardiovascular diseases, statins, exercise intervention, network meta-analysis, Whole-Body vibration training

## Abstract

**Background and aims:**

Arterial stiffness (AS) predicts cardiovascular disease (CVD) risk and relates to multiple factors. But the best interventions for AS in high—risk CVD groups are unknown. This review focuses on how different interventions affect AS and related indicators.

**Methods:**

We searched MEDLINE (PubMed), Embase, Cochrane Library, EBSCO, and Web of Science for relevant studies. Inclusion criteria: (1) randomized controlled trials (RCT); (2) participants with CVD risk factors as per American College of Sports Medicine (ACSM) guidelines; (3) interventions including Whole-Body Vibration (WBV), statins (STA), interval training (INT), aerobic exercise (AE), resistance exercise (RT), and combined exercise (CT); (4) control groups with usual care or placebo; (5) outcomes of pulse wave velocity (PWV), systolic blood pressure (SBP), and diastolic blood pressure (DBP); (6) studies in English. Data were analyzed using a random effects network meta-analysis and assessed for bias using the Cochrane tool.

**Results:**

This meta-analysis of 58 studies (*n* = 2,931) found all long-term interventions (STA, WBV, CT, RT, AE, INT) significantly reduced PWV (*p* < 0.001). WBV, INT, and AE notably lowered both SBP and DBP (*p* < 0.001). hSTA showed optimal PWV reduction (SUCRA=92.0), while WBV showed highest efficacy for SBP (SUCRA=94.0) and DBP (SUCRA=77.3).

**Conclusions:**

For CVD high-risk populations, high doses of statins (hSTA) optimally reduces AS; WBV is the top non-drug AS intervention, while INT best improves both AS and BP short-term. Combined, these interventions significantly enhance outcomes.

**Systematic Review Registration:**

https://www.crd.york.ac.uk/PROSPERO/display_record.php?RecordID=564538, PROSPERO CRD42024564538.

## Graphical Abstract

**Figure:**
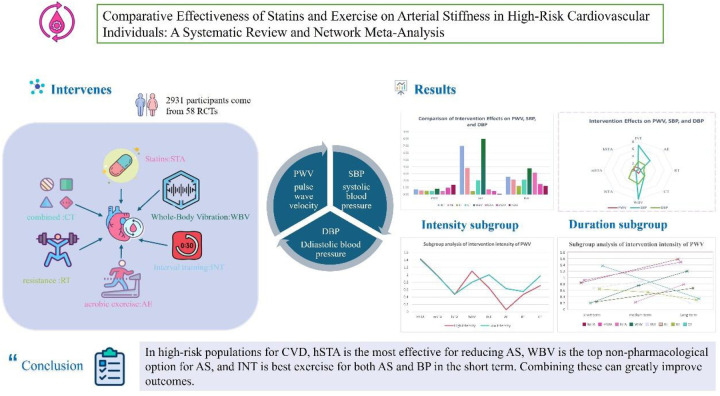

## 1 Introduction

### 1.1 Background & significance

Arterial stiffness (AS), measured by pulse-wave velocity (PWV), is an independent predictor of cardiovascular disease (CVD). A 1 m/s PWV increase elevates cardiovascular events by 12%–14% and CVD mortality by 13%–15% (1). AS promotes atherosclerosis through endothelial dysfunction and inflammation (2). Despite statins’ established role in AS management, preventive strategies like exercise remain critical due to challenges in early intervention (3).

### 1.2 Exercise controversies

Exercise can improve AS by enhancing arterial remodeling, boosting endothelial function, lowering sympathetic nervous system activity, and reducing inflammatory cytokines (4). However, optimal modalities for distinct populations remain controversial due to inconsistent study protocols and populations. Specifically for high-risk CVD patients, meta-analyses conflict on whether aerobic (AE) or interval training (INT) is superior for AS reduction [Ramos 2015 favoring INT (6) vs. Saz-Lara 2021 finding no difference (5)]. Resistance training (RT) effects are particularly contentious: high-intensity RT increased AS in young adults (8) but improved it in another study despite acute BP elevation (7), while moderate-intensity RT showed no effect in young adults and no association in middle-aged populations (8). Whole-Body Vibration (WBV) enhances endothelial function via mechanical stimulation (12–15), but its comparative efficacy is unknown. These inconsistencies necessitate population- and intensity-specific exercise prescriptions.

### 1.3 Aerobic exercise mechanisms

Aerobic exercise (AE) is often recommended for improving vascular function due to its acute impact on blood vessel dilators like calcium (Ca^2^^+^), potassium (K^+^), hydrogen ions (H^+^), and carbon dioxide (CO₂) (5). Acutely, AE induces endothelial shear stress, triggering nitric oxide (NO) release and transient arterial dilation within 30–90 min post-exercise (20). Long-term AE (>8 weeks) reduces AS through structural adaptations: decreased collagen deposition, increased elastin content, and attenuated vascular inflammation (21). A analysis confirmed sustained AS improvement after 3-month AE programs in CVD patients (22).

### 1.4 Statins & research Gap

Statins are a cornerstone therapy for arterial stiffness (AS), a major CVD risk factor (16). Beyond cholesterol reduction, they improve endothelial function and arterial compliance via anti-inflammatory/antioxidant mechanisms, directly reducing AS (17, 18), potentially independent of lipid-lowering (19), underscoring their role in CVD risk management (16). However, critical gaps remain in optimizing statin therapy within broader strategies for high-risk CVD patients. Although systematic reviews exist for isolated interventions [e.g., exercise (4), statins (16)], key limitations persist: (1) Existing meta-analyses [e.g., (5, 6)] focus on single modalities, lacking direct comparisons between pharmacological (e.g., statins) and non-pharmacological (e.g., exercise) strategies; (2) Crucially, no review quantifies dose-response relationships for statin intensity (L/m/hSTA) vs. exercise types. This gap is clinically significant, resulting in absent integrated guidelines for combining statins, WBV, and multi-modal exercise to manage AS in high-risk individuals.

### 1.5 Study aim

The study aims to bridge this gap by systematically evaluating and comparing the effects of: Interval Training (INT), Aerobic Exercise (AE), Resistance Training (RT), Combined Training (CT), low- (lSTA), moderate- (mSTA), and high-intensity statins (hSTA), and Whole-Body Vibration (WBV) on arterial stiffness (primary outcome) and blood pressure (SBP/DBP, secondary outcomes) in adults at high risk for cardiovascular disease. The findings will provide evidence-based guidance for tailoring effective AS management strategies in this vulnerable population.

## 2 Methods

### 2.1 Registration

The study protocol was registered with PROSPERO (CRD42024564538) (23). The systematic review and network meta-analysis (NMA) adhered to the PRISMA-NMA guidelines.

### 2.2 Literature search strategy

We systematically searched MEDLINE (PubMed), Embase, the Cochrane Library, EBSCO, and Web of Science databases using a detailed electronic search strategy, outlined in Supplementary Appendix 2. The search strategy was based on key phrases related to the PICOS tool: (P) Population: “Hypertension” or “obesity” or “Type 2 diabetes” or “T2D” or “metabolic syndrome” or “older persons over 60 years”; (I) Intervention: “statins” or “simvastatin” or “rosuvastatin” or “lovastatin” or “fluvastatin” or “atorvastatin” or “WBV” or “whole-body vibration training” or “physical activity” or “training” or “aerobic exercise” or “moderate intensity continuous training” or “moderate interval training” or “high interval training” or “resistance training” or “strength training” or “combined training” or “sprint interval training” or “high intensity interval training”; (C) Comparator: “control group” or “no exercise” or “usual care” or “placebo-control”; (O) Outcomes: “arterial stiffness” or “pulse wave velocity” or “PWV”; and (S) Study type: “randomized controlled trial” or “randomized” or “placebo” or “RCT”. The search was restricted to English-language articles published from the inception of the databases through June 2024. We included RCTs that compared different exercise types on AS in high-risk populations for CVD.

### 2.3 Study selection

Duplicates were first removed using EndNote X9 software (Clarivate Analytics, Philadelphia, PA). Two researchers (JLC and HTM) then independently screened titles and abstracts to identify potentially relevant studies. These reviewers also independently assessed the studies for inclusion criteria. Discrepancies were resolved through discussion or, if needed, consultation with a third expert (JTZ). The inclusion criteria were: (1) Studies must be RCTs; (2) Subjects must have a known risk factor for CVD; (3) Interventions must include AE, RT, INT, CT, WBV, and STA; (4) Comparators must include no intervention, usual care, and placebo-control; (5) Outcomes must include PWV, SBP, and DBP; (6) Studies must be published in English.

Exclusion criteria were: (1) Healthy adult subjects; (2) Animal studies or randomized crossover trials; (3) Acute exercise interventions (<3weeks); (4) Incomplete data or lack of a control group; (5) Reviews, duplicate publications, letters to the editor, and meta-analyses.

### 2.4 Intervene categories

In the included RCTs, interventions comprised of INT, AE, RT, CT, WBV, and STA. As depicted in Table 1, we operationally defined these intervention as follows:

**Table 1:** Definition of the types of exercise.

Type	Definition
INT	Frequency: 2–3 times per week
	Intensity: >65% VO₂max, >65% HRR, or >75% HRmax
	Duration per Session: 20–30 min
	Mode: Any intermittent traditional interval training, including MIIT and HIIT (e.g., walking, running, cycling, rowing, swimming, elliptical, and stepping exercises) (24)
HIIT	Frequency: 3–5 times per week
	Intensity: 75%–90% HRmax, 65%–85%VO₂max, or 65%–85% HRR
	Duration per Session: ≥60 min
	Interval Time: <40 s
MIIT	Frequency: 3–4 times per week
	Intensity: 75%–90% HRmax, 65%–85% VO2max^a^, or 65%–85% HRR
	Duration per Session: 30–59 min
	Interval Time: 40–90 s
AE	Frequency: 3–5 times per week
	Intensity: >45% VO₂max, >50% HRR, or >65% HRmax
	Duration per Session: 30–60 min
	Mode: Any continuous aerobic exercise (e.g., walking, running, cycling, rowing, swimming, aerobics, elliptical, stepping)
RT	Frequency: 2–3 times per week
	Intensity: ≥50% 1RM
	Duration per Session: 30–60 min
	Mode: Any resistance training, including circuit-based programs (e.g., free weights, weight machines, resistance bands) (14)
CT	A combination of AE and RT
WBV	included six leg exercises standing on a WBV platform
lSTA	STA dosage <10 mg once daily
mSTA	STA dosage of 10–20 mg once daily
hSTA	STA dosage >10 mg once daily
CON	No exercise or usual care or placebo

VO_2_max, maximal oxygen uptake; HRR, heart rate reserve; HRmax, maximum heart rate; RM, repetition maximu.

### 2.5 Outcomes

The primary outcome of this study was PWV, with secondary outcomes including SBP and DBP. The cfPWV (carotid-femoral pulse wave velocity) is the gold standard for AS, defined as the distance between measurement sites divided by the time for the pulse wave to travel. A PWV >10 m/s indicates AS (25). This study mainly used cfPWV, with some baPWV (brachial-ankle pulse wave velocity) measurements. baPWV correlates with cfPWV but has limitations due to artery elasticity and measurement interference (26). SBP is the maximum pressure during heart contraction, DBP is the pressure when the heart is relaxed, and PP is the difference between SBP and DBP (27). Stage I hypertension is SBP 130–139 mmHg and/or DBP 80–89 mmHg.

### 2.6 Data extraction

Data extraction was conducted independently by two investigators (JLC and HTM). Any disagreements were resolved through consensus or by consulting a third author (JTZ) if necessary. Collected information included the first author, publication year, country, subject characteristics (sample size, gender, age, PWV, SBP, DBP, and concomitant diseases), intervention details (type, intensity, duration, frequency, and supervision status), and outcome measures reported in each eligible study, as outlined in Supplementary Appendix 4. For studies with insufficient information, email communication was used to obtain the missing values.

### 2.7 Risk of bias and GRADE assessment

The risk of bias (ROB) in the included studies was assessed by two researchers using the Cochrane Risk of Bias tool (28), which covers seven domains: (a) allocation generation, (b) allocation concealment, (c) blinding of participants and personnel, (d) blinding of outcome assessment, (e) handling of incomplete outcome data, (f) freedom from selective reporting bias, and (g) other forms of bias. Due to the difficulty in blinding participants to exercise interventions, this component was not factored into the overall ROB score. Studies were categorized into three risk levels: low risk if none of the domains were rated as high risk and ≤3 domains were rated as unclear; moderate risk if one domain was rated as high risk or no domain was high risk but ≥4 domains were unclear; high risk for all other cases (29). The Grading of Recommendations Assessment, Development and Evaluation (GRADE) framework was used to assess the certainty of the evidence for both primary and secondary outcomes (30).

### 2.8 Data synthesis and statistical analyses

The experimental effect was estimated by combining the pre-to-post changes of both the experimental and CON. The standard deviation (SD) of the change value was calculated using the formula provided in the Cochrane Handbook (version 6.3) (31). (the formula is $SD_{\mathrm{change}}=\sqrt{{\;\mathrm{SD}}_{\mathrm{baseline}}^{2}+{\mathrm{SD}}_{\mathrm{final}}^{2}D\text{-}(2\times \mathrm{Corr}\times SD_{\mathrm{baseline}}\;\times SD_{\mathrm{final}})}$

Heterogeneity among studies was evaluated using network estimates and pairwise meta-analytic techniques with Review Manager 5.3 (Nordic Cochrane, Denmark). Sensitivity analysis was performed to examine this heterogeneity (32). Pooled effect estimates were computed with a random-effects model, and mean differences (MD) were assessed for PWV, SBP, and DBP. Heterogeneity was measured using the I^2^ statistic and Cochran's *Q* test, with significant heterogeneity defined as I^2^ > 50% or *p* ≤ 0.10 (33). Publication bias was assessed using a funnel plot and Begg's test.

Statistical analysis was conducted using STATA 16.0 (STATA Corp, College Station, TX, USA) within a frequentist framework and random-effects multivariate network meta-analysis (NMA) (34). Weighted mean differences were reported for continuous variables, with 95% confidence intervals (CI) and 95% prediction intervals. Interventions were compared using network geometry, where node size and line thickness indicated the number of studies and the direct relationships between interventions.

Inconsistencies were evaluated using loop-specific, node-splitting, and global methods to assess ring, local, and global inconsistencies (35). A *p*-value < 0.05 indicated inconsistency, allowing further NMA analysis (36). The transitivity assumption was checked using a consistency model to ensure valid comparisons and random allocation (36, 37). The network contribution diagram showed the impact of each direct comparison on the NMA results.

The cumulative ranking curve (SUCRA) was used to rank different exercise modalities (31). SUCRA values range from 0 to 100, with higher values representing better outcomes (38, 39,40). Publication bias was also evaluated using a funnel plot and symmetry criterion.

## 3 Results

### 3.1 Literature selection

**Figure 1:**
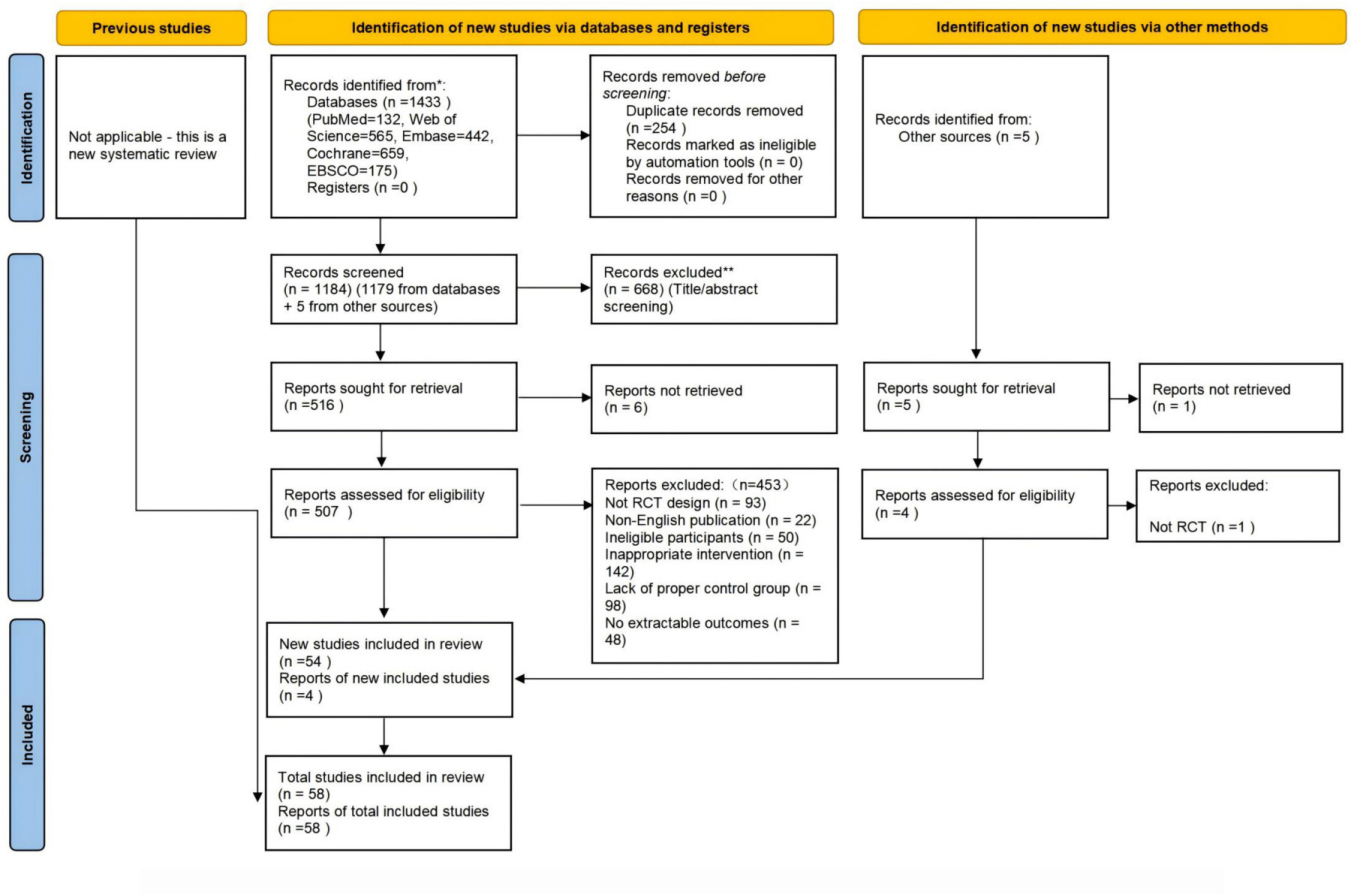
Preferred reporting items for systematic reviews and meta-analyses (PRISMA 2020) flow diagram of each stage of the study selection. Note. RCT indicates randomized controlled trial.

We retrieved 1,973 articles from the database and an additional 5 from other sources, totaling 1,978 articles. After removing 799 duplicates, 1,179 unique articles remained. We then reviewed the titles and abstracts, excluding 668 articles and leaving 511 candidates. A further review of the full texts led to the exclusion of 453 more articles, resulting in 58 articles that met our criteria. Thus, 58 eligible articles were identified, as shown in Figure 1.

### 3.2 Characteristics of the included studies

Supplementary Appendix 4 outlines the characteristics of the studies included in our review, conducted between 2003 and 2024 across various regions: Asia (18 studies), North America (19), South America (2), Europe (13), Oceania (2), Latin America (2), and Africa (2). In total, these studies involved 2,931 subjects at high risk for CVD. The experimental group consisted of 1,538 subjects, further categorized into subgroups: INT: 369, AE: 351, RT: 153, CT: 209, WBV: 58, lSTA: 48, mSTA: 251, and hSTA: 99. The control group included 1,393 subjects.

Approximately 50.3% of participants were female, with ages ranging from 10 to 75 years. The studies differed in gender focus: 10 included only males, 11 only females, and 37 included both genders. All 58 studies targeted individuals at high risk for CVD, adhering to American College of Sports Medicine (ACSM) guidelines, which recognize obesity, diabetes, hypertension, metabolic disorders, and advanced age as significant risk factors.

Types of statin therapy include simvastatin, rosuvastatin, lovastatin, fluvastatin, and atorvastatin. In the study, statin dosages were categorized as lSTA (less than 10 mg once daily), mSTA (10–20 mg once daily), and hSTA (more than 20 mg once daily). Exercise interventions included INT, AE, RT, CT (AE + RT), and WBV, with intensity ranging from high to low. The duration of these interventions varied from 8 to 96 weeks and encompassed activities such as running, cycling, rowing, swimming, stepping exercises, free weights, weight machines, and resistance bands. Exercise frequency ranged from 3 to 7 sessions per week, with each session lasting between 30 and 90 min. The control group received usual care, placebo, active control, or no exercise. Among the studies, 38 involved supervised exercise interventions, 3 combined supervisions with home-based interventions, and 17 had no supervision.

Carotid-femoral pulse wave velocity (cfPWV) was measured using applanation tonometry with the SphygmoCor CPV device (AtCor Medical, Australia) or SphygmoCor software version 9.0 (AtCor Medical Pty. Ltd., West Ryde, Australia). CfPWV was calculated by recording pressure pulse waves at the carotid and femoral arteries with a high-fidelity micromanometer (Millar Instruments, Houston, TX) and dividing the distance between the recording sites by the time delay between the carotid and femoral pulse waves (37, 46).

### 3.3 Results of ROB assessment

The risk of bias (ROB) assessments for each study are detailed in Supplementary Appendix 5. Among the studies, two had a high risk of bias due to randomization procedures, while 18 studies employed appropriate allocation concealment methods. Blinding of outcome assessment was associated with a low risk of bias in 45 studies, whereas eight studies exhibited a high risk of bias related to missing outcome values. Selective reporting posed a low risk of bias in 14 studies. Additionally, studies with sample sizes smaller than 10 or significant measurement errors were classified as having a high risk of other biases, with 44 studies demonstrating a low risk of such biases. In summary, 37 articles were rated as having low ROB, while 15 and 6 articles were rated as having moderate and high ROB, respectively.

### 3.4 Direct pairwise meta-analyses

#### 3.4.1 Primary outcome

**Figure 2:**
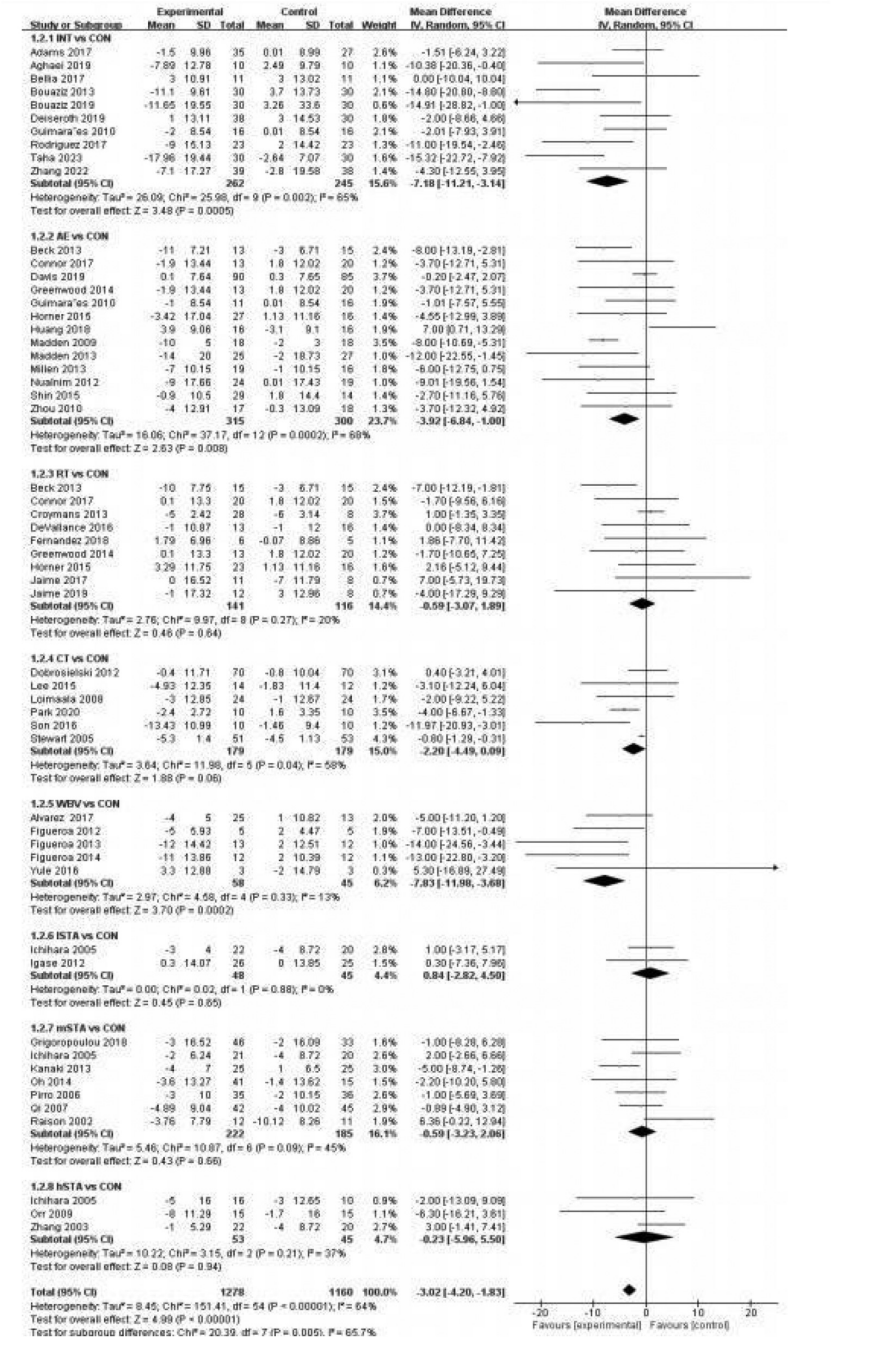
Forest plot of pulse wave velocity.

Figure 2 presents forest plots showing the differences and heterogeneity in the effects of exercise (INT, AE, RT, CT, and WBV) and statins (lSTA, mSTA, and hSTA) on improving PWV compared to the CON.

A pairwise meta-analysis was conducted, and the forest plot of PWV for exercise and statin categories is shown with detailed trial-level information (Figure 2). Compared to the CON, RT significantly decreased PWV with an SMD of −0.38 [*p* = 0.09, 95% CI (−0.59, −0.17), I^2^ = 40%], indicating lower heterogeneity. In contrast, INT [SMD = −0.69, *p* < 0.00001, 95% CI (−1.03, −0.35), I^2^ = 71%], AE [SMD = −0.61, *p* < 0.00001, 95% CI (−1.12, −0.10), I^2^ = 94%], CT [SMD = −0.69, *p* < 0.00001, 95% CI (−1.60, 0.24), I^2^ = 98%], lSTA [SMD = −0.44, *p* = 0.05, 95% CI (−0.98, 0.09), I^2^ = 74%], and mSTA [SMD = −0.92, *p* = 0.005, 95% CI (−1.65, −0.19), I^2^ = 65%] also significantly reduced PWV but with higher heterogeneity. WBV and hSTA showed no significant effect on PWV compared to the CON (*p* > 0.1). Funnel plots and Begg's test identified publication bias in the RT and hSTA subgroups, while other subgroups showed no evidence of publication bias (Supplementary Appendix 6.1).

#### 3.4.2 Secondary outcomes

The forest plot of secondary outcomes, including SBP and DBP, for exercise and statin categories is shown in Supplementary Appendix 7.2–7.3. The pairwise meta-analysis results indicated that INT, AE, CT, and mSTA significantly reduced SBP, while INT, AE, RT, and lSTA significantly decreased DBP (*p* < 0.01). Funnel plot analysis and Begg's test identified publication bias for RT, WBV, lSTA, and mSTA in SBP, and for INT, RT, and mSTA in DBP (*p* < 0.001). No publication bias was observed in other subgroups (Supplementary Appendix 6.2, 6.3).

### 3.5 Network meta-analysis

This study primarily focused on PWV as the main outcome and blood pressure parameters, specifically SBP and DBP, as secondary outcomes, all of which were analyzed using NMA. Supporting materials included network evidence plots, loop-specific approaches, node-splitting techniques, global inconsistency assessments, network forest plots, network contribution plots, funnel plots, and cumulative ranking plots. Figure 2 presents an annotated graphical abstract.

**Figure 3:**
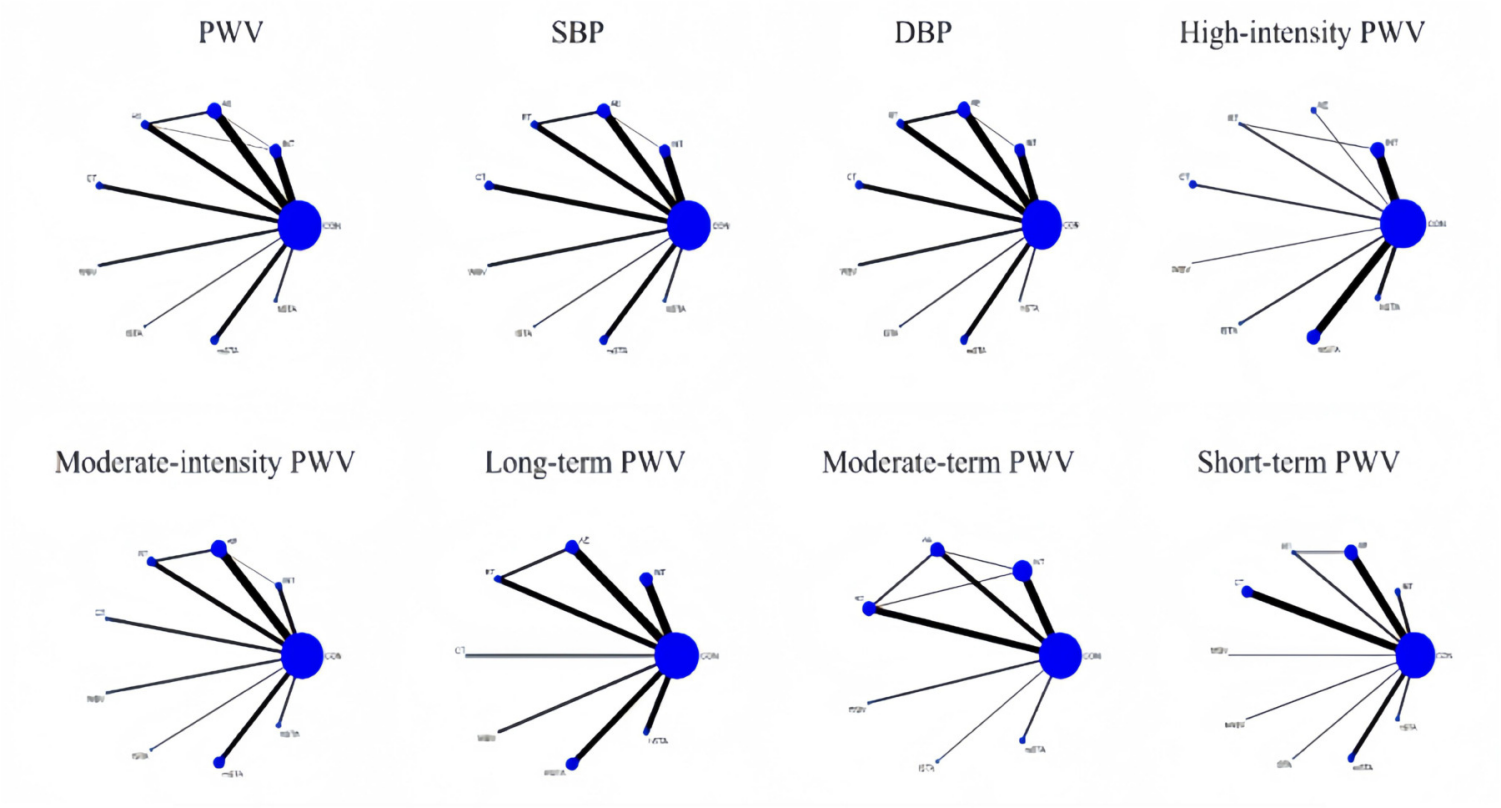
Network meta-analysis of eligible comparisons for PWV, SBP, and DBP, high-intensity PWV, moderate-intensity PWV, long-term PWV, moderate-term PWV, and short-term PWV.

The network evidence plot compares the differential impact of various exercise interventions on PWV and secondary outcomes. Figure 2 illustrates the NMA chart for PWV, SBP, and DBP. The connecting lines between nodes represent direct relationships between interventions, with the size of each node and the thickness of the lines proportional to the number of studies. As shown in Figure 3, AE intervention studies are predominant, whereas hSTA intervention studies are relatively scarce.

The inconsistency test plots include the loop-specific approach, node-splitting, and global inconsistency tests, which assess the consistency of PWV, SBP, and DBP at the loop, local, and global levels, respectively (Supplementary Appendix 9). The results of the loop-specific approach indicate that all closed loops involving PWV, SBP, and DBP exhibit good consistency, which showed inconsistency in PWV. Global inconsistency was assessed using an inconsistency model, and the results demonstrated that the *p*-values for PWV, SBP, and DBP were all greater than 0.05, indicating overall good consistency. Furthermore, the node-splitting analysis revealed no significant difference between indirect and direct comparisons (*p* > 0.05), suggesting reliable results (Supplementary Appendix 9).

The network forest plots illustrate the differences in intervention effects between various exercise and statins (including CON) through pairwise comparisons. Supplementary Appendix 10 displays the network forest diagrams for PWV, SBP, and DBP with 95% confidence intervals and 95% prediction intervals.

The contribution of direct and indirect comparisons to NMA is illustrated in the Network Contribution Graph (Supplementary Appendix 8), which also shows the number of studies for each direct comparison.

Funnel plots were used to assess publication bias in NMA, with PWV, SBP, and DBP all showing high symmetry, indicating an absence of publication bias.

Cumulative ranking curves (SUCRA) were employed to rank and compare the intervention effects of different types of exercise on PWV, SBP, and DBP. Supplementary Appendix 12 (1–4) presents the SUCRA probability rank results for various exercise and statins.

#### 3.5.1 Pooled estimates of primary outcomes

Table 2 presents the pooled estimates from the NMA of PWV. The interventions and their corresponding SMD, 95% CI, and *p*-values are as follows: hSTA [SMD = −1.39, 95% CI (−2.22, −0.56), *p* < 0.0001], mSTA [SMD = −0.96, 95% CI (−1.55, −0.36), *p* < 0.0001], WBV [SMD = −0.84, 95% CI (−1.51, −0.16), *p* < 0.0001], CT [SMD = −0.52, 95% CI (−1.02, −0.01), *p* < 0.0001], RT [SMD = −0.53, 95% CI (−0.96, −0.5), *p* < 0.0001], AE [SMD = −0.57, 95% CI (−0.93, −0.21), *p* < 0.0001], and INT [SMD = −0.77, 95% CI (−1.17, −0.36), *p* < 0.0001] all show significant improvements in PWV compared to CON. The SUCRA rankings in Table 3 reveal that hSTA (SUCRA = 92) is the most likely to be the best intervention for PWV, whereas lSTA (SUCRA = 38) is the least effective.

**Table 2:** The classifications for exercise according to the frequency, intensity, duration per session, and the length of intervention.

Classification type	Criteria
Frequency	
Low	1–2 times/week
Moderate	3–4 times/week
High	3 ≥ 5 times/week
Duration per session	
Short	<30 min
Moderate-1	30–44 min
Moderate-2	45–59 min
long	≥60 min
Length of intervention	
Short	<13 weeks (3 months)
Moderate long	14–25 weeks (3 months-6 months) ≥60 min (≥6 months)
Intensity	
Low	Interval training:
	< 75%HR max/ 65% V˙O2max/65% HRR
	Aerobic exercise: <60%HR max/ 60% ^V˙^O_2_max Resistance training: <50% of 1 RM
Moderate	Interval training:
	75%-90% HR max/65%–85% ^V˙^O2max/65%–85% HRR
	Aerobic exercise: 60%-85% HR max/60%–80% ^V˙^O_2_max Resistance training: 60%–80% of 1 RM
High	Interval training:
	>90% HR max/85% ^V˙^O2max/85% ^V˙^O2max
	Aerobic exercise: >85% HR max/80% ^V˙^O_2_max,
	Resistance training: 80%–100% of 1 RM

**Table 3:** Network meta-analysis matrix of PWV and secondary outcomes.

PWV in High intensity, m/s								
hSTA	0.43 (−0.59,1.45)	0.91 (−0.27,2.09)	0.55 (−0.52,1.62)	0.86 (−0.11,1.84)	0.86 (−0.08,1.79)	0.82 (−0.09,1.73)	0.62 (−0.30,1.55)	**1.39** (**0.56,2.22)**
−0.43 (−1.45,0.59)	**mSTA**	0.47 (−0.55,1.50)	0.12 (−0.78,1.02)	0.43 (−0.35,1.21)	0.43 (−0.31,1.16)	0.39 (−0.31,1.08)	0.19 (−0.53,0.91)	**0.96** (**0.36,1.55)**
−0.91 (−2.09,0.27)	−0.47 (−1.50,0.55)	**lSTA**	−0.36 (−1.43,0.72)	−0.04 (−1.01,0.93)	−0.05 (−0.99,0.89)	−0.09 (−0.99,0.82)	−0.28 (−1.21,0.64)	0.48 (−0.35,1.32)
−0.55 (−1.62,0.52)	−0.12 (−1.02,0.78)	0.36 (−0.72,1.43)	**WBV**	0.31 (−0.53,1.15)	0.31 (−0.49,1.11)	0.27 (−0.50,1.03)	0.07 (−0.71,0.86)	**0.84** (**0.16,1.51)**
−0.86 (−1.84,0.11)	−0.43 (−1.21,0.35)	0.04 (−0.93,1.01)	−0.31 (−1.15,0.53)	**CT**	−0.01 (−0.66,0.65)	−0.04 (−0.66,0.57)	−0.24 (−0.88,0.40)	**0.52** (**0.03,1.02)**
−0.86 (−1.79,0.08)	−0.43 (−1.16,0.31)	0.05 (−0.89,0.99)	−0.31 (−1.11,0.49)	0.01 (−0.65,0.66)	**RT**	−0.04 (−0.54,0.46)	−0.23 (−0.79,0.32)	**0.53** (**0.11,0.96)**
−0.82 (−1.73,0.09)	−0.39 (−1.08,0.31)	0.09 (−0.82,0.99)	−0.27 (−1.03,0.50)	0.04 (−0.57,0.66)	0.04 (−0.46,0.54)	**AE**	−0.20 (−0.71,0.32)	**0.57** (**0.21,0.93)**
−0.62 (−1.55,0.30)	−0.19 (−0.91,0.53)	0.28 (−0.64,1.21)	−0.07 (−0.86,0.71)	0.24 (−0.40,0.88)	0.23 (−0.32,0.79)	0.20 (−0.32,0.71)	**INT**	**0.77** (**0.36,1.17)**
**−1.39** (**−2.22,−0.56)**	**−0.96** (**−1.55,−0.36)**	−0.48 (−1.32,0.35)	**−0.84** (**−1.51,−0.16)**	**−0.52** (**−1.02,−0.03)**	**−0.53** (**−0.96,−0.11)**	**−0.57** (**−0.93,−0.21)**	**−0.77** (**−1.17,−0.36)**	**CON**
SBP, mm/Hg								
hSTA	−0.39 (−6.68,5.90)	0.87 (−7.08,8.81)	**−7.88** (**−15.18,−0.58)**	−1.92 (−8.14,4.31)	−0.35 (−6.65,5.95)	−3.71 (−9.72,2.31)	**−6.85** (**−12.95,−0.75)**	0.11 (−5.42,5.64)
0.39 (−5.90,6.68)	**mSTA**	1.26 (−5.18,7.69)	**−7.49** (**−13.13,−1.85)**	−1.53 (−5.68,2.62)	0.04 (−4.20,4.28)	−3.32 (−7.14,0.50)	**−6.46** (**−10.42,−2.51)**	0.50 (−2.47,3.48)
−0.87 (−8.81,7.08)	−1.26 (−7.69,5.18)	**lSTA**	**−8.74** (**−16.19,−1.30)**	−2.78 (−9.18,3.61)	−1.22 (−7.67,5.24)	−4.57 (−10.76,1.61)	**−7.72** (**−13.99,−1.45)**	−0.75 (−6.46,4.96)
**7.88** (**0.58,15.18)**	**7.49** (**1.85,13.13)**	**8.74** (**1.30,16.19)**	**WBV**	**5.96** (**0.38,11.54)**	**7.53** (**1.88,13.18)**	4.17 (−1.17,9.51)	1.03 (−4.41,6.47)	**7.99** (**3.21,12.77)**
1.92 (−4.31,8.14)	1.53 (−2.62,5.68)	2.78 (−3.61,9.18)	**−5.96** (**−11.54,−0.38)**	**CT**	1.57 (−2.61,5.74)	−1.79 (−5.53,1.95)	**−4.93** (**−8.82,−1.05)**	2.03 (−0.85,4.92)
0.35 (−5.95,6.65)	−0.04 (−4.28,4.20)	1.22 (−5.24,7.67)	**−7.53** (**−13.18,−1.88)**	−1.57 (−5.74,2.61)	**RT**	−3.36 (−6.85,0.14)	**−6.50** (**−10.47,−2.54)**	0.46 (−2.55,3.48)
3.71 (−2.31,9.72)	3.32 (−0.50,7.14)	4.57 (−1.61,10.76)	−4.17 (−9.51,1.17)	1.79 (−1.95,5.53)	3.36 (−0.14,6.85)	**AE**	−3.15 (−6.58,0.29)	**3.82** (**1.43,6.21)**
**6.85** (**0.75,12.95)**	**6.46** (**2.51,10.42)**	**7.72** (**1.45,13.99)**	−1.03 (−6.47,4.41)	**4.93** (**1.05,8.82)**	**6.50** (**2.54,10.47)**	3.15 (−0.29,6.58)	**INT**	**6.97** (**4.37,9.57)**
−0.11 (−5.64,5.42)	−0.50 (−3.48,2.47)	0.75 (−4.96,6.46)	**−7.99** (**−12.77,−3.21**)	−2.03 (−4.92,0.85)	−0.46 (−3.48,2.55)	**−3.82** (**−6.21,−1.43)**	**−6.97** (**−9.57,−4.37)**	**CON**
DBP, mm/Hg								
hSTA	−0.27 (−6.09,5.56)	−1.87 (−8.97,5.23)	−2.50 (−9.32,4.31)	−0.86 (−6.75,5.03)	2.52 (−3.26,8.30)	−0.89 (−6.43,4.65)	−1.30 (−6.86,4.27)	1.25 (−3.81,6.31)
0.27 (−5.56,6.09)	**mSTA**	−1.60 (−7.36,4.15)	−2.23 (−7.63,3.16)	−0.59 (−4.77,3.58)	2.79 (−1.23,6.80)	−0.63 (−4.29,3.03)	−1.03 (−4.72,2.67)	1.51 (−1.37,4.40)
1.87 (−5.23,8.97)	1.60 (−4.15,7.36)	**lSTA**	−0.63 (−7.39,6.13)	1.01 (−4.82,6.84)	4.39 (−1.33,10.10)	0.98 (−4.50,6.45)	0.57 (−4.92,6.07)	3.12 (−1.87,8.10)
2.50 (−4.31,9.32)	2.23 (−3.16,7.63)	0.63 (−6.13,7.39)	**WBV**	1.64 (−3.83,7.11)	5.02 (−0.33,10.37)	1.61 (−3.48,6.70)	1.21 (−3.91,6.32)	3.75 (−0.82,8.31)
0.86 (−5.03,6.75)	0.59 (−3.58,4.77)	−1.01 (−6.84,4.82)	−1.64 (−7.11,3.83)	**CT**	3.38 (−0.73,7.48)	−0.03 (−3.80,3.73)	−0.44 (−4.24,3.37)	2.11 (−0.91,5.13)
−2.52 (−8.30,3.26)	−2.79 (−6.80,1.23)	−4.39 (−10.10,1.33)	−5.02 (−10.37,0.33)	−3.38 (−7.48,0.73)	**RT**	**−3.41** (**−6.67,−0.15)**	**−3.81** (**−7.42,−0.20)**	−1.27 (−4.06,1.52)
0.89 (−4.65,6.43)	0.63 (−3.03,4.29)	−0.98 (−6.45,4.50)	−1.61 (−6.70,3.48)	0.03 (−3.73,3.80)	**3.41** (**0.15,6.67)**	**AE**	−0.40 (−3.54,2.74)	2.14 (−0.11,4.40)
1.30 (−4.27,6.86)	1.03 (−2.67,4.72)	−0.57 (−6.07,4.92)	−1.21 (−6.32,3.91)	0.44 (−3.37,4.24)	**3.81** (**0.20,7.42)**	0.40 (−2.74,3.54)	**INT**	**2.54** (**0.23,4.86)**
−1.25 (−6.31,3.81)	−1.51 (−4.40,1.37)	−3.12 (−8.10,1.87)	−3.75 (−8.31,0.82)	−2.11 (−5.13,0.91)	1.27 (−1.52,4.06)	−2.14 (−4.40,0.11)	**−2.54** (**−4.86,−0.23)**	**CON**

Effects are expressed as the effect size [95% CI] between interventions. Bold values indicate statistical significance (*p* < 0.05), light cyan areas indicate the effect of the longitudinal versus the lateral intervention, pink areas indicate the effect of the lateral versus the longitudinal intervention, grey areas represent the intervention category, and black areas represent the group. For example, “-0.77 (-1.17,-0.36)” (column 8, row 8), which indicates that INT (longitudinal intervention) significantly reduces PWV compared with CON (transverse intervention). CON, control group; INT, interval training; AE, aerobic exercise; RT, resistance training; CT, combined training; WBV, whole-body vibration training; lSTA, low doses of statins; mSTA, moderate doses of statins; hSTA, high doses of statins.

▪ Efficacy (response rate).

▪ Comparison.

▪ Acceptability (dropout rate).

▪ Group.

Effects are expressed as the effect size [95% CI] between interventions. Bold indicates that the data are significant, light cyan areas indicate the effect of the longitudinal vs. the lateral intervention, pink areas indicate the effect of the lateral vs. the longitudinal intervention, grey areas represent the intervention category, and black areas represent the group. For example, “−0.77 (−1.17, −0.36)” (column 8, row 8), which indicates that INT (longitudinal intervention) significantly reduces PWV compared with CON (transverse intervention).

CON, control group; INT, interval training; AE, aerobic exercise; RT, resistance training; CT, combined training; WBV, whole-body vibration training; lSTA, low doses of statins; mSTA, moderate doses of statins; hSTA, high doses of statins.

#### 3.5.2 Pooled estimates of the secondary outcome

The secondary indicators of this study were DBP and SBP. As shown in Table 3, WBV [SMD = −7.99, 95% CI (−12.77, −3.21), *p* < 0.0001], AE [SMD = −3.82, 95% CI (−6.21, −1.43), *p* < 0.0001], and INT [SMD = −6.97, 95% CI (−9.57, −4.37), *p* < 0.0001] significantly reduce SBP compared to CON. WBV [SMD = −2.54, 95% CI (−4.86, −0.21), *p* < 0.0001] significantly reduces DBP compared to CON. The SUCRA probability rankings in Table 3 indicate that WBV (SUCRA = 94) is the most likely to be the best intervention for SBP, whereas lSTA (SUCRA = 23.1) is the least effective. For DBP, WBV (SUCRA = 77.3) is the most likely to be the best intervention, while RT (SUCRA = 8.4) is the least effective.

#### 3.5.3 Subgroup NMA of primary outcome

We conducted subgroup analysis to assess the impact of intervention intensity and duration on PWV outcomes. Intervention intensity was categorized into high and medium-low groups, while intervention duration was classified into short, medium, and long cycles (Supplementary Appendix 15, 16).

Table 4 presents the results of subgroup analyses for intervention intensity and duration on PWV. The SUCRA probability rankings show that, at high intensity, WBV (SUCRA = 72.2) is the most effective sports intervention, excluding statins (lST, mSTA, hSTA) while CT (SUCRA = 23.1) is the least effective. At moderate intensity, CT (SUCRA = 69) is the most effective, and RT (SUCRA = 33.8) is the least effective. Regarding intervention duration, CT (SUCRA = 87.4) is the most effective in the short term, whereas AE (SUCRA = 29.6) is the least effective. In the medium term, INT (SUCRA = 90.5) is the most effective, while mSTA (SUCRA = 1.5) is the least effective. In the long term, hSTA (SUCRA = 83.3) is the most effective, and INT (SUCRA = 26.9) is the least effective.

**Table 4:** Subgroup analyses assessing potential moderating factors for PWV.

Group	Studies			PWV(m/s)			
	Rank		Reference	WMD (15% CI)	SUCRA	PrBest	MeanRank
Intervene intensity							
High intensity	1	hSTA	(49,54,55, 58)	−1.43 (−2.20, −0.66)	91.6	54.3	1.7
	2	mSTA	(47,48,50,52,53,56,57,58)	−0.98 (−1.53, −0.42)	76.3	8.4	2.9
	3	WBV	(44)	−1.10 (−2.85,0.65)	72.2	34.2	3.2
	4	INT	(4,5,6,8,10,11,12, 33)	−0.65 (−1.07, −0.23)	58.9	0.4	4.3
	5	lSTA	(51, 58)	−0.47 (−1.20,0.25)	49.2	1.2	5.1
	6	RT	(27,33,37,40,)	−0.47 (−1.12,0.18)	48.7	0.8	5.1
	7	AE	(22)	−0.06 (−1.06,0.94)	28.8	0.6	6.7
	8	CT	(42)	0.71 (−0.14,1.56)	3.2	0	8.7
Moderate intensity	1	hSTA	(49,54,55, 58)	−1.41 (−2.21, −0.61)	89.5	61.9	1.8
	2	INT	(1,2,3,8,9,10,13)	−1.00 (−1.64, −0.36)	69	12.9	3.5
	3	CT	(34,35,36,38,39,41)	−0.97 (−1.51, −0.42)	66.6	8.8	3.7
	4	mSTA	(47,48,50,52,53,56,57,58)	−0.97 (−1.54, −0.40)	66.5	9.2	3.7
	5	WBV	(42,43,45,46)	−0.80 (−1.48, −0.12)	52.8	5.1	4.8
	6	AE	(1,14,15,16,17,18,20,21, 23,24,25,28,29,30)	−0.63 (−0.99, −0.27)	38.8	0.2	5.9
	7	RT	(19,25,28,29,30,31,)	−0.55 (−1.04, −0.07)	33.8	0.3	6.3
	8	lSTA	(51, 58)	−0.48 (−1.25,0.30)	31.3	1.5	6.5
Intervene duration							
Short term	1	CT	(36)	−1.37 (−2.00, −0.74)	87.4	44.5	1.9
	2	mSTA	(50,56)	−0.92 (−1.75, −0.10)	66.2	10.6	3.4
	3	hSTA	(49,55)	−0.84 (−2.26,0.58)	60	18.2	3.8
	4	INT	(2,3,13)	−0.67 (−1.70,0.37)	52.7	6.4	4.3
	5	RT	(31,29)	−0.64 (−1.53,0.26)	52	3.8	4.4
	6	WBV	(46)	−0.20 (−2.84,2.44)	37.7	16.4	5.4
	7	AE	(14,21,29)	−0.24 (−0.92,0.44)	29.6	0.1	5.9
Medium term	1	INT	(1,4,8,9,10,11,12,33)	−0.89 (−1.25, −0.53)	90.5	56.4	1.6
	2	WBV	(43,45)	−0.75 (−1.32, −0.18)	77.6	31.1	2.3
	3	RT	(25,26,28,30,32,33)	−0.54 (−0.94, −0.13)	61	3.3	3.3
	4	AE	(1,17,24,25,30)	−0.51 (−1.04,0.03)	58.7	5.9	3.5
	5	lSTA	(51)	−0.23 (−0.93,0.47)	39.6	3.2	4.6
	6	mSTA	(47,53)	1.29 (−0.16,2.74)	1.5	0.1	6.9
Long term	1	hSTA	(54,58)	−1.58 (−2.66, −0.51)	83.3	30.3	2.5
	2	mSTA	(48,52,57,58)	−1.49 (−2.35, −0.63)	81.9	21.2	2.6
	3	lWBV	(42)	−1.20 (−2.99,0.59)	66.6	21.9	4
	4	hWBV	(44)	−1.10 (−3.05,0.85)	62.1	19.8	4.4
	5	lSTA	(58)	−0.79 (−2.18,0.60)	53	6.4	5.2
	6	AE	(15,16,19,20,22,23,24)	−0.67 (−1.21, −0.13)	50.3	0.1	5.5
	7	CT	(34,35,37,38,39,40,41)	−0.34 (−0.96,0.27)	32.8	0	7.1
	8	RT	(27,19)	−0.30 (−1.23,0.62)	30.8	0.2	7.2
	9	INT	(5,6,7)	−0.20 (−1.22,0.82)	26.9	0.2	7.6

Note: WMD, weighted mean difference; SUCRA, cumulative probability ranking; Intervention durations are classified as Short (< 1,000 min), Medium (1,000–2,000 minutes), and Long (> 2,000minutes). For vibration training, these correspond to 60, 180, and 360 min, respectively. Medication durations are < 12 weeks, 12–24 weeks, and > 24 weeks for short, medium, and long cycles, respectively. In the intensity subgroup, STA is the reference variable and is not ranked.

#### 3.5.4 GRADE assessment

Supplementary Appendix 14.3.1 presents the GRADE evaluation results for PWV, demonstrating high performance with most comparisons achieving low to moderate confidence. SBP and DBP were also assessed using the GRADE framework (see Supplementary Appendix 14). The results indicated low to moderate confidence for most SBP comparisons and low to moderate confidence for most DBP comparisons. Overall, PWV, SBP, and DBP were rated with moderate confidence in the GRADE assessment.

## 4 Discussion

### 4.1 Primary outcome

We assessed the impact of lSTA, mSTA, hSTA, WBV, INT, AE, RT, and CT on PWV in individuals at high risk for CVD. Our analysis found that all these interventions—hSTA, mSTA, WBV, INT, AE, RT, and CT—significantly reduced PWV and were clinically relevant (SMD > 0.5). SUCRA probability rankings identified hSTA as the most likely optimal intervention, WBV as the best non-pharmacological option, and INT as the most effective exercise intervention.

These findings align with previous research. In a subsequent meta-analysis, a different group concluded that statin therapy might be more effective than exercise for reducing AS (41). This supports our results showing a greater impact of statins compared to exercise. Variations in participant populations likely contribute to differences observed between studies comparing interventions. Limited research also suggests that WBV can significantly reduce AS (21).

However, our analysis suggests WBV may be a promising non-pharmacological intervention. While WBV appears to improve AS through enhanced systemic circulation and vascular function (42, 43), its lower intensity relative to traditional exercises warrants further investigation regarding cardiovascular benefits. Figure 2 indicates WBV effectively improves blood pressure, which may contribute to its impact on AS. These findings should be interpreted with caution given the limited number of WBV studies included in our meta-analysis.

Moreover, our study identifies INT as the most effective exercise intervention for improving AS. INT's alternating high and low intensity likely provides a highly efficient workout, maximizing cardiovascular adaptation through enhanced endothelial shear stress and nitric oxide bioavailability within shorter durations (44). In contrast, other forms of exercise may require longer durations to achieve similar results (6).

### 4.2 Secondary outcome

This study investigated the effects of various interventions on blood pressure (BP) in high-risk CVD populations. Our results demonstrated that WBV, AE, and INT significantly reduced SBP, while INT also lowered DBP. SUCRA rankings identified WBV as the optimal intervention for both SBP and DBP reduction (SUCRA = 94.0 and 77.3, respectively), whereas ISTA and RT were the least effective for SBP and DBP, respectively (Table 5).

**Table 5:** Ranking of exercise interventions in order of effectiveness.

PWV (65 studies, *N*=2,931)			
Treatment	SUCRA	PrBest (%)	Mean Rank
hSTA	92	69.7	1.6
mSTA	73.9	14.1	3.1
WBV	64.1	9.3	3.9
INT	60.9	2.4	4.1
AE	41.8	0.2	5.7
RT	39	0.5	5.9
CT	38.2	0.6	5.9
lSTA	38	3.2	6
CON	2	0	8.8
SBP (55 studies, *N*=2,438)			
Treatment	SUCRA	PrBest (%)	Mean Rank
WBV	94	63.4	1.5
INT	91.2	35.1	1.7
AE	70.7	0.7	3.3
CT	52.1	0.1	4.8
mSTA	32.6	0	6.4
RT	32.5	0	6.4
hSTA	29.9	0.4	6.6
CON	23.9	0	7.1
lSTA	23.1	0.2	7.2
DBP (54 studies, *N*=2,373)			
Treatment	SUCRA	PrBest (%)	Mean Rank
WBV	77.3	39.1	2.8
lSTA	68.4	28.2	3.5
INT	66.2	8.3	3.7
AE	59.6	4.5	4.2
CT	58.1	7.4	4.4
mSTA	47.7	3.1	5.2
hSTA	44.8	9.4	5.4
CON	19.6	0	7.4
RT	8.4	0	8.3

These findings align with prior evidence cited in the Introduction regarding the efficacy of AE and INT on BP. As noted, AE improves vascular function through modulation of vasodilatory agents (e.g., Ca^2^^+^, K^+^, H^+^, CO₂) (5), while INT's superiority over continuous AE may stem from its high-low intensity alternation, which enhances cardiovascular adaptation efficiency (6). Notably, WBV's leading role in BP management corroborates its proposed mechanism of action: mechanical vibrations stimulate muscle contractions, augmenting blood flow and endothelial function (12–14). This effect may be particularly advantageous for high-risk populations due to WBV's low-impact nature and time efficiency (14).

While WBV showed significant BP reduction, its long-term sustainability requires further validation, as highlighted in prior studies (45). Importantly, WBV's dual benefit on both AS (primary outcome) and BP supports the interplay between arterial stiffness and hypertension pathophysiology (4, 15). As emphasized in the Introduction, AS exacerbates BP elevation via endothelial dysfunction and inflammatory pathways (1, 2), suggesting that WBV's mechanical stimulation may concurrently ameliorate both processes.

### 4.3 Subgroup NMA of primary outcome

Subgroup analyses assessed how intervention intensity (high vs. moderate-low) and duration (short, medium, long) modulated PWV improvements (Table 4). Key findings included: Intensity: Higher intensity enhanced efficacy for WBV (SUCRA = 72.2 under high intensity) and RT, whereas AE performed better at moderate intensity (SUCRA = 38.8). Duration: Short-term effects were optimal for CT (SUCRA = 87.4), medium-term for INT (SUCRA = 90.5), and long-term for hSTA (SUCRA = 83.3). WBV and AE required longer durations for maximal benefit.

These intensity-dependent variations resonate with controversies outlined in the Introduction. For instance, while high-intensity RT may acutely increase BP (7), our data indicate it ultimately reduces PWV more effectively than lower intensities—consistent with its role in promoting arterial remodeling (9). Conversely, AE's superiority at moderate intensity aligns with evidence that vigorous AE may not further improve AS in obese populations (46), supporting tailored intensity prescriptions.

The duration-dependent efficacy underscores distinctions between rapid-acting (e.g., CT/INT in short/medium term) and sustained (e.g., hSTA/WBV in long term) interventions. INT's medium-term dominance (SUCRA = 90.5) reflects its efficiency in inducing rapid vascular adaptations (6), while WBV's reliance on longer exposure aligns with its proposed mechanism of gradual endothelial enhancement (12–14). This complements the Introduction's emphasis on CT's sensitivity to exercise sequence (10, 11): our results extend this by demonstrating CT's acute efficacy (short-term SUCRA = 87.4), likely due to synergistic AE-RT sequencing.

### 4.4 Strengths and limitations

This study used a network meta-analysis approach to simultaneously compare multiple intervention methods. This approach not only provides a broader perspective but also significantly enhances the value of the findings. We focused on High-risk populations for CVD, which have been relatively underexplored in past research. Since AS is a common complication of CVD, investigating this group is crucial for assessing the benefits of exercise in preventing and treating AS. We compared the effects of different statin intensities and various types of exercise on AS and BP in High-risk populations for CVD. Our analysis also included subgroup evaluations based on intervention intensity and duration, leading to significant findings. We recommend that High-risk populations for CVD prioritize WBV for improving AS and INT as the preferred exercise method. Additionally, moderate-intensity AE may be more effective over a longer intervention period.

However, this study has several limitations. First, we cannot confirm whether all possible intervention methods were considered. Future research should explore additional pharmacological treatments and exercise interventions. Second, while we focused on PWV, SBP, and DBP as key indicators of AS, other relevant measures such as ALX, blood lipids, and blood sugar were not included due to concerns about their validity. Addressing these limitations in future research with larger sample sizes and diverse interventions will be important. Challenges may include variability in study populations, differences in study designs, and unmeasured confounding factors.

## 5 Conclusion

This systematic review and network meta-analysis provide clear evidence on the most effective interventions for managing AS and BP in High-risk populations for CVD. hSTA is identified as the most effective treatment for reducing AS, while WBV is recognized as the leading non-pharmacological option for improving AS. INT emerges as the most effective exercise intervention for both reducing AS and lowering BP.

The analysis highlights that INT delivers significant cardiovascular benefits quickly due to its alternating high and low intensity, which maximizes cardiovascular adaptation. WBV and AE provide better results with longer durations, whereas CT, RT, and INT are more effective with shorter durations.

In managing BP, WBV, AE, and INT are all effective, with INT showing notable benefits in reducing DBP. Despite its lower intensity, WBV significantly impacts BP, underscoring its overall efficacy.

Future research should further explore the long-term effects of WBV and investigate additional pharmacological and exercise interventions. Standardizing study protocols and addressing variability among participants will enhance the accuracy and reliability of these findings.

In summary, hSTA and INT are highly effective for improving AS and cardiovascular health, while WBV and AE offer significant benefits for BP management. Choosing the appropriate type and duration of intervention is crucial for optimizing outcomes in High-risk populations for CVD.

## Data Availability

The datasets used and/or analyzed during the current study are available from the corresponding author on reasonable request.
