# Supplementary material for: Comparative effectiveness of statins and exercise in high-risk individuals: systematic review and network meta-analysis
Source: Front Cardiovasc Med. 2025 Jul 11;12:1617799. doi: 10.3389/fcvm.2025.1617799 (PMC12289655; doi:10.3389/fcvm.2025.1617799)
Supplement: Supplementary file 1 [file Datasheet1.pdf]

**Supplemental materials**	**Page**
**Appendix 1.** Completed PRISMA-NMA checklist.	1-7
**Appendix 2.** Search strategy of PubMed, Embase, Cochrane, Web of Science, and EBSCO.	8-12
**Appendix 3.** Transformation formulas and the classifications of exercise interventions.	13-16
**Appendix 4.** Characteristics of the included studies.	17-24
**Appendix 5.** Risk of bias assessment.	25-28
**Appendix 6.** Funnel plot of Pulse Wave Velocity, Systolic Blood Pressure, and Diastolic Blood Pressure in pairwise meta-analysis.	29-31
**Appendix 7.** Forest plots for Pulse Wave Velocity, Systolic Blood Pressure, and Diastolic Blood Pressure.	32-34
**Appendix 8.** Contributions of direct and indirect comparisons to NMA and the number of studies of each direct comparison of Pulse Wave Velocity, Systolic Blood Pressure, and Diastolic Blood Pressure.	35-37
**Appendix 9.** Inconsistency of Pulse Wave Velocity, Systolic Blood Pressure, and Diastolic Blood Pressure tested by loop-specific heterogeneity estimates, inconsistency model and node splitting analysis.	38-42
**Appendix 10.** Forest plots of eligible comparisons of Pulse Wave Velocity, Systolic Blood Pressure, and Diastolic Blood Pressure.	43-45
**Appendix 11.** The funnel plot graphics of Pulse Wave Velocity, Systolic Blood Pressure, and Diastolic Blood Pressure in NMA.	46-48
**Appendix 12.** Area under the curve for cumulative ranking probability of each intervention on Pulse Wave Velocity, Systolic Blood Pressure, and Diastolic Blood Pressure.	49-52
**Appendix 13.** Network of eligible comparisons for Pulse Wave Velocity, Systolic Blood Pressure, and Diastolic Blood Pressure.	53-55
**Appendix 14.** GRADE for Pulse Wave Velocity, Systolic Blood Pressure, and Diastolic Blood Pressure.	56-68
**Appendix 15.** Subgroup analysis of Pulse Wave Velocity in intervene intensity.	69-87
**Appendix 16.** Subgroup analysis of Pulse Wave Velocity in intervene duration.	88-113
**Appendix 17.** List of included studies.	114-122

Appendix 1. PRISMA NMA checklist of items to include when reporting a systematic review involving a network meta-analysis.

Section/Topic	Item #	Checklist Item	Reported on Page #
**TITLE**			
Title	1	Identify the report as a systematic review incorporating a network meta-analysis (or related form of meta-analysis).	1
**ABSTRACT**			
Structured summary	2	Provide a structured summary including, as applicable:<br>**Background:** main objectives<br>**Methods:** data sources; study eligibility criteria, participants, and interventions; study appraisal; and *synthesis methods, such as network meta-analysis.*<br>**Results:** number of studies and participants identified; summary estimates with corresponding confidence/credible intervals; treatment rankings may also be discussed. Authors may choose to summarize pairwise comparisons against a chosen treatment included in their analyses for brevity.<br>**Discussion/Conclusions:** limitations; conclusions and implications of findings.<br>**Other:** primary source of funding; systematic review registration number with registry name.	2
**INTRODUCTION**			
Rationale	3	Describe the rationale for the review in the context of what is already known, *including mention of why a network meta-analysis has been conducted.*_	4-5

Objectives	4	Provide an explicit statement of questions being addressed, with reference to participants, interventions, comparisons, outcomes, and study design (PICOS).	4-5
**METHODS**			
Protocol and registration	5	Indicate whether a review protocol exists and if and where it can be accessed (e.g., Web address); and, if available, provide registration information, including registration number.	6
Eligibility criteria	6	Specify study characteristics (e.g., PICOS, length of follow-up) and report characteristics (e.g., years considered, language, publication status) used as criteria for eligibility, giving rationale. Clearly describe eligible treatments included in the treatment network and note whether any have been clustered or merged into the same node (with justification)	6
Information sources	7	Describe all information sources (e.g., databases with dates of coverage, contact with study authors to identify additional studies) in the search and date last searched.	6
Search	8	Present full electronic search strategy for at least one database, including any limits used, such that it could be repeated.	6-7
Study selection	9	State the process for selecting studies (i.e., screening, eligibility, included in systematic review, and, if applicable, included in the meta-analysis).	6-7
Data collection process	10	Describe method of data extraction from reports (e.g., piloted forms, independently, in duplicate) and any processes for obtaining and confirming data from	6-7

		investigators.	
Data items	11	List and define all variables for which data were sought (e.g., PICOS, funding sources) and any assumptions and simplifications made.	8-9
**Geometry of the network**	**S1**	Describe methods used to explore the geometry of the treatment network under study and potential biases related to it. This should include how the evidence base has been graphically summarized for presentation, and what characteristics were compiled and used to describe the evidence base to readers.	9
Risk of bias within individual studies	12	Describe methods used for assessing risk of bias of individual studies (including specification of whether this was done at the study or outcome level), and how this information is to be used in any data synthesis.	9
Summary measures	13	State the principal summary measures (e.g., risk ratio, difference in means). Also describe the use of additional summary measures assessed, such as treatment rankings and surface under the cumulative ranking curve (SUCRA) values, as well as modified approaches used to present summary findings from meta-analyses.	9-11
Planned methods of analysis	14	Describe the methods of handling data and combining results of studies for each network meta-analysis. This should include, but not be limited to:<br>• Handling of multi-arm trials;<br>• Selection of variance structure;<br>• Selection of prior distributions in Bayesian analyses; and	9-11

		• Assessment of model fit.	
**Assessment of Inconsistency**	**S2**	Describe the statistical methods used to evaluate the agreement of direct and indirect evidence in the treatment network(s) studied. Describe efforts taken to address its presence when found.	10-11
Risk of bias across studies	15	Specify any assessment of risk of bias that may affect the cumulative evidence (e.g., publication bias, selective reporting within studies).	9
Additional analyses	16	Describe methods of additional analyses if done, indicating which were pre-specified. This may include, but not be limited to, the following: • Sensitivity or subgroup analyses; • Meta-regression analyses; • Alternative formulations of the treatment network; and • Use of alternative prior distributions for Bayesian analyses (if applicable).	9-10
**RESULTS†**			
Study selection	17	Give numbers of studies screened, assessed for eligibility, and included in the review, with reasons for exclusions at each stage, ideally with a flow diagram.	11
**Presentation of network structure**	**S3**	Provide a network graph of the included studies to enable visualization of the geometry of the treatment network.	Figure 1
**Summary of network geometry**	**S4**	Provide a brief overview of characteristics of the treatment network. This may include commentary on the abundance of trials and randomized patients for the different interventions and pairwise comparisons in the	12-13

		network, gaps of evidence in the treatment network, and potential biases reflected by the network structure.	
Study characteristics	18	For each study, present characteristics for which data were extracted (e.g., study size, PICOS, follow-up period) and provide the citations.	Appendix 17
Risk of bias within studies	19	Present data on risk of bias of each study and, if available, any outcome level assessment.	Appendix 5
Results of individual studies	20	For all outcomes considered (benefits or harms), present, for each study: 1) simple summary data for each intervention group, and 2) effect estimates and confidence intervals. Modified approaches may be needed to deal with information from larger networks.	13-14
Synthesis of results	21	Present results of each meta-analysis done, including confidence/credible intervals. In larger networks, authors may focus on comparisons versus a particular comparator (e.g., placebo or standard care), with full findings presented in an appendix. League tables and forest plots may be considered to summarize pairwise comparisons. If additional summary measures were explored (such as treatment rankings), these should also be presented.	14-17, Table 3
**Exploration for inconsistency**	**S5**	Describe results from investigations of inconsistency. This may include such information as measures of model fit to compare consistency and inconsistency models, *P* values from statistical tests, or summary of inconsistency estimates from different parts of the treatment network.	16, Appendix 9
Risk of bias	22	Present results of any assessment of risk of bias across	17

across studies		studies for the evidence base being studied.	
Results of additional analyses	23	Give results of additional analyses, if done (e.g., sensitivity or subgroup analyses, meta-regression analyses, alternative network geometries studied, alternative of prior distributions for Bayesian analyses, and so forth).	16-17 Appendix 15-16
**DISCUSSION**			
Summary of evidence	24	Summarize the main findings, including the strength of evidence for each main outcome; consider their relevance to key groups (e.g., healthcare providers, users, and policymakers).	24-26
Limitations	25	Discuss limitations at study and outcome level (e.g., risk of bias), and at review level (e.g., incomplete retrieval of identified research, reporting bias). Comment on the validity of the assumptions, such as transitivity and consistency. Comment on any concerns regarding network geometry (e.g., avoidance of certain comparisons).	26
Conclusions	26	Provide a general interpretation of the results in the context of other evidence, and implications for future research.	28
**FUNDING**			
Funding	27	Describe sources of funding for the systematic review and other support (e.g., supply of data); role of funders for the systematic review. This should also include information regarding whether funding has been received from manufacturers of treatments in the network and/or whether some of the authors are	Not Applicable

	content experts with professional conflicts of interest that could affect use of treatments in the network.	

PICOS = population, intervention, comparators, outcomes, study design.

* Text in italics indicate wording specific to reporting of network meta-analyses that has been added to guidance from the PRISMA statement.

† Authors may wish to plan for use of appendices to present all relevant information in full detail for items in this section.

Appendix 2. Search strategy of PubMed, Embase, Cochrane, Web of Science, and EBSCO.

Appendix 2.1 Search strategy in PubMed(N=68)

Step	Search strategy	Results
#1	((((((((((((((statins [Title/Abstract])) OR (simvastatin [Title/Abstract])) OR (rosuvastatin [Title/Abstract])) OR (lovastatin [Title/Abstract])) OR (fluvastatin [Title/Abstract])) OR (atorvastatin [Title/Abstract])) OR (whole-body vibration training [Title/Abstract])) OR (WBV [Title/Abstract])) OR (physical activity[Title/Abstract])) OR (training[Title/Abstract])) OR (aerobic exercise[Title/Abstract])) OR (aerobic training[Title/Abstract])) OR (moderate intensity continuous training[Title/Abstract])) OR (resistance training[Title/Abstract])) OR (strength training[Title/Abstract])) OR (combined training[Title/Abstract])) OR (sprint interval training[Title/Abstract])) OR (high intensity interval training[Title/Abstract])) OR ("Exercise"[Mesh])))))))))))))	820682
#2	(((arterial stiffness [Title/Abstract])) OR (PWV[Title/Abstract])) OR (pulse wave velocity [Title/Abstract])))	17685
#3	(((randomized controlled trial [Title/Abstract]) OR (randomized [Title/Abstract])) OR (placebo [Title/Abstract])) OR (RCT[Title/Abstract])))	795131
#4	((((control group [Title/Abstract]) OR (CON [Title/Abstract])) OR (usual care [Title/Abstract])) OR (placebo [Title/Abstract])) OR (active control [Title/Abstract]))))	3081609
#5	(((((Hypertension [Title/Abstract]) OR (Hypercholesterolemia [Title/Abstract])) OR (obesity [Title/Abstract])) OR (Type 2 diabetes [Title/Abstract])) OR (metabolic syndrome	928650

	[Title/Abstract])) OR (Older persons over 60 years [Title/Abstract])))))	
#6	#1 AND #2 AND #3 AND #4 AND #5	68

Appendix 2.2 Cochrane search results (*N*=441)

Step	Search strategy	Results
#1	MeSH descriptor: [Exercise] explode al trees	32970
#2	(statins): ti, ab, KW or (simvastatin): ti, ab, KW or (rosuvastatin): ti, ab, KW or (lovastatin): ti, ab, KW or (fluvastatin): ti, ab, KW or (atorvastatin): ti, ab, KW or (whole-body vibration training): ti, ab, KW or (WBV): ti, ab, KW or (training): ti, ab, KW or (aerobic exercise): ti, ab, KW or (aerobic training): ti, ab, KW or (resistance training): ti, ab, KW or (strength training): ti, ab, KW or (moderate intensity continuous Limits training): ti, ab, KW or (combined training): ti, ab, KW or (sprint interval training): ti, ab, KW or (high intensity interval training): ti, ab, KW or (physical activity): ti, ab, KW	160382
#3	#1 OR #2	171772
#4	(arterial stiffness) ti, ab, kw or (PWV):t, ab, kw or (pulse wave velocity) t, ab, kw	5988
#5	(randomized) ti, ab, kw or (randomized controlled trial) ti, ab, kw or (RCT): ti, ab, kw	1179451
#6	(control group): ti, ab, kw or (CON): ti, ab, kw or (active control): ti, ab, kw or (usual care): ti, ab, kw or (placebo): ti, ab, kw	581557
#7	#3 and #4 and#5 and #6	441

Appendix 2.3 Embase search results (*N*=241)

Step	Search strategy	Results
#1	'exercise'/exp	429601
#2	(statins): ti, ab, kw OR ' simvastatin ': ti, ab, kw OR ' rosuvastatin ': ti, ab, kw OR ' lovastatin ':ti, ab, kw OR ' fluvastatin ':ti, ab, kw OR ' atorvastatin ':ti, ab, kw OR ' whole-body vibration training ':ti, ab, kw OR ' WBV ': ti, ab, kw OR ‘training': ti, ab, kw OR 'aerobic ‘exercise': ti, ab, kw OR 'aerobic training': ti, ab, kw OR 'resistance training':ti, ab, kw OR 'strength training':ti, ab, kw OR 'moderate intensity continuous training':ti, ab, kw OR 'combined training':ti, ab, kw OR 'sprint interval training':ti, ab, kw OR 'high intensity interval training'. kw OR 'strength training’: ti, ab, kw OR 'moderate intensity continuous training’: ti, ab, kw OR 'combined training’: ti, ab, kw OR 'sprint interval training':ti, ab, kw OR 'high intensity interval training'.:ti, ab, kw OR 'physical activity'.:ti, ab, kw	901921
#3	#1 OR #2	1187115
#4	'Arterial stiffness’: ti, ab, kw OR 'PWV’: ti, ab, kw OR 'pulse wave velocity’: ti, ab, kw	31655
#5	'randomized': ti, ab, kw OR 'randomized controlled trial': ti, ab, kw OR 'placebo': ti, ab, kw OR 'rct': ti, ab, kw	1129810
#6	'Control group': ti, ab, kw OR 'CON': ti, ab, kw OR 'active control': ti, ab, kw OR 'usual care': ti, ab, kw OR 'placebo': ti, ab, kw	4002500
#7	#3 and #4 and#5 and #6	241

Appendix 2.4 EBSCO search results (*N*=98)

Step	Search strategy	Results
#1	AB statins OR AB simvastatin OR AB rosuvastatin OR AB lovastatin OR AB fluvastatin OR AB atorvastatin OR AB whole-body vibration training AB WBV OR AB physical activity OR AB training OR AB aerobic exercise OR AB aerobic training OR AB moderate intensity continuous training OR AB resistance training OR AB strength training OR AB combined training OR AB sprint interval	1593159
#2	D AB arterial stiffness OR AB pulse wave velocity OR AB PWV	14444
#3	AB randomized controlled trial OR AB randomized OR AB placebo OR AB RCT	1023801
#4	AB hypertension OR AB old people OR AB obesity OR AB type 2 diabetes OR AB metabolic syndrome OR AB postmenopausal women	5382327
#5	AB Control group OR AB CON OR AB active control OR AB usual care OR AB placebo	876899
#6	(AB Hypertension OR AB Hypercholesterolemia OR AB obesity OR AB Type 2 diabetes OR AB metabolic syndrome OR AB postmenopausal women OR AB Older persons over 60 years) AND (S1 AND S2 AND S3 AND S4 AND S5)	98

Appendix 3. Transformation formulas and the classifications of exercise interventions.

Appendix 3.1 Transformation formulas for estimating the mean and standard deviation.

(1) When calculating SD from M (confidence interval) for intervention or control group

a. the sample size in each group＞100

- SE= (upper limit-lower limit)/3.92
- SD=SE*N^ (1/2)

b. the sample size in each group≤100

- Input "tinv (1-0.95 , N_1-1)" in Microsoft Excel to obtain t
- SE= (upper limit-lower limit)/ t
- SD=SE*N^(1/2)

(2) When calculating SD from MD and P value between intervention or control group

a. reporting the exact p value

- Input "tinv (p , N_1+N_2-2)" in Microsoft Excel to obtain t
- SE=MD/t
- SD1=SE/N_1^(1/2)

b. reporting only significant levels (e.g. P＜0.05 or P＞0.05)

- The conservative calculation is to take the upper bound P. (e.g. P＜0.05 is replaced 0.05)

(3) When calculating M (SD) from the m (interquartile range) for intervention or control group

a. the sample size＞25, M=m;

the sample size≤25, M=$(a+2m+b)/4$

b. the sample size≤15, SD=$(((a-2m+b)^2/4+(b-a)^2)/12)$ ^ (1/2)

the sample size 15<n≤70, SD=$(b-a)/4$

the sample size＞70 , SD=$(b-a)/6$

(4) When calculating M (SD) from the m (interquartile range) for intervention or control group

a. M= $(q_1 + m + q_3)/3$

b. calculation of SD

- $Z=(0.25N_1+0.375)/(N1+0.25)$
- Input "norm. inv(1-z ,0 ,1)" obtain μ
- $SD=(q_3-q_1)/(2*\mu)$

Note:

M= mean; SE=standard error of mean; SD=standard deviation; N=sample size; MD= mean difference; m=median; a= minimum value; b = maximum value; q1= first quartile; q3= third quartile

Appendix 3.2 The classifications of exercise interventions

To compare the effects of different types of exercise , we classified exercise interventions into the following broad categories: interval training (INT , any intermittent traditional mode of interval training only , including single-component MIIT and HIIT such as walking , running , cycling , rowing , swimming , elliptical exercise , stepping exercise) , aerobic exercise (AE , aiming to improve cardiovascular fitness including walking , running , or cycling) , resistance exercise (RE , with the intent of increasing muscular strength and power using elastic bands , weight-machines) , combined training (CT , A combination of CET and RT). Different types of exercise were further classified and encoded according to the frequency, intensity, duration per session, and the length of intervention (see table 3.1).

Appendix 3.2.1 The classifications for exercise according to the frequency, intensity, duration per session, and the length of intervention.

Frequency	
Low	1-2 times/week
Moderate	3-4 times/week
High	3≥5 times/week
Duration per session	
Short	<30 minutes
Moderate-1	30-44 minutes
Moderate-2	45-59 minutes
long	≥60 minutes
Length of intervention	
Short	<13 weeks (3 months)

Moderate	14-25 weeks (3 months-6 months)
long	≥60 minutes (≥6 months)
Intensity	
Low	Interval training:<br>< 75%HR max/ 65% $\dot{V}$O2max/65% HRR<br>Aerobic exercise: < 60%HR max/ 60% $\dot{V}O_2max$<br>Resistance training: < 50% of 1 RM
Moderate	Interval training:<br>75%-90% HR max/65–85% $\dot{V}$O2max/65–85% HRR<br>Aerobic exercise: 60%-85% HR max/60–80% $\dot{V}O_2max$<br>Resistance training: 60–80% of 1 RM
High	Interval training:<br>>90% HR max/85% $\dot{V}$O2max/85% $\dot{V}$O2max<br>Aerobic exercise: >85% HR max/80% $\dot{V}O_2max$,<br>Resistance training: 80–100% of 1 RM

References: (Jarstad & Mamen , 2019; Morris et al. , 2017; Ramirez-Velez et al. , 2017; Zhou et al. , 2019)

Appendix 4. Characteristics of the included studies.

Study	Country	Characteristics of subject				Interventions information				
		Disease/ condition	Sample size (F)	age (mean [SD])	PWV Measure	Type	Duration	Supervised	Comparator	Outcomes
Guimarães 2010[1]	Brazil	Hypertension	65(42)	50(7.2)	Complior	INT: treadmill with intensity alternating between 50% (2 min) and 80% (1 min) of HRR	40min/session, 2session/week, 16weeks	YES	No exercise	②, ⑤, ⑥
Bouaziz 2019[2]	France	Sedentary aged 70 or over	60(44)	73.6(3.1)	DiaTecne s.r.l.	INT: 6*(4min at first ventilatory threshold (VT1) intensity + 1min at 40% of VT1 cycling exercise)	30min/session, 3session/week, 9.5weeks	YES	No exercise	②,⑤, ⑥
Aghaei 2019[3]	Iran	Stage 1 Hypertension	30(0)	48(3.2)	Enverdis	INT: 4 reps of 4min activity at 75% to 90% of Vo2peak interspersed with 4-min passive/active (15%-30% of VO 2peak) recovery.	32min/session, 2session/week, 8weeks	YES	No exercise	①, ⑤, ⑥
Zhuang 2023[4]	China	Older persons	82(49)	70(5.45)	Sphygmocor	INT: 3 daily cycling sessions of 40 mins 80% of HRmax	40min/day, 3days/week, 12 weeks	NO	No exercise	④, ⑤
Rodriguez 2017[5]	Spain	Metabolic syndrome	46(0)	53.5(8.9)	Sphygmocor	INT: four bouts of 4 min of pedaling at an intensity that elicited 90% of maxHR interspersed with 3-min active re-covery periods at 70% maxHR	45min/session, 3session/week, 24weeks	YES	No exercise	②, ⑤, ⑥
Deiseroth 2019[6]	Switzerla nd	Older persons	68(0)	59(7.5)	Sphygmocor	INT: 4 × 4 min at 80–90% of HRmax with 3 min active recovery at an intensity of 60–70% HRmax.	40min/session, 3session/week, 12weeks	YES	No exercise	③, ⑤, ⑥

Zhang 2022[7]	China	T2D	77(38)	57(7)	Complior	INT: 3 daily cycling sessions of 40 mins 70-90% of HRR	40min/day, 3days/week, 52 weeks	YES	No exercise	②, ⑤, ⑥
Kim 2017[8]	USA	Older persons	49(33)	65.2(1.3)	Sphygmocor	INT: 4*4 min at 90% HRpeak alternated by 3*3 min active recovery at 70% HRpeak.	40min/day, 3days/week, 12 weeks	YES	No exercise	②, ⑤, ⑥
Bellia 2017[9]	Italy	Overweight T2D	22(6)	57.4(7.3)	Sphygmocor	INT: 2-4 rep of 4 minutes' walk to 75-80% of the HRmax with active 3-minute recovery to 45-50% of the HRmax.	40min/day, 3days/week, 12 weeks	YES	Usual care	①, ⑤, ⑥
Way 2020[10]	Australia	Overweight T2D	34(15)	55.1(1.4)	Sphygmocor	INT: 1x4 min cycling at 90% peak oxygen consumption [V̇O2peak]	45min/day, 3days/week, 12 weeks	YES	Sham placebo	②, ⑤, ⑥
Adams 2017[11]	Canada	Cancer Survivors	63(0)	43.5(10.3)	Complior	INT: uphill treadmill walking/running, with 75% to 95% of VO2peak	40min/day, 3days/week, 12 weeks	YES	Usual care	②, ⑤, ⑥
Taha 2023[12]	Saudi Arabia	Obese Hypertension	60(60)	48.6(4.3)	Mobil-O-Graph	INT: cycling at 85-90% of PHR in ters with 3mins active recovery at 60 - 70% of PHR	45min/day, 3days/week, 12 weeks	YES	No exercise	④, ⑤, ⑥
Bouaziz 2013[13]	Brazil	Older persons	60(44)	73.6(2.9)	R wave of an ECG	INT: 6×4-min at first ventilatory threshold (VT1) intensity + 1-min at 40% of VT	30min/day, 2days/wee, 9.5 weeks	NO	No exercise	②, ⑤, ⑥
Millen 2013[14]	South Africa	Obese individuals	35(11)	44.3(6.4)	R wave of an ECG	AE: 60 and 75 % of VO2peak on a stationary bike and/or a treadmill	50min/day, ≥3days/week , 12 weeks	YES	No exercise	③, ⑤, ⑥

Kearney 2013[15]	Northern Ireland	Overweight adults	77(58)	45(7.1)	Intelesens	AE: walking on a treadmill at a constant self-selected speed. Increasing the incline by 2.5% at 3-min intervals until 80% of the age-predicted max HR.	30min/day, 5days/week, 16 weeks	YES	Stretching exercises	④,⑤, ⑥
Nualnim 2012[16]	USA	Older persons	43(32)	60.3(7.9)	VP-2000	AE: swimming ,40 to 45 min, at 70% to 75% of max HR	45min/day ,3-4days/week, 12weeks	YES	Stretching exercises	②, ⑤, ⑥
Zhou 2010[17]	China	Older Persons	35(0)	74.7(3.5)	SphymgmoCor	AE: treadmill training at 50–60 % of maxHR	45min/day, 3days/week, 12weeks	NO	No exercise	④,⑤, ⑥
Madden 2013[18]	Canada	Older persons hypertension, hyperlipidemia	52(22)	69(4.3)	Complior	AE: exercise treadmill (60min, at 60–75% of HRR)	60min/day, 3days/week, 8weeks	YES	Usual care	②, ⑤, ⑥
Horner 2015[19]	USA	Obese adolescents	81(40)	14.8(1.8)	Complior	AE: exercise on a treadmill, elliptical or stationary bike at 60–75% of VO2peak. RT: 10 whole body exercises (2 sets of 8–12 repetitions)	60min/day, 3days/week, 12 weeks	YES	No exercise	②, ⑤, ⑥
Huang 2018[20]	China	Obesity young persons	32(0)	20.7(1.1)	VP-1000	AE: cycling, walking/jogging, elliptical), 50min, at 60 – 85%HR	50min/day, 4days/week, 12weeks	YES	No exercise	④,⑤, ⑥
Shin 2015[21]	South Korea	Older persons	56(56)	65.2(5.6)	SphygmoCor	AE: Tai Chi exercise (included Twelve Movement, such as knee flexion; straight and extended head and trunk; combined rotation of head, and extremities)	60min/day, 1days/week, 12weeks	NO	usual care	②, ⑤, ⑥
Davis 2019[22]	USA	Overweight children	175(106)	9.7(0.7)	SphygmoCor	AE: aerobic activities (e.g., tag, jump rope) for 40 min each day, average daily HR > 140 beats/min.	55min/day, 3days/week, 16weeks	YES	Usual care	②, ⑤, ⑥

Chen 2018[23]	China	Older persons	42(34)	66.5(3.2)	VP-1000	AE: composed of 5 minutes of warm-up, 45 minutes ofconditioning, and 5 minutes of cooldown at 50%-60% of the peak oxygen uptake (VO2peak)	60min/day, 6days/week, 24weeks	YES	No exercise	④,⑤, ⑥
Madden 2009[24]	Canada	Older persons	40(19)	71.4(0.7)	transducer measures	AE: exercise on a treadmill and a cycle ergometer, with 60–75% HRR	60min/day, 3days/week, 12weeks	YES	No exercise	②, ⑤, ⑥
0'Connor 2017[25]	UK	KTR	46(19)	51.8(12.5)	Skidmore	AE: individually tailored exercise training	30min/day, 3days/week, 12weeks	YES	usual care	②, ⑤, ⑥
Jaime 2017[26]	USA	Older persons	19(19)	66(2.5)	SphygmoCor	RT: consisted of leg press, leg extension, leg flexion, and calf raises exercises at 40% of the 1RM	30min/day, 3 days/week, 12 weeks	YES	No exercise	②, ⑤, ⑥
Croymans 2013[27]	USA	Obese young men	36(0)	21.5(0.8)	SphygmoCor	RT: 3 phase 2-3 sets, with 8–15 repetitions of each exercise, at 100% approximated 12–15 RM or 6-8RM	60min/day, 3 days/week, 12 weeks	YES	No exercise	②, ⑤, ⑥
DeVallance 2016[28]	USA	Metabolic syndrome	29(20)	51(14.5)	SphygmoCor	RT: six exercises (leg press, chest press, lat pull down, leg curl, shoulder press, and leg extension) on body master weight.	45min/day, 3 days/week, 8 weeks	YES	No exercise	②, ⑤, ⑥
Greenwood 2015[29]	UK	KTR	60(26)	54.3(10.5)	Complior	RT: high-intensity resistance training at 80% one-rep max.	40min/day, 2days/week, 12weeks	YES	usual care	②, ⑤, ⑥
Beck2013[30]	USA	Hypertension	43(13)	21.1(0.7)	SphygmoCor	RT: 3 min of walking at 65% of maxHR and 2 min running at incline at 85% of maxHR for a total of nine 5-minute intervals.	60min/day, 3days/week, 8 weeks	NO	No exercise	②, ⑤, ⑥

Fernandez 2018[31]	USA	Obese women	11(11)	23.5(4.2)	SphygmoCor	RT: 50min ,3 sets of 10 reps at 70-85% 1 RM.	50min/day, 3days/week, 3 weeks	YES	Usual care	①, ⑤, ⑥
Jaime 2019[32]	USA	postmenopausal women	20(20)	65.6(3.2)	VP-2000	RT: leg press, leg extension, leg flexion, and calf raise exercises initially prescribed at 40% of the 1RM, with the max goal of 15 reps.	35min/day, 3days/week, 12 weeks	YES	Usual care	②, ⑤, ⑥
Ramírez 2020[33]	Spain	Overweight adults	57(34)	40.78(7.1)	Arteriograph	RT: 12–15 reps per set of 6 exercises, A 60-second recovery at 50–70%1RM	40min/day, 3days/week, 12 weeks	NO	Usual care	①
Wong 2017[34]	USA	Obese Adolescent Girls	30(30)	15.3(1.2)	SphygmoCor	CT: Combined exercise consisted of 20 minutes of various resistant band exercises	60min/day, 3days/week, 12 weeks	YES	No exercise	④
Lee 2015[35]	South Korea	Chronic Poststroke Hemiparesis	26(0)	64(6.5)	SphygmoCor	CT: a 5-min warm-up, a 10-min stretching exercise, a 20-minresistance exercise, a 20-min aerobic exercise, and a 5-min cool down.	50min/day, 3days/week, 16 weeks	YES	No exercise	④, ⑤, ⑥
Shiotsu 2018[36]	Japan	older men	40(0)	70.5 (3.5)	VaSera	CT: AE (cycling at 60% of HR) + RT (3 sets of 8–12 reps for 5 different exercises, 70–80% of 1RM)	45min/day, 2days/week, 10 weeks	YES	No exercise	②
Stewart 2005[37]	USA	Older Persons	104(53)	63.6(5.7)	SphygmoCor	CT: RT (2 sets of 10-15 rep at 50% 1RM) +AE (running, 45min at 60% to 90% of maxHR)	90min/day, 3days/week, 24 weeks	NO	Usual care	①, ⑤, ⑥
Park 2020[38]	Korea	Obese Older Men	20(0)	68.8(0.9)	Complior	CT: RT (elastic-band ,3 sets of 10-15 reps at 60-70% 1RM ,30-40min) +AE (running and bicycle ,60min at 60%–70% of HRmax	90min/day, 3 days/week, 12 weeks	YES	No exercise	④, ⑤, ⑥

Son 2016[39]	South Korea	Postmenopausal women with Hypertension	20(20)	75.3(3.5)	SphygmoCor	CT: RT (20 min of various resistant band exercises) +AE (30 minutes of walking, 40% to 50% HR in 1-4 week, 60% to 70% HR)	70min/day, 3 days/week, 12 weeks	NO	Usual care	④, ⑤, ⑥
Dobrosielski 2012[40]	USA	T2D	140(81)	56.5(6)	SphygmoCor	CT: RT (2 sets of 7 exercises at 10 to 15 reps per exercise at 50 % of 1-rep max) +AE (45min, at 60 % to 90 % of maxHR)	70min/day, 3 days/week, 26 weeks	YES	No exercise	②,⑤, ⑥
Loimaala 2008[41]	Finland	Type 2 Diabetes	48(0)	53.8(5.6)	ECG	CT: AE (jogging or walking, at 65% to 75% VO2max) +RT (12 different resistance training, 3 to 4 sets with 10 to 12 reps at 60%-80%1RM)	30min/day, 4 days/week, 96 weeks	YES	Usual care	②, ⑤
Figueroa 2014[42]	USA	Postmenopausal women	25(25)	55.8(0.9)	VP-2000	Including four leg exercises standing on a WBV platform	10min/day, 3days/week ,12weeks	YES	No exercise	②,⑤, ⑥
Figueroa 2012[43]	USA	Obese women	10(10)	20(0.9)	VP-2000	including four leg exercises standing on a WBV platform	10min/day, 3days/week ,6weeks	YES	No exercise	④,⑤, ⑥
Figueroa 2015[44]	USA	Obese postmenopausal women	36(36)	57.3(3.5)	VP-2000	including six leg exercises standing on a WBV platform	10min/day, 3days/week ,12weeks	YES	No exercise	④,⑤, ⑥
Alvarez 2017[45]	USA	Obese women	38(38)	20.5(4.47)	VP-2000	Including six leg exercises standing on a WBV platform	10min/day, 3days/week ,6weeks	YES	No exercise	②,⑤, ⑥
Yule 2016[46]	New Zealand	Chronic Stroke	6(2)	50.5(14.5)	SphygmoCor	Including six leg exercises standing on a WBV platform	5min/day, 3days/week ,4weeks	YES	No exercise	②,⑤, ⑥

Raison 2002[47]	France	Hypertension Hypercholesterolemia	33(12)	56.7(10.8)	Complior	Atorvastatin 10mg once daily	12weeks	NO	Placebo-control	②,⑤, ⑥
Kanaki 2013[48]	Greece	Hypertension Hypercholesterolemia	50(26)	59.3(10)	sphygmoCor	Atorvastatin 10mg once daily	26weeks	NO	Placebo-control	②,⑤, ⑥
Orr 2009[49]	USA	Obese older Adults	26(15)	54(2.65)	ECG	Atorvastatin 80mg once daily	12weeks	NO	Placebo-control	②,⑤, ⑥
Pirro 2007[50]	Italy	Hypercholesterol emia	71(33)	57(15.72)	ECG	Rosuvastatin 10mg once daily	4weeks	NO	Active-control	①,⑤, ⑥
Igase 2012[51]	Japan	postmenopausal women with dyslipidemia	31(31)	67.1(5.8)	FORM/ABI	Rosuvastatin 2.5mg once daily	12weeks	NO	Active-Control	④,⑤, ⑥
Grigoropoulou 2019[52]	Greece	T2DM Dyslipidemia	79(48)	59.5(8.54)	SphygmoCor	Atorvastatin 10mg once daily	48weeks	NO	Active-control	②,⑤, ⑥
Qi 2007[53]	China	Hypertension	87(39)	57.4(5.62)	Complior	Atorvastatin 10mg once daily	12weeks	NO	Placebo-control	②,⑤, ⑥
Chen 2009[54]	China	Hypertension	104(37)	55.1(11.2)	VP-000	Atorvastatin 20mg once daily	24weeks	NO	Active-control	②,⑤, ⑥
Zhang 2003[55]	China	Hypertension	30(13)	64.9(9.23)	Complior	Fluvastatin 40mg once daily	12weeks	NO	Placebo-control	②,⑤, ⑥

Oh 2014[56]	USA	Hypercholesterolemia	56(17)	50.4(8.35)	VP-2000	Rosuvastatin 10mg once daily	8weeks	NO	Active-control	④,⑤, ⑥
Mukherjee 2008[57]	Indian	T2D	57(31)	Lack	VP-2000	Atorvastatin 10mg once daily	24weeks	NO	Placebo-control	④,⑤, ⑥
Ichihara 2005[58]	Japan	Hypertension	85(14)	60(2.5)	ECG	Fluvastatin 20mg once daily; Pravastatin 10mg once daily; Simvastatin 5mg once daily	48weeks	NO	Placebo-control	④,⑤, ⑥

Note.①: Aortic pulse wave velocity; ②: Carotid-femoral pulse wave velocity; ④: Brachial arteries pulse wave velocity; ⑤: systolic blood pressure; ⑥: diastolic blood pressure.

Abbreviation: ECG: Electro-Chemical Grinding; T2D: Type 2 Diabetes; KTR: Kidney Transplant Recipients.

Appendix 5. Risk of bias assessment.

Appendix 5.1 Risk of bias assessment table

Study	Random sequence generation	Allocation concealment	blinding of outcome assessors	incomplete outcome	selective outcome reporting	other risks of bias	Risk category
Guimarães 2010	Low	Unclear	Low	High	Unclear	Low	Moderate
Bouaziz 2019	Low	Unclear	Low	Low	Unclear	Low	Low
Aghaei 2019	Unclear	Unclear	Low	Unclear	Low	Low	Low
Zhuang 2023	Unclear	Unclear	Unclear	High	Unclear	Low	Moderate
Rodriguez 2017	Unclear	Unclear	Low	Low	Unclear	Low	Low
Deiseroth 2019	Low	Unclear	Unclear	Low	Unclear	Low	Low
Zhang 2022	High	Unclear	Unclear	High	Unclear	Low	High
Kim 2017	Low	Low	Low	High	Unclear	Low	Moderate
Bellia 2017	Unclear	Unclear	Unclear	Low	Low	High	Moderate
Way 2020	Low	Low	Low	Low	Unclear	Low	Low
Adams 2017	Low	Unclear	Unclear	Low	Unclear	Low	Low
Taha 2023	Low	Low	Low	Low	Unclear	Low	Low
Wong 2017	Unclear	Unclear	Unclear	Low	Unclear	High	High
Lee 2015	Low	Low	Low	Low	Low	High	Moderate
Shiotsu 2018	Unclear	Unclear	Low	Low	Unclear	Low	Low
Stewart 2005	Unclear	Unclear	Low	Low	Unclear	Low	Low
Park 2020	Low	Low	Low	Low	Unclear	High	Moderate
Bouaziz 2013	Unclear	Unclear	Low	Low	Low	High	Moderate
Son 2016	Unclear	Unclear	Low	Low	Unclear	High	High
Dobrosielski 2012	Unclear	Unclear	Low	Low	Unclear	Low	Low
Greenwood 2015	Low	Low	Unclear	Low	Unclear	High	Moderate
Loimaala 2008	Unclear	Unclear	Low	Low	Unclear	Low	Low
Jaime 2017	Unclear	Unclear	Low	Low	Low	Low	Low
Croymans 2013	Low	Low	Low	Low	Unclear	Low	Low
DeVallance 2016	Low	Low	Low	Low	Unclear	Low	Low
Zhou 2010	Low	Low	Low	Low	Unclear	Low	Low
Horner 2015	Unclear	Unclear	Low	Low	Unclear	Low	Low
Beck 2013	Unclear	Unclear	Unclear	Low	Unclear	Low	Moderate
Fernandez 2018	Unclear	Unclear	Unclear	Low	Unclear	High	High
Jaime 2019	Unclear	Unclear	Low	Low	Unclear	Low	Low
Ramírez 2020	Low	Low	Unclear	High	Low	Low	Moderate
Millen 2013	Unclear	Unclear	Low	Low	Low	Low	Low
Kearney 2013	Unclear	Low	Low	High	Unclear	Low	Moderate
Huang 2018	Unclear	Unclear	Unclear	Low	Unclear	Low	Moderate
Nualnim 2012	Unclear	Unclear	Low	Unclear	Low	Low	Low

Chen 2018	Unclear	Unclear	Unclear	Low	Unclear	High	High
Madden 2013	Low	Low	Low	Low	Unclear	Unclear	Low
Madden 2009	Unclear	Unclear	Low	Unclear	Low	Low	Low
Shin 2015	Unclear	Unclear	Low	High	Unclear	Low	Moderate
Davis 2019	Low	Low	Low	Low	Unclear	Low	Low
0’Connor 2017	Unclear	Unclear	Low	Unclear	Unclear	Low	Low
Figueroa 2013	Low	Low	Low	Low	Low	Unclear	Low
Figueroa 2012	Low	Unclear	Low	Low	Low	Unclear	Low
Figueroa 2014	Low	Low	Low	High	Low	Low	Moderate
Alvarez 2017	Low	Low	Low	Low	Low	Low	Low
Yule 2016	Low	Unclear	Low	Low	Unclear	Low	Low
Raison 2002	Low	Unclear	Low	Low	Unclear	Unclear	Low
Kanaki 2013	Low	Unclear	Low	Low	Unclear	Unclear	Low
Orr 2009	Low	Low	Low	Low	Unclear	Unclear	Low
Pirro 2006	Low	Unclear	Low	Unclear	Unclear	Unclear	Moderate
Igase 2012	Low	Unclear	Low	Unclear	Unclear	Low	Low
Grigoropoulou 2018	High	High	Low	Low	Unclear	Low	High
Qi 2007	Unclear	Unclear	Low	Low	Unclear	Low	Low
Chen 2009	Unclear	Unclear	Low	Low	Unclear	Low	Low
Zhang 2003	Unclear	Unclear	Low	Low	Low	Low	Low
Oh 2014	Unclear	Unclear	Low	Low	Unclear	Low	Low
Mukherjee 2008	Low	Low	Low	Low	Unclear	Low	Low
Ichihara 2005	Low	Unclear	Low	Low	Unclear	Low	Low

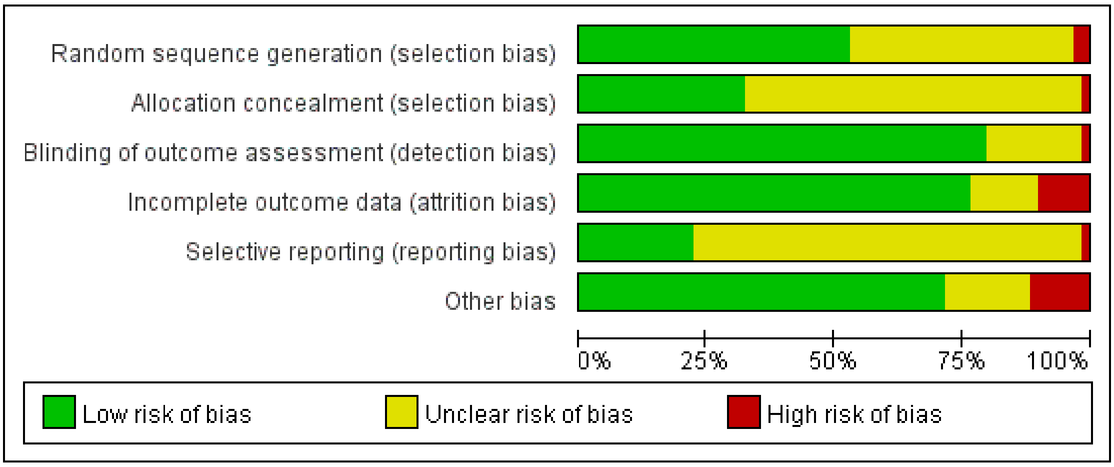

Appendix 5.2 The overall risk of bias for all included studies

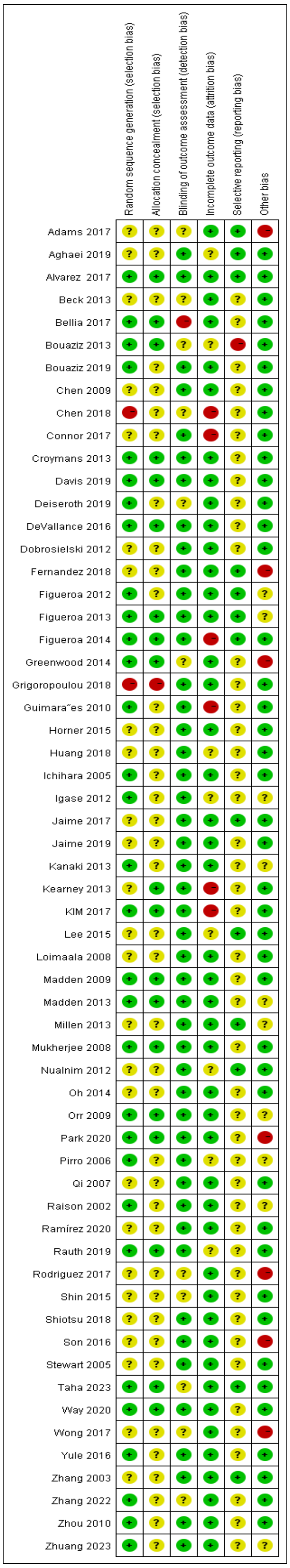

Appendix 5.3 The risk of bias for each study

Appendix 6. Funnel plot of Pulse Wave Velocity, Systolic Blood Pressure, and Diastolic Blood Pressure in pairwise meta-analysis.

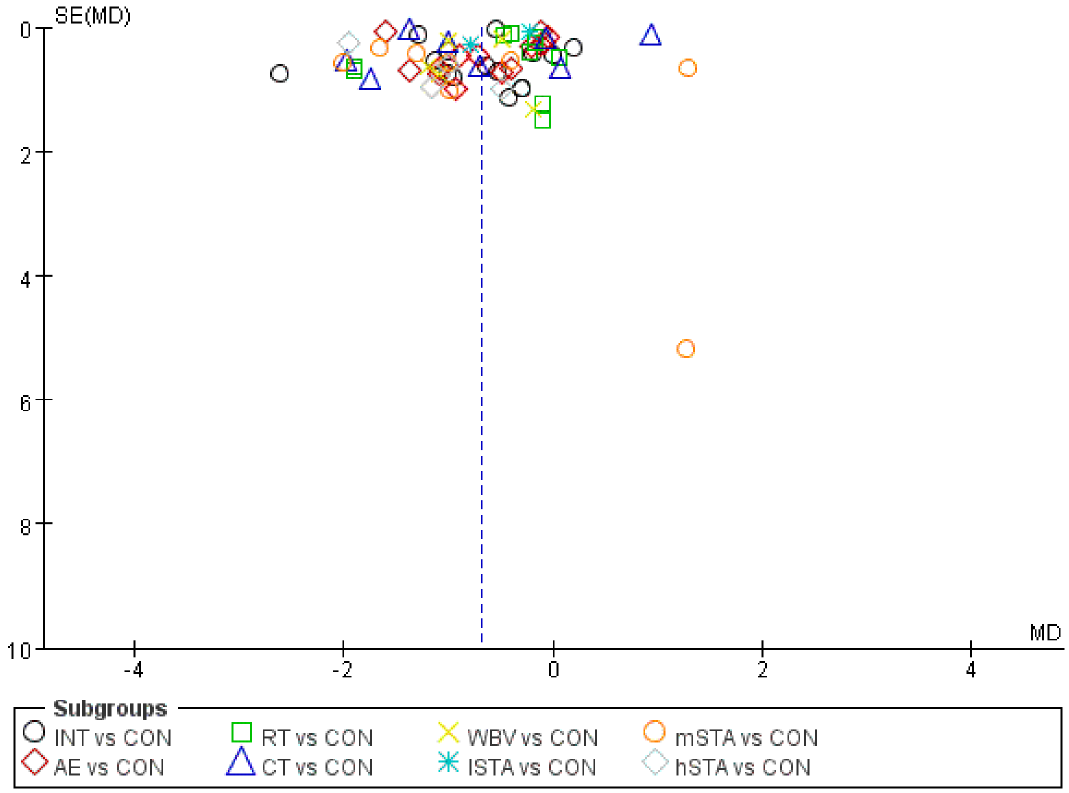

Appendix 6.1 Funnel plot of Pulse Wave Velocity in pairwise meta-analysis.

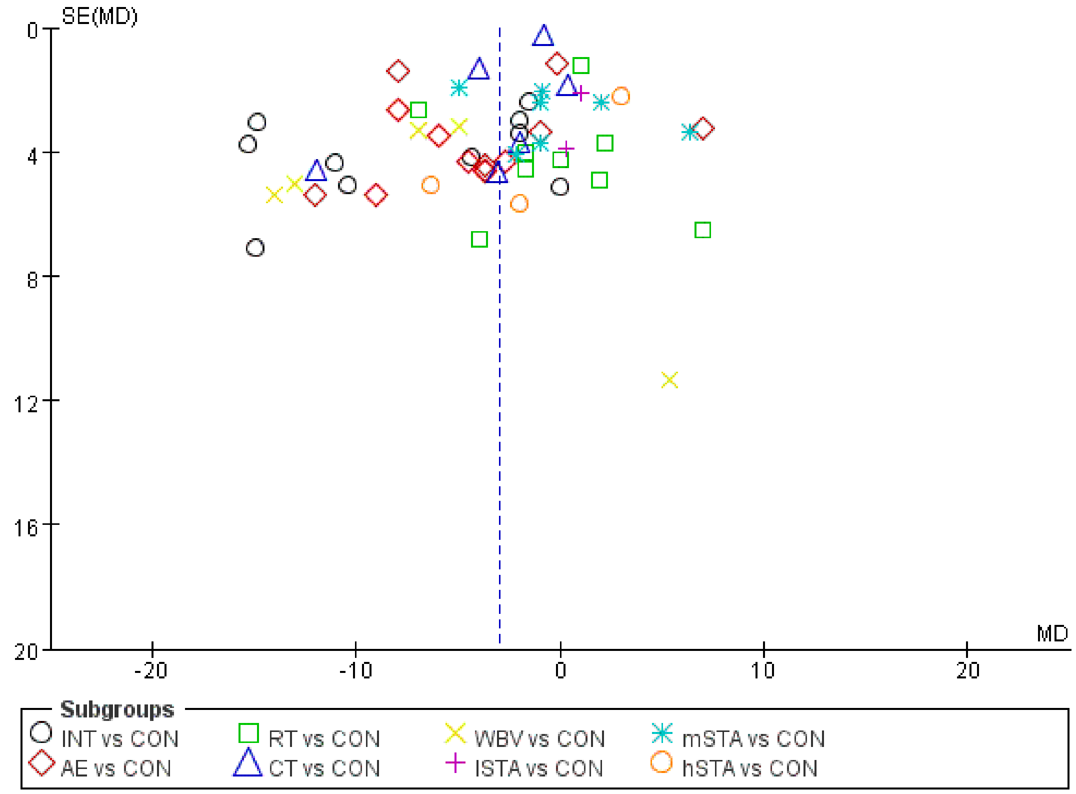

Appendix 6.2 Funnel plot of Systolic Blood Pressure in pairwise meta-analysis.

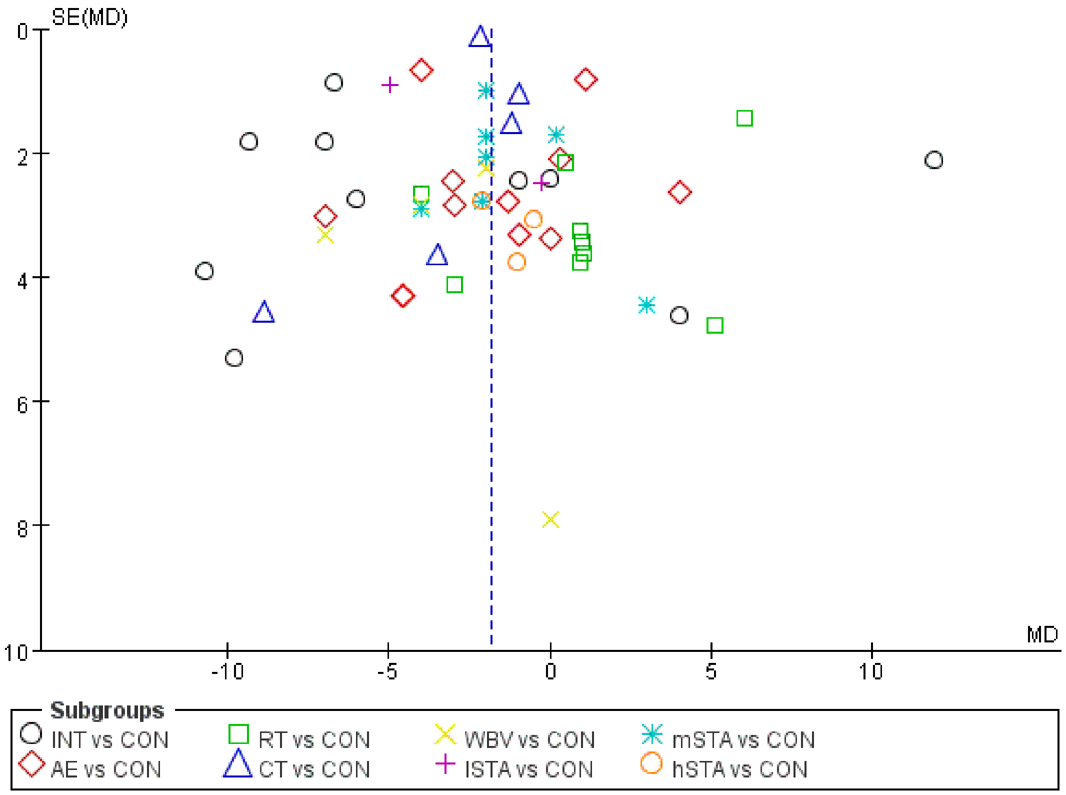

Appendix 6.3 Funnel plot of Diastolic Blood Pressure in pairwise meta-analysis.

Appendix 7. Forest plots for Pulse Wave Velocity, Systolic Blood Pressure, and Diastolic Blood Pressure.

Study or Subgroup	Experimental Mean	Experimental SD	Experimental Total	Control Mean	Control SD	Control Total	Weight	Mean Difference IV, Random, 95% CI
**1.1.1 INT vs CON**								
Adams 2017	-0.74	1.75	33	0.26	1.3	26	1.8%	-1.00 [-1.78, -0.22]
Aghaei 2019	-0.76	2.08	10	0.19	1.47	10	0.9%	-0.95 [-2.53, 0.63]
Bellia 2017	-2.6	2.19	11	0.01	1.29	11	1.0%	-2.61 [-4.11, -1.11]
Bouaziz 2013	-0.49	2.75	30	0.04	2.99	30	1.0%	-0.53 [-1.98, 0.92]
Bouaziz 2019	-0.32	4.38	27	0.11	4.18	29	0.5%	-0.43 [-2.68, 1.82]
Chen 2018	-0.1	1.15	38	-0.3	1.6	30	1.9%	0.20 [-0.48, 0.88]
Deiseroth 2019	-0.37	1.01	16	0.26	1.91	11	1.2%	-0.63 [-1.86, 0.60]
Guimara˜es 2010	-0.51	1.58	18	0.5	2.47	14	1.0%	-1.01 [-2.50, 0.48]
KIM 2017	-0.27	0.04	14	0.27	0.13	16	2.6%	-0.54 [-0.61, -0.47]
Ramírez 2020	-1.14	0.5	30	0.15	0.5	30	2.5%	-1.29 [-1.54, -1.04]
Rodriguez 2017	-0.33	0.85	12	0.8	1.6	11	1.4%	-1.13 [-2.19, -0.07]
Taha 2023	0.8	4.37	39	1.1	4.41	38	0.7%	-0.30 [-2.26, 1.66]
Way 2020	-0.6	2.01	39	-0.4	1.81	39	1.7%	-0.20 [-1.05, 0.65]
Zhang 2022	0.8	4.37	39	1.1	4.41	38	0.7%	-0.30 [-2.26, 1.66]
Zhuang 2023	0.01	1.11	13	0.01	1.15	12	1.6%	0.00 [-0.89, 0.89]
**Subtotal (95% CI)**			**369**			**345**	**20.4%**	**-0.69 [-1.03, -0.35]**
Heterogeneity: Tau^2 = 0.19; Chi^2 = 48.94, df = 14 (P < 0.00001); I^2 = 71%								
Test for overall effect: Z = 3.96 (P < 0.0001)								
**1.1.2 AE vs CON**								
Beck 2013	-0.05	0.71	13	0.03	0.66	15	2.2%	-0.08 [-0.59, 0.43]
Chen 2018	-0.95	2.38	23	0.43	1.97	15	1.1%	-1.38 [-2.77, 0.01]
Connor 2017	-0.6	1.51	13	0.5	2.3	20	1.1%	-1.10 [-2.40, 0.20]
Davis 2019	-0.02	1.03	90	0.04	1.02	85	2.4%	-0.06 [-0.36, 0.24]
Greenwood 2014	-0.6	2.01	13	0.5	2.3	20	1.0%	-1.10 [-2.59, 0.39]
Guimara˜es 2010	-0.24	1.75	16	0.26	1.91	11	1.0%	-0.50 [-1.92, 0.92]
Horner 2015	-0.6	1.87	27	0.13	1.2	16	1.6%	-0.73 [-1.65, 0.19]
Huang 2018	-0.7	1.47	16	0.2	1.15	16	1.6%	-0.90 [-1.81, 0.01]
Kearney 2013	-2.15	0.26	25	-0.54	0.27	25	2.6%	-1.61 [-1.76, -1.46]
Madden 2009	-0.21	0.25	18	-0.08	0.25	18	2.6%	-0.13 [-0.29, 0.03]
Millen 2013	-1.21	3.5	25	-0.27	3.63	27	0.7%	-0.94 [-2.88, 1.00]
Nualnim 2012	0.27	0.91	19	0.39	0.84	16	2.1%	-0.12 [-0.70, 0.46]
Shin 2015	-0.2	0.92	24	0.01	1.14	19	2.0%	-0.21 [-0.84, 0.42]
Zhou 2010	-0.94	1.52	29	-0.53	2.24	14	1.1%	-0.41 [-1.71, 0.89]
**Subtotal (95% CI)**			**351**			**317**	**23.0%**	**-0.61 [-1.12, -0.10]**
Heterogeneity: Tau^2 = 0.72; Chi^2 = 222.00, df = 13 (P < 0.00001); I^2 = 94%								
Test for overall effect: Z = 2.34 (P = 0.02)								
**1.1.3 RT vs CON**								
Beck 2013	-0.14	0.7	15	0.03	0.66	15	2.2%	-0.17 [-0.66, 0.32]
Connor 2017	-1.4	1.64	20	0.5	2.3	20	1.2%	-1.90 [-3.14, -0.66]
Croymans 2013	-0.1	0.36	28	0.3	0.2	8	2.5%	-0.40 [-0.59, -0.21]
DeVallance 2016	-0.13	0.4	13	0.04	0.41	16	2.5%	-0.17 [-0.47, 0.13]
Fernandez 2018	0.09	0.82	6	0.04	0.79	5	1.5%	0.05 [-0.90, 1.00]
Greenwood 2014	-1.4	1.64	13	0.5	2.3	20	1.1%	-1.90 [-3.25, -0.55]
Horner 2015	-0.1	1.29	23	0.13	1.2	16	1.8%	-0.23 [-1.02, 0.56]
Jaime 2017	-0.1	2.75	11	0.01	3.54	8	0.3%	-0.11 [-3.05, 2.83]
Jaime 2019	-0.1	2.42	12	0.01	2.87	8	0.5%	-0.11 [-2.52, 2.30]
Ramírez 2020	-0.2	0.4	12	0.27	0.13	16	2.5%	-0.47 [-0.71, -0.23]
**Subtotal (95% CI)**			**153**			**132**	**16.1%**	**-0.38 [-0.59, -0.17]**
Heterogeneity: Tau^2 = 0.03; Chi^2 = 14.94, df = 9 (P = 0.09); I^2 = 40%								
Test for overall effect: Z = 3.57 (P = 0.0004)								
**1.1.4 CT vs CON**								
Dobrosielski 2012	0.08	3.77	70	0.01	4.18	70	1.1%	0.07 [-1.25, 1.39]
Lee 2015	-0.78	1.74	14	0.97	2.44	12	0.8%	-1.75 [-3.40, -0.10]
Loimaala 2008	0.6	2.24	24	1.3	2.24	24	1.2%	-0.70 [-1.97, 0.57]
Park 2020	-0.04	0.42	10	0.03	0.42	10	2.4%	-0.07 [-0.44, 0.30]
Shiotsu 2018	-0.99	0.16	15	0.38	0.13	10	2.6%	-1.37 [-1.48, -1.26]
Son 2016	-0.78	1.35	10	1.19	1.15	10	1.4%	-1.97 [-3.07, -0.87]
Stewart 2005	1.11	0.73	51	0.17	0.57	53	2.5%	0.94 [0.69, 1.19]
Wong 2017	-1	0.65	15	0.01	0.75	15	2.2%	-1.01 [-1.51, -0.51]
**Subtotal (95% CI)**			**209**			**204**	**14.1%**	**-0.69 [-1.62, 0.24]**
Heterogeneity: Tau^2 = 1.58; Chi^2 = 295.64, df = 7 (P < 0.00001); I^2 = 98%								
Test for overall effect: Z = 1.45 (P = 0.15)								
**1.1.5 WBV vs CON**								
Alvarez 2017	-0.6	0.85	25	-0.1	0.35	13	2.4%	-0.50 [-0.88, -0.12]
Figueroa 2012	-0.9	0.35	5	0.11	0.35	5	2.3%	-1.01 [-1.44, -0.58]
Figueroa 2013	-1.3	1.91	13	-0.1	1.25	12	1.2%	-1.20 [-2.46, 0.06]
Figueroa 2014	-1.1	1.94	12	0	1.73	12	1.0%	-1.10 [-2.57, 0.37]
Yule 2016	-0.4	1.97	3	-0.2	1.13	3	0.4%	-0.20 [-2.77, 2.37]
**Subtotal (95% CI)**			**58**			**45**	**7.2%**	**-0.75 [-1.03, -0.48]**
Heterogeneity: Tau^2 = 0.00; Chi^2 = 3.90, df = 4 (P = 0.42); I^2 = 0%								
Test for overall effect: Z = 5.40 (P < 0.00001)								
**1.1.7 lSTA vs CON**								
Ichihara 2005	-0.22	0.21	26	0.01	0.24	25	2.6%	-0.23 [-0.35, -0.11]
Igase 2012	-0.38	0.91	22	0.41	0.9	20	2.1%	-0.79 [-1.34, -0.24]
**Subtotal (95% CI)**			**48**			**45**	**4.7%**	**-0.44 [-0.98, 0.09]**
Heterogeneity: Tau^2 = 0.12; Chi^2 = 3.82, df = 1 (P = 0.05); I^2 = 74%								
Test for overall effect: Z = 1.63 (P = 0.10)								
**1.1.8 mSTA vs CON**								
Grigoropoulou 2018	0.97	13.82	12	-0.29	10.88	11	0.0%	1.26 [-8.86, 11.38]
Ichihara 2005	-1.7	1.87	25	0.3	2.17	25	1.3%	-2.00 [-3.12, -0.88]
Kanaki 2013	-1.7	1.73	35	-0.4	2.01	36	1.7%	-1.30 [-2.17, -0.43]
Mukherjee 2008	-0.8	2.56	46	0.2	2.45	33	1.3%	-1.00 [-2.12, 0.12]
Oh 2014	-0.31	3.09	42	-1.6	3.03	45	1.2%	1.29 [0.00, 2.58]
Pirro 2006	-0.5	1.75	41	-0.1	1.9	15	1.4%	-0.40 [-1.50, 0.70]
Qi 2007	-1.54	3.29	29	-0.55	4.25	28	0.7%	-0.99 [-2.97, 0.99]
Raison 2002	-1.25	1.31	21	0.41	0.9	20	1.9%	-1.66 [-2.35, -0.97]
**Subtotal (95% CI)**			**251**			**213**	**9.4%**	**-0.92 [-1.65, -0.19]**
Heterogeneity: Tau^2 = 0.63; Chi^2 = 20.28, df = 7 (P = 0.005); I^2 = 65%								
Test for overall effect: Z = 2.47 (P = 0.01)								
**1.1.9 hSTA vs CON**								
Chen 2009	-1.64	1.36	16	-0.47	2.85	10	0.7%	-1.17 [-3.06, 0.72]
Ichihara 2005	-1.3	2.88	46	-0.3	2.91	47	1.3%	-1.00 [-2.18, 0.18]
Orr 2009	-0.7	2.36	15	-0.2	2.98	15	0.7%	-0.50 [-2.42, 1.42]
Zhang 2003	-1.54	0.77	22	0.41	0.9	20	2.2%	-1.95 [-2.46, -1.44]
**Subtotal (95% CI)**			**99**			**92**	**4.8%**	**-1.50 [-2.16, -0.84]**
Heterogeneity: Tau^2 = 0.13; Chi^2 = 4.08, df = 3 (P = 0.25); I^2 = 27%								
Test for overall effect: Z = 4.46 (P < 0.00001)								
**Total (95% CI)**			**1538**			**1393**	**100.0%**	**-0.68 [-0.86, -0.49]**
Heterogeneity: Tau^2 = 0.34; Chi^2 = 740.62, df = 65 (P < 0.00001); I^2 = 91%								
Test for overall effect: Z = 7.21 (P < 0.00001)								
Test for subgroup differences: Chi^2 = 13.79, df = 7 (P = 0.05), I^2 = 49.3%								

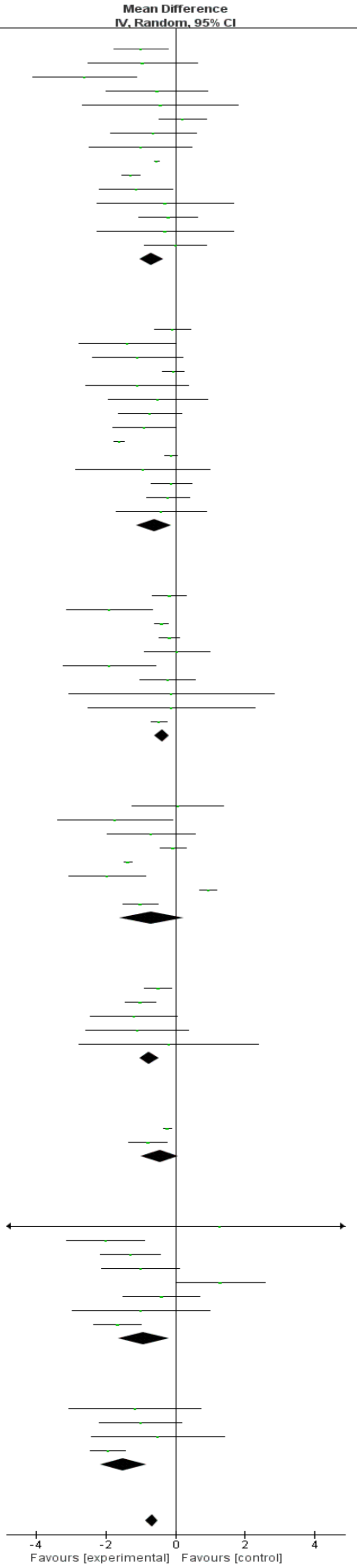

Appendix 7.1 forest plot of Pulse Wave Velocity.

Study or Subgroup	Experimental Mean	Experimental SD	Experimental Total	Control Mean	Control SD	Control Total	Weight	Mean Difference IV, Random, 95% CI
**1.2.1 INT vs CON**								
Adams 2017	-1.5	9.96	35	0.01	8.99	27	2.6%	-1.51 [-6.24, 3.22]
Aghaei 2019	-7.89	12.78	10	2.49	9.79	10	1.1%	-10.38 [-20.36, -0.40]
Bellia 2017	3	10.91	11	3	13.02	11	1.1%	0.00 [-10.04, 10.04]
Bouaziz 2013	-11.1	9.61	30	3.7	13.73	30	2.1%	-14.80 [-20.80, -8.80]
Bouaziz 2019	-11.65	19.55	30	3.26	33.6	30	0.6%	-14.91 [-28.82, -1.00]
Deiseroth 2019	1	13.11	38	3	14.53	30	1.8%	-2.00 [-8.66, 4.66]
Guimara˜es 2010	-2	8.54	16	0.01	8.54	16	2.1%	-2.01 [-7.93, 3.91]
Rodriguez 2017	-9	15.13	23	2	14.42	23	1.3%	-11.00 [-19.54, -2.46]
Taha 2023	-17.96	19.44	30	-2.64	7.07	30	1.6%	-15.32 [-22.72, -7.92]
Zhang 2022	-7.1	17.27	39	-2.8	19.58	38	1.4%	-4.30 [-12.55, 3.95]
**Subtotal (95% CI)**			**262**			**245**	**15.6%**	**-7.18 [-11.21, -3.14]**
Heterogeneity: Tau² = 26.09; Chi² = 25.98, df = 9 (P = 0.002); I² = 65%								
Test for overall effect: Z = 3.48 (P = 0.0005)								
**1.2.2 AE vs CON**								
Beck 2013	-11	7.21	13	-3	6.71	15	2.4%	-8.00 [-13.19, -2.81]
Connor 2017	-1.9	13.44	13	1.8	12.02	20	1.2%	-3.70 [-12.71, 5.31]
Davis 2019	0.1	7.64	90	0.3	7.65	85	3.7%	-0.20 [-2.47, 2.07]
Greenwood 2014	-1.9	13.44	13	1.8	12.02	20	1.2%	-3.70 [-12.71, 5.31]
Guimara˜es 2010	-1	8.54	11	0.01	8.54	16	1.9%	-1.01 [-7.57, 5.55]
Horner 2015	-3.42	17.04	27	1.13	11.16	16	1.4%	-4.55 [-12.99, 3.89]
Huang 2018	3.9	9.06	16	-3.1	9.1	16	1.9%	7.00 [0.71, 13.29]
Madden 2009	-10	5	18	-2	3	18	3.5%	-8.00 [-10.69, -5.31]
Madden 2013	-14	20	25	-2	18.73	27	1.0%	-12.00 [-22.55, -1.45]
Millen 2013	-7	10.15	19	-1	10.15	16	1.8%	-6.00 [-12.75, 0.75]
Nualnim 2012	-9	17.66	24	0.01	17.43	19	1.0%	-9.01 [-19.56, 1.54]
Shin 2015	-0.9	10.5	29	1.8	14.4	14	1.4%	-2.70 [-11.16, 5.76]
Zhou 2010	-4	12.91	17	-0.3	13.09	18	1.3%	-3.70 [-12.32, 4.92]
**Subtotal (95% CI)**			**315**			**300**	**23.7%**	**-3.92 [-6.84, -1.00]**
Heterogeneity: Tau² = 16.06; Chi² = 37.17, df = 12 (P = 0.0002); I² = 68%								
Test for overall effect: Z = 2.63 (P = 0.008)								
**1.2.3 RT vs CON**								
Beck 2013	-10	7.75	15	-3	6.71	15	2.4%	-7.00 [-12.19, -1.81]
Connor 2017	0.1	13.3	20	1.8	12.02	20	1.5%	-1.70 [-9.56, 6.16]
Croymans 2013	-5	2.42	28	-6	3.14	8	3.7%	1.00 [-1.35, 3.35]
DeVallance 2016	-1	10.87	13	-1	12	16	1.4%	0.00 [-8.34, 8.34]
Fernandez 2018	1.79	6.96	6	-0.07	8.86	5	1.1%	1.86 [-7.70, 11.42]
Greenwood 2014	0.1	13.3	13	1.8	12.02	20	1.2%	-1.70 [-10.65, 7.25]
Horner 2015	3.29	11.75	23	1.13	11.16	16	1.6%	2.16 [-5.12, 9.44]
Jaime 2017	0	16.52	11	-7	11.79	8	0.7%	7.00 [-5.73, 19.73]
Jaime 2019	-1	17.32	12	3	12.96	8	0.7%	-4.00 [-17.29, 9.29]
**Subtotal (95% CI)**			**141**			**116**	**14.4%**	**-0.59 [-3.07, 1.89]**
Heterogeneity: Tau² = 2.76; Chi² = 9.97, df = 8 (P = 0.27); I² = 20%								
Test for overall effect: Z = 0.46 (P = 0.64)								
**1.2.4 CT vs CON**								
Dobrosielski 2012	-0.4	11.71	70	-0.8	10.04	70	3.1%	0.40 [-3.21, 4.01]
Lee 2015	-4.93	12.35	14	-1.83	11.4	12	1.2%	-3.10 [-12.24, 6.04]
Loimaala 2008	-3	12.85	24	-1	12.67	24	1.7%	-2.00 [-9.22, 5.22]
Park 2020	-2.4	2.72	10	1.6	3.35	10	3.5%	-4.00 [-6.67, -1.33]
Son 2016	-13.43	10.99	10	-1.46	9.4	10	1.2%	-11.97 [-20.93, -3.01]
Stewart 2005	-5.3	1.4	51	-4.5	1.13	53	4.3%	-0.80 [-1.29, -0.31]
**Subtotal (95% CI)**			**179**			**179**	**15.0%**	**-2.20 [-4.49, 0.09]**
Heterogeneity: Tau² = 3.64; Chi² = 11.98, df = 5 (P = 0.04); I² = 58%								
Test for overall effect: Z = 1.88 (P = 0.06)								
**1.2.5 WBV vs CON**								
Alvarez 2017	-4	5	25	1	10.82	13	2.0%	-5.00 [-11.20, 1.20]
Figueroa 2012	-5	5.93	5	2	4.47	5	1.9%	-7.00 [-13.51, -0.49]
Figueroa 2013	-12	14.42	13	2	12.51	12	1.0%	-14.00 [-24.56, -3.44]
Figueroa 2014	-11	13.86	12	2	10.39	12	1.1%	-13.00 [-22.80, -3.20]
Yule 2016	3.3	12.88	3	-2	14.79	3	0.3%	5.30 [-16.89, 27.49]
**Subtotal (95% CI)**			**58**			**45**	**6.2%**	**-7.83 [-11.98, -3.68]**
Heterogeneity: Tau² = 2.97; Chi² = 4.58, df = 4 (P = 0.33); I² = 13%								
Test for overall effect: Z = 3.70 (P = 0.0002)								
**1.2.6 ISTA vs CON**								
Ichihara 2005	-3	4	22	-4	8.72	20	2.8%	1.00 [-3.17, 5.17]
Igase 2012	0.3	14.07	26	0	13.85	25	1.5%	0.30 [-7.36, 7.96]
**Subtotal (95% CI)**			**48**			**45**	**4.4%**	**0.84 [-2.82, 4.50]**
Heterogeneity: Tau² = 0.00; Chi² = 0.02, df = 1 (P = 0.88); I² = 0%								
Test for overall effect: Z = 0.45 (P = 0.65)								
**1.2.7 mSTA vs CON**								
Grigoropoulou 2018	-3	16.52	46	-2	16.09	33	1.6%	-1.00 [-8.28, 6.28]
Ichihara 2005	-2	6.24	21	-4	8.72	20	2.6%	2.00 [-2.66, 6.66]
Kanaki 2013	-4	7	25	1	6.5	25	3.0%	-5.00 [-8.74, -1.26]
Oh 2014	-3.6	13.27	41	-1.4	13.62	15	1.5%	-2.20 [-10.20, 5.80]
Pirro 2006	-3	10	35	-2	10.15	36	2.6%	-1.00 [-5.69, 3.69]
Qi 2007	-4.89	9.04	42	-4	10.02	45	2.9%	-0.89 [-4.90, 3.12]
Raison 2002	-3.76	7.79	12	-10.12	8.26	11	1.9%	6.36 [-0.22, 12.94]
**Subtotal (95% CI)**			**222**			**185**	**16.1%**	**-0.59 [-3.23, 2.06]**
Heterogeneity: Tau² = 5.46; Chi² = 10.87, df = 6 (P = 0.09); I² = 45%								
Test for overall effect: Z = 0.43 (P = 0.66)								
**1.2.8 hSTA vs CON**								
Ichihara 2005	-5	16	16	-3	12.65	10	0.9%	-2.00 [-13.09, 9.09]
Orr 2009	-8	11.29	15	-1.7	16	15	1.1%	-6.30 [-16.21, 3.61]
Zhang 2003	-1	5.29	22	-4	8.72	20	2.7%	3.00 [-1.41, 7.41]
**Subtotal (95% CI)**			**53**			**45**	**4.7%**	**-0.23 [-5.96, 5.50]**
Heterogeneity: Tau² = 10.22; Chi² = 3.15, df = 2 (P = 0.21); I² = 37%								
Test for overall effect: Z = 0.08 (P = 0.94)								
**Total (95% CI)**			**1278**			**1160**	**100.0%**	**-3.02 [-4.20, -1.83]**
Heterogeneity: Tau² = 8.45; Chi² = 151.41, df = 54 (P < 0.00001); I² = 64%								
Test for overall effect: Z = 4.99 (P < 0.00001)								
Test for subgroup differences: Chi² = 20.39, df = 7 (P = 0.005), I² = 65.7%								

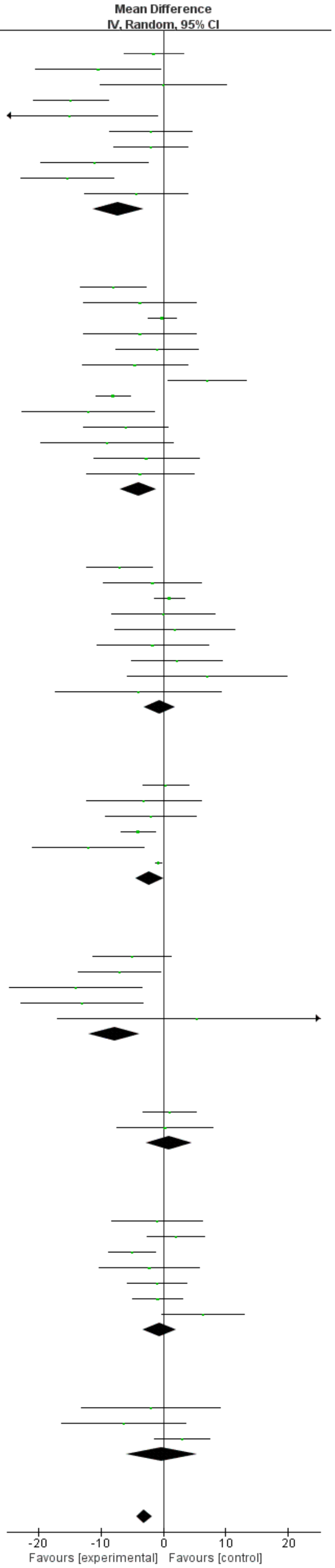

Appendix 7.2 forest plot of Systolic Blood Pressure.

	Experimental			Control				Mean Difference
Study or Subgroup	Mean	SD	Total	Mean	SD	Total	Weight	IV, Random, 95% CI
**1.5.1 INT vs CON**								
Adams 2017	-5.5	7.66	35	1.5	6.72	27	2.6%	-7.00 [-10.59, -3.41]
Aghaei 2019	-9.29	11.5	10	1.43	4.46	10	1.1%	-10.72 [-18.36, -3.08]
Bellia 2017	1	7.41	11	-3	13.35	11	0.9%	4.00 [-5.02, 13.02]
Bouaziz 2013	-6.1	6.85	30	3.2	7.18	30	2.6%	-9.30 [-12.85, -5.75]
Bouaziz 2019	-6.29	18.71	30	3.49	22.27	30	0.7%	-9.78 [-20.19, 0.63]
Deiseroth 2019	-1	8.89	38	-1	10.54	30	2.0%	0.00 [-4.71, 4.71]
Guimara˜es 2010	-2	5.57	16	-1	8	16	2.0%	-1.00 [-5.78, 3.78]
KIM 2017	0.01	8.49	0	-1	7.48	14		Not estimable
Rodriguez 2017	-7	8.72	23	-1	9.85	23	1.8%	-6.00 [-11.38, -0.62]
Taha 2023	-8	3.16	30	-1.32	3.52	30	3.6%	-6.68 [-8.37, -4.99]
Way 2020	0.01	7	0	-4	8	11		Not estimable
Zhang 2022	9	8.5	39	-2.9	10	38	2.3%	11.90 [7.75, 16.05]
**Subtotal (95% CI)**			**262**			**270**	**19.4%**	**-3.35 [-7.60, 0.91]**
Heterogeneity: Tau² = 38.85; Chi² = 87.95, df = 9 (P < 0.00001); I² = 90%								
Test for overall effect: Z = 1.54 (P = 0.12)								
**1.5.2 AE vs CON**								
Beck 2013	-3	6.71	13	-7	7.21	15	1.8%	4.00 [-1.16, 9.16]
Connor 2017	-2.5	13.85	13	2.1	8.55	20	1.0%	-4.60 [-13.01, 3.81]
Davis 2019	1.1	5.49	90	0.01	5.33	85	3.6%	1.09 [-0.51, 2.69]
Greenwood 2014	-2.5	13.85	13	2.1	8.55	20	1.0%	-4.60 [-13.01, 3.81]
Guimara˜es 2010	-1	9	11	-1	8	16	1.4%	0.00 [-6.61, 6.61]
Horner 2015	-2.95	9.51	27	0.08	6.44	16	2.0%	-3.03 [-7.81, 1.75]
Madden 2009	-5	2	18	-1	2	18	3.8%	-4.00 [-5.31, -2.69]
Madden 2013	0.01	13.23	25	1	10.39	27	1.4%	-0.99 [-7.49, 5.51]
Millen 2013	-8	9.17	19	-1	8.54	16	1.6%	-7.00 [-12.87, -1.13]
Nualnim 2012	-4	9.8	24	-1	8.72	19	1.7%	-3.00 [-8.54, 2.54]
Shin 2015	0.2	6.8	29	-0.1	6.3	14	2.3%	0.30 [-3.83, 4.43]
Zhou 2010	-2	7.85	17	-0.7	8.57	18	1.7%	-1.30 [-6.74, 4.14]
**Subtotal (95% CI)**			**299**			**284**	**23.2%**	**-1.64 [-3.69, 0.41]**
Heterogeneity: Tau² = 6.68; Chi² = 33.88, df = 11 (P = 0.0004); I² = 68%								
Test for overall effect: Z = 1.57 (P = 0.12)								
**1.5.3 RT vs CON**								
Beck 2013	-7	7.75	15	-3	6.71	15	1.8%	-4.00 [-9.19, 1.19]
Connor 2017	3	11.7	20	2.1	8.55	20	1.4%	0.90 [-5.45, 7.25]
Croymans 2013	-4.5	2.2	28	-10.5	3.89	8	3.0%	6.00 [3.18, 8.82]
DeVallance 2016	-2	7.2	13	1	14.42	16	1.0%	-3.00 [-11.08, 5.08]
Fernandez 2018	1.94	7.25	6	-3.13	8.34	5	0.8%	5.07 [-4.26, 14.40]
Greenwood 2014	3	11.7	13	2.1	8.55	20	1.2%	0.90 [-6.48, 8.28]
Horner 2015	0.56	6.81	23	0.08	6.44	16	2.2%	0.48 [-3.73, 4.69]
Jaime 2017	0	10	11	-1	4.58	8	1.3%	1.00 [-5.71, 7.71]
Jaime 2019	0.01	10.4	12	-1	5.66	8	1.2%	1.01 [-6.06, 8.08]
**Subtotal (95% CI)**			**141**			**116**	**14.0%**	**1.18 [-1.50, 3.87]**
Heterogeneity: Tau² = 7.50; Chi² = 15.59, df = 8 (P = 0.05); I² = 49%								
Test for overall effect: Z = 0.86 (P = 0.39)								
**1.5.4 CT vs CON**								
Dobrosielski 2012	-1.4	6.69	70	-0.4	5.86	70	3.4%	-1.00 [-3.08, 1.08]
Lee 2015	-2.5	7.08	14	1	10.76	12	1.2%	-3.50 [-10.63, 3.63]
Park 2020	-0.1	3.16	10	1.1	3.67	10	2.9%	-1.20 [-4.20, 1.80]
Son 2016	-10.73	9.37	10	-1.86	10.91	10	0.9%	-8.87 [-17.78, 0.04]
Stewart 2005	-3.7	0.68	51	-1.5	0.68	53	4.1%	-2.20 [-2.46, -1.94]
**Subtotal (95% CI)**			**155**			**155**	**12.4%**	**-2.18 [-2.44, -1.92]**
Heterogeneity: Tau² = 0.00; Chi² = 3.96, df = 4 (P = 0.41); I² = 0%								
Test for overall effect: Z = 16.56 (P < 0.00001)								
**1.5.5 WBV vs CON**								
Alvarez 2017	-1	5	25	1	7.21	13	2.2%	-2.00 [-6.38, 2.38]
Figueroa 2012	-2	4.47	5	2	4.47	5	1.7%	-4.00 [-9.54, 1.54]
Figueroa 2013	-6	9.55	13	1	6.93	12	1.4%	-7.00 [-13.51, -0.49]
Yule 2016	-1.3	9.7	3	-1.3	9.61	3	0.3%	0.00 [-15.45, 15.45]
**Subtotal (95% CI)**			**46**			**33**	**5.6%**	**-3.56 [-6.54, -0.57]**
Heterogeneity: Tau² = 0.00; Chi² = 1.79, df = 3 (P = 0.62); I² = 0%								
Test for overall effect: Z = 2.34 (P = 0.02)								
**1.5.6 ISTA vs CON**								
Ichihara 2005	-2	2	22	3	3.61	20	3.5%	-5.00 [-6.79, -3.21]
Igase 2012	1.5	9.97	26	1.8	7.69	25	1.9%	-0.30 [-5.18, 4.58]
**Subtotal (95% CI)**			**48**			**45**	**5.5%**	**-3.22 [-7.69, 1.25]**
Heterogeneity: Tau² = 7.53; Chi² = 3.15, df = 1 (P = 0.08); I² = 68%								
Test for overall effect: Z = 1.41 (P = 0.16)								
**1.5.7 mSTA vs CON**								
Grigoropoulou 2018	-3	9	46	-1	9.17	33	2.3%	-2.00 [-6.07, 2.07]
Ichihara 2005	1	2.65	21	3	3.61	20	3.5%	-2.00 [-3.95, -0.05]
Kanaki 2013	-2	5.57	25	0.01	6.56	25	2.7%	-2.01 [-5.38, 1.36]
Oh 2014	-3.3	7.91	41	-1.2	9.6	15	1.7%	-2.10 [-7.53, 3.33]
Pirro 2006	-3	14	35	1	10	36	1.6%	-4.00 [-9.67, 1.67]
Qi 2007	-2.8	7.8	42	-3	8.1	45	2.7%	0.20 [-3.14, 3.54]
Raison 2002	-0.1	10.32	12	-3.1	10.91	11	0.9%	3.00 [-5.70, 11.70]
**Subtotal (95% CI)**			**222**			**185**	**15.4%**	**-1.66 [-2.97, -0.35]**
Heterogeneity: Tau² = 0.00; Chi² = 3.16, df = 6 (P = 0.79); I² = 0%								
Test for overall effect: Z = 2.48 (P = 0.01)								
**1.5.8 hSTA vs CON**								
Ichihara 2005	-3.3	7.91	41	-1.2	9.6	15	1.7%	-2.10 [-7.53, 3.33]
Orr 2009	-1	10.6	16	0.01	8.38	10	1.2%	-1.01 [-8.36, 6.34]
Zhang 2003	-2.3	9.18	15	-1.8	7.6	15	1.5%	-0.50 [-6.53, 5.53]
**Subtotal (95% CI)**			**72**			**40**	**4.4%**	**-1.30 [-4.83, 2.24]**
Heterogeneity: Tau² = 0.00; Chi² = 0.16, df = 2 (P = 0.92); I² = 0%								
Test for overall effect: Z = 0.72 (P = 0.47)								
**Total (95% CI)**			**1245**			**1128**	**100.0%**	**-1.83 [-2.77, -0.89]**
Heterogeneity: Tau² = 5.69; Chi² = 200.56, df = 51 (P < 0.00001); I² = 75%								
Test for overall effect: Z = 3.80 (P = 0.0001)								
Test for subgroup differences: Chi² = 8.33, df = 7 (P = 0.30), I² = 16.0%								

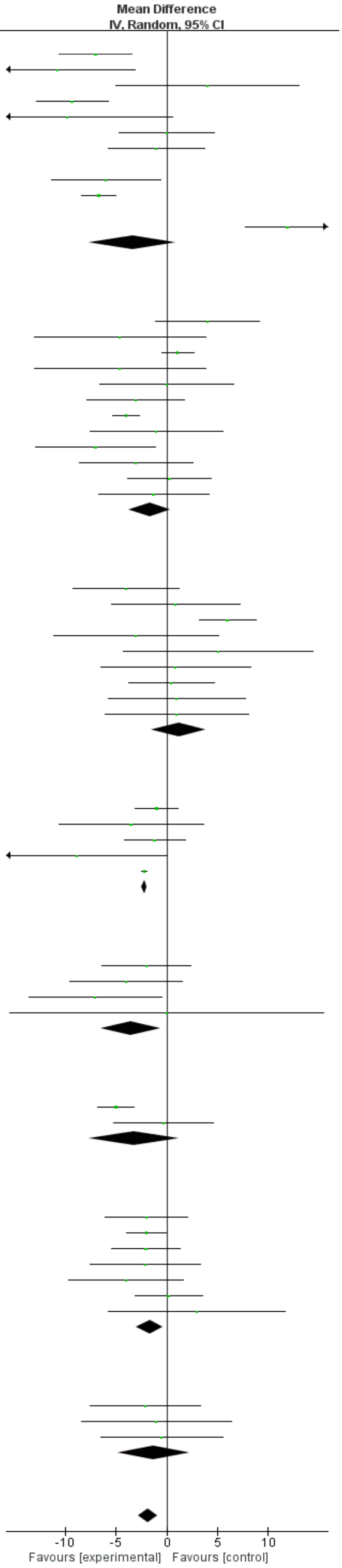

Appendix 7.3 forest plot of Diastolic Blood Pressure.

Appendix 8. Contributions of direct and indirect comparisons to NMA and the number of studies of each direct comparison of Pulse Wave Velocity, Systolic Blood Pressure , and Diastolic Blood Pressure.

Note. A means control group; B means interval training; C means aerobic exercise; D means resistance training; E means combined training; F means whole-body vibration training; G means low doses of statins; H means moderate doses of statins; I means high doses of statins.

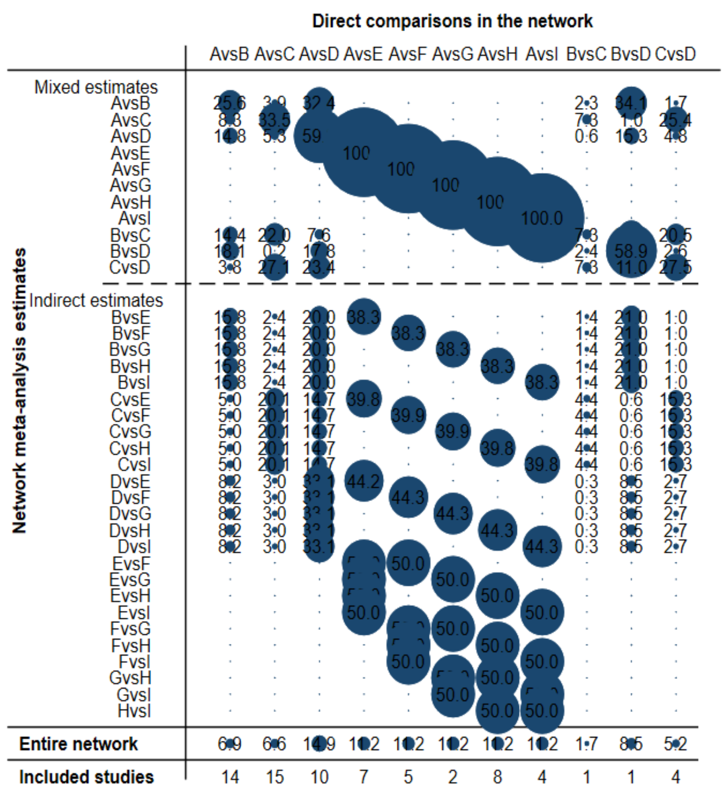

Appendix 8.1 Contribution plot for Pulse Wave Velocity.

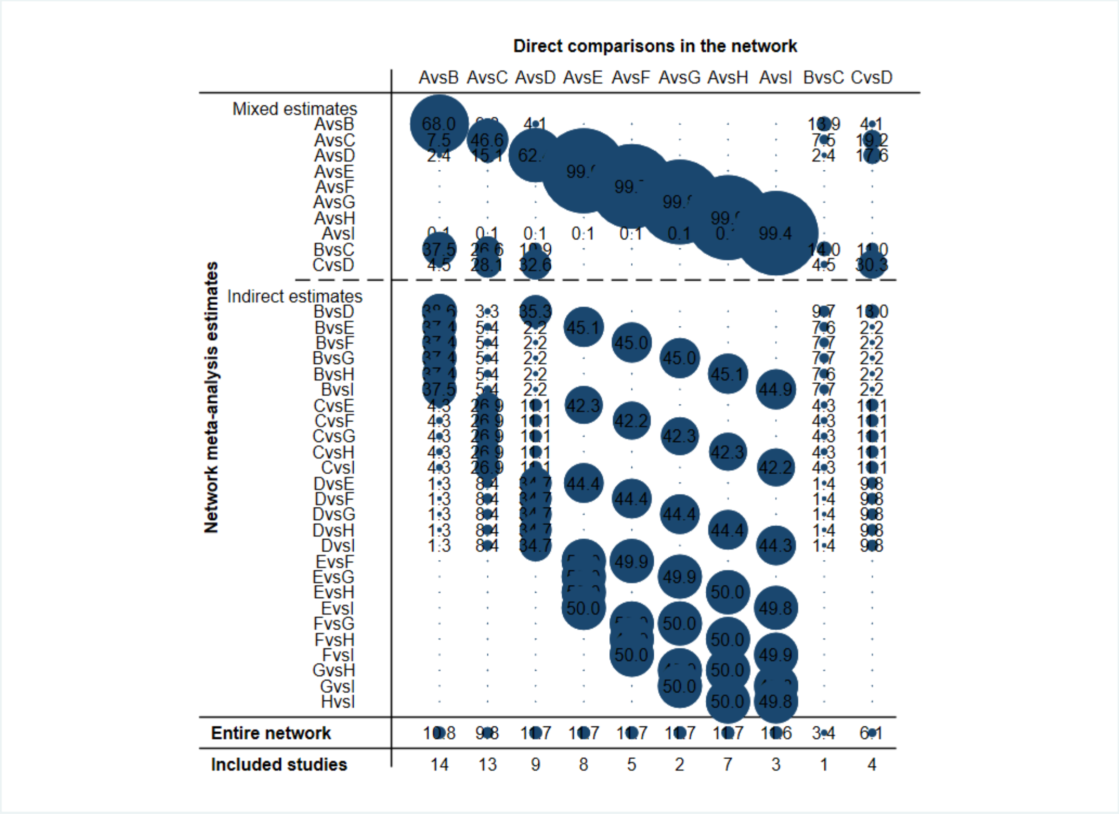

Appendix 8.2 Contribution plot for Systolic Blood Pressure.

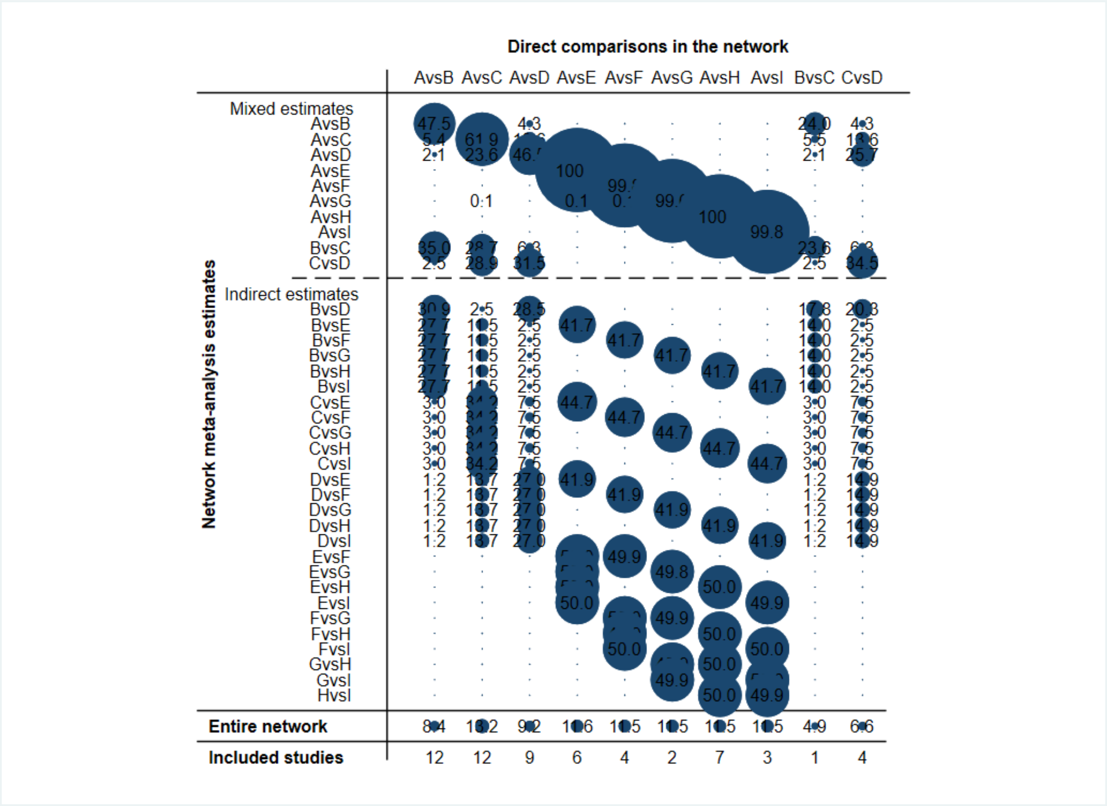

Appendix 8.3 Contribution plot for Diastolic Blood Pressure.

Appendix 9. Inconsistency of Pulse Wave Velocity, Systolic Blood Pressure, and Diastolic Blood Pressure tested by loop-specific heterogeneity estimates, inconsistency model and node splitting analysis.

Note. CON, control group; INT, interval training; AE, means aerobic exercise; RT, resistance training; CT, means combined training.

loop-specific heterogeneity estimates.

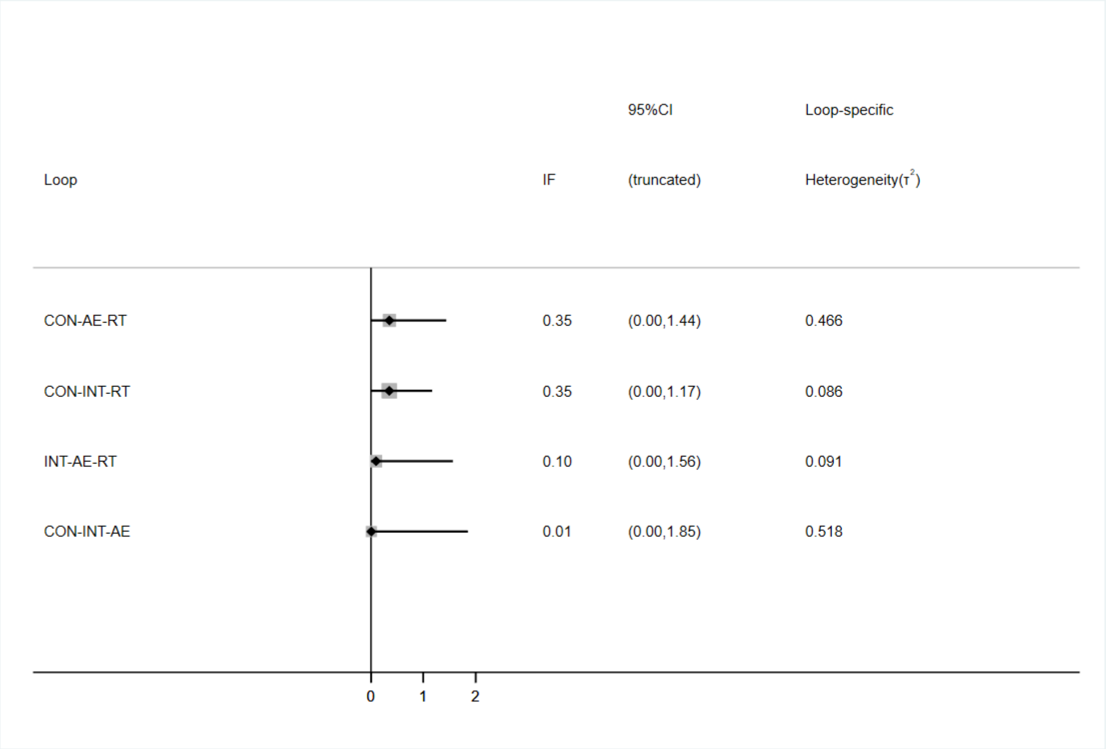

Appendix 9.1 loop-specific heterogeneity estimates for Pulse Wave Velocity.

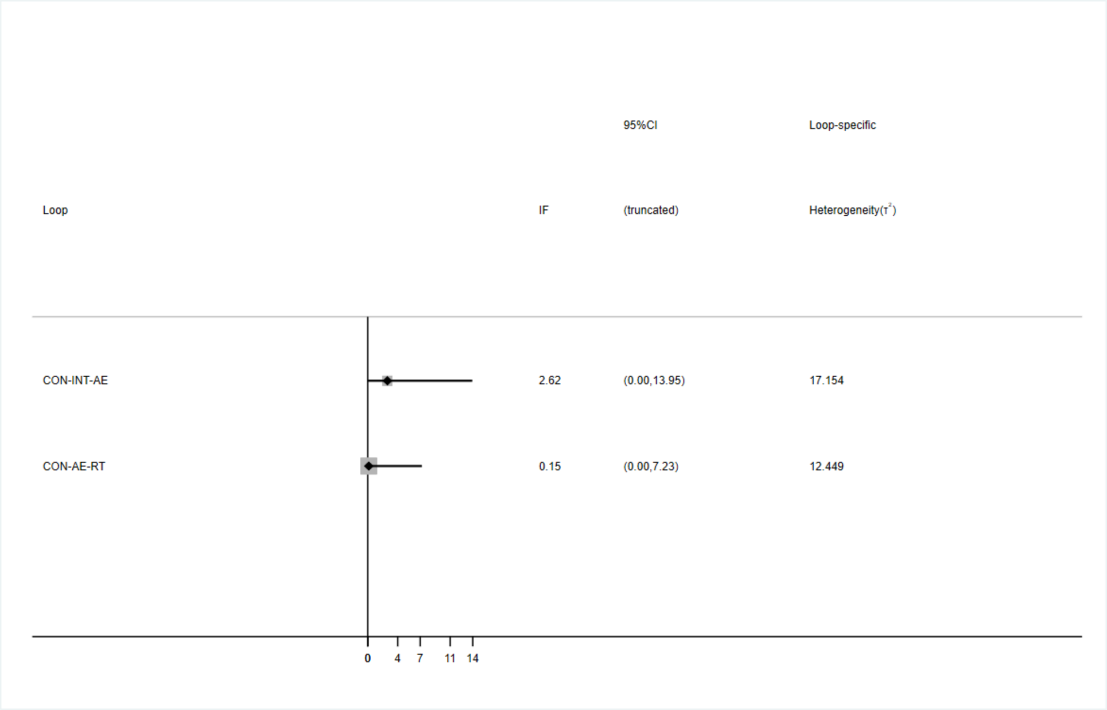

Appendix 9.2 loop-specific heterogeneity estimates for Systolic Blood Pressure.

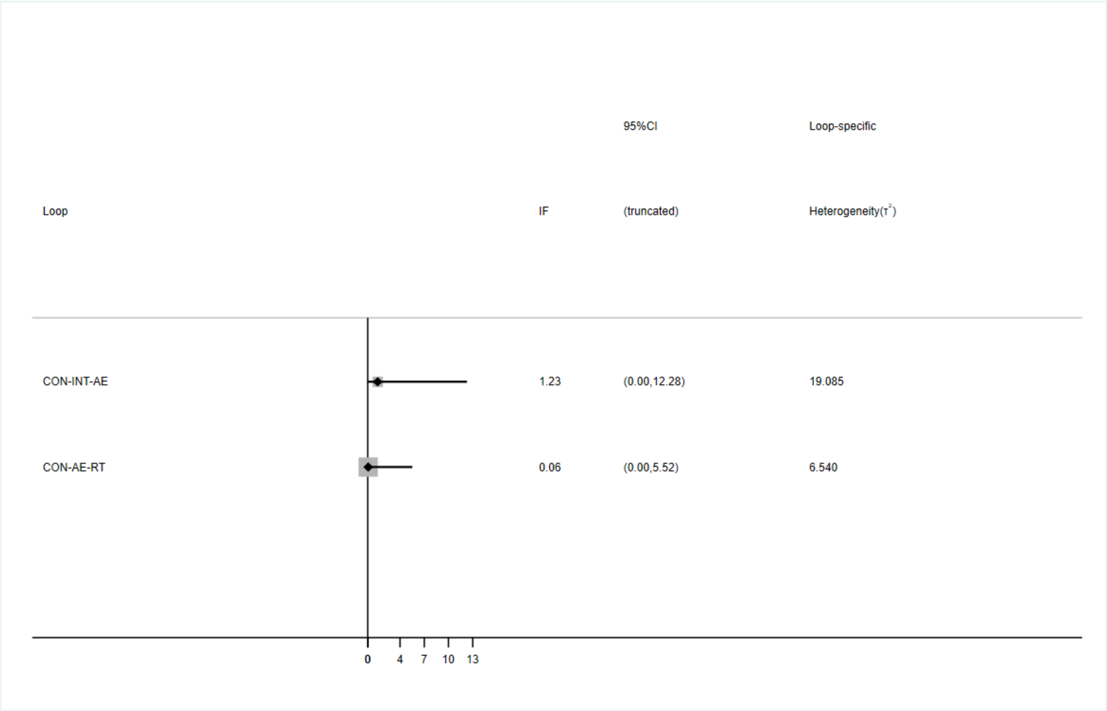

Appendix 9.3 loop-specific heterogeneity estimates for Diastolic Blood Pressure.

Inconsistency model.

Appendix 9.4 Inconsistency model for Pulse Wave Velocity, Systolic Blood Pressure, and Diastolic Blood Pressure.

	Pulse Wave Velocity	Systolic Blood Pressure	Diastolic Blood Pressure
chi2	1.87	2.93	1.53
Prob > chi2	0.9315	0.5695	0.8211

No evidence for the existence of significant global inconsistency

Node splitting analysis.

Appendix 9.5 Node splitting analysis for Pulse Wave Velocity, Systolic Blood Pressure, and Diastolic Blood Pressure.

Side	Direct Coef.	Std. Err.	Indirect Coef.	Std. Err.	Difference Coef.	Std.Err.	P>z
			Pulse Wave Velocity				
A B	-0.772	0.213	-0.667	0.913	-0.105	0.944	0.912
A C	-0.615	0.190	0.094	0.741	-0.709	0.767	0.356
A D	-0.480	0.232	-0.925	0.644	0.445	0.687	0.517
B C	0.120	0.763	0.206	0.285	-0.087	0.815	0.915
B D	0.066	0.590	0.284	0.328	-0.218	0.675	0.747
C D	-0.226	0.375	0.273	0.353	-0.499	0.516	0.334
			Systolic Blood Pressure				
A B	-6.952	1.354	-7.721	8.317	0.768	8.426	0.927
A C	-3.857	1.262	-3.281	4.873	-0.576	4.993	0.908
A D	-0.636	1.613	1.459	5.248	-2.095	5.463	0.701
B C	0.709	4.465	3.599	1.918	-2.890	4.862	0.552
C D	2.812	2.520	3.940	2.595	-1.129	3.632	0.756
			Diastolic Blood Pressure				
A B	-2.464	1.213	-5.223	7.992	2.759	8.124	0.734
A C	-2.326	1.186	0.751	4.486	-3.077	4.608	0.504
A D	1.087	1.487	3.404	4.946	-2.318	5.132	0.652
B C	0.812	4.138	0.316	1.761	0.496	4.499	0.912
C D	3.001	2.357	3.841	2.413	-0.840	3.395	0.805

Appendix 10. Prediction interval forest plots for Pulse Wave Velocity, Systolic Blood Pressure, and Diastolic Blood Pressure.

Note. CON, control group; INT, interval training; AE, means aerobic exercise; RT, resistance training; CT, means combined training; WBV, means whole-body vibration training; lSTA, means low doses of statins; mSTA, means moderate doses of statins; hSTA, means high doses of statins.

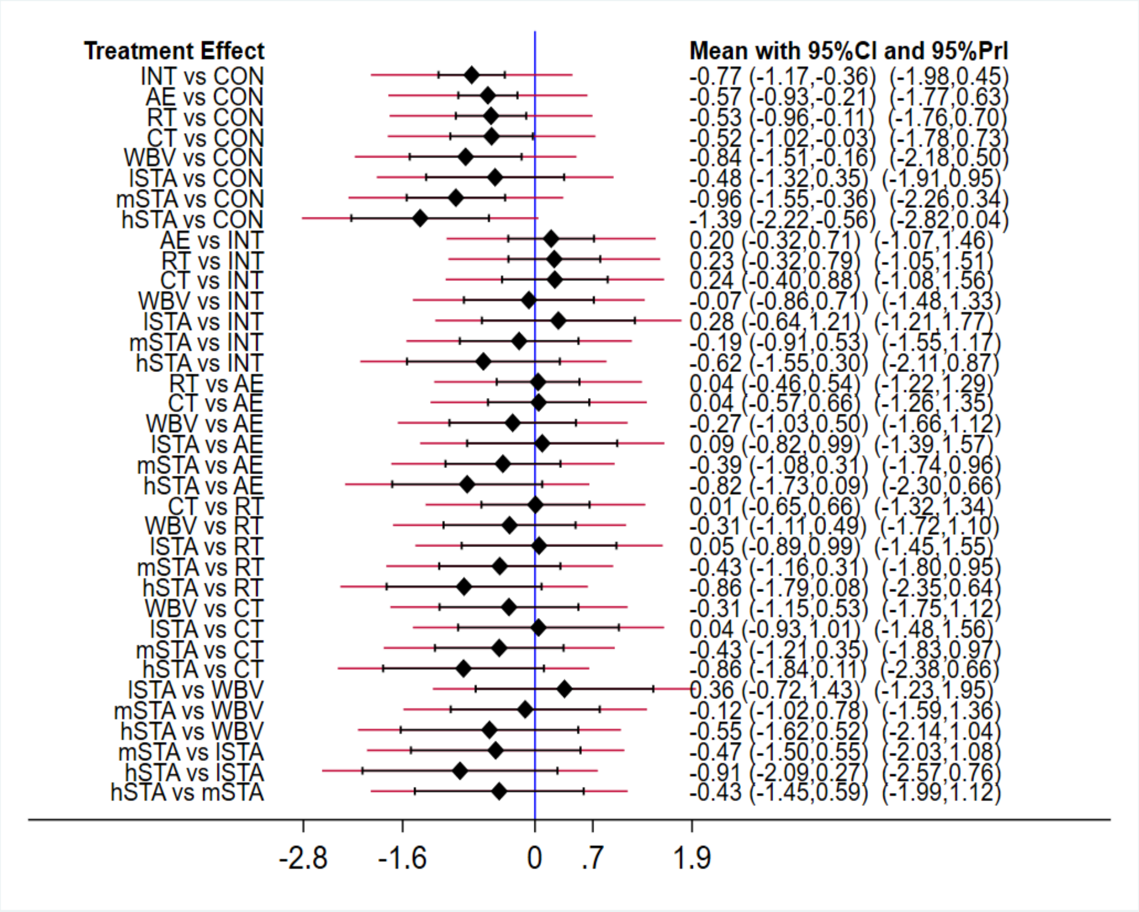

Appendix 10.1 Forest plots of eligible comparisons of Pulse Wave Velocity.

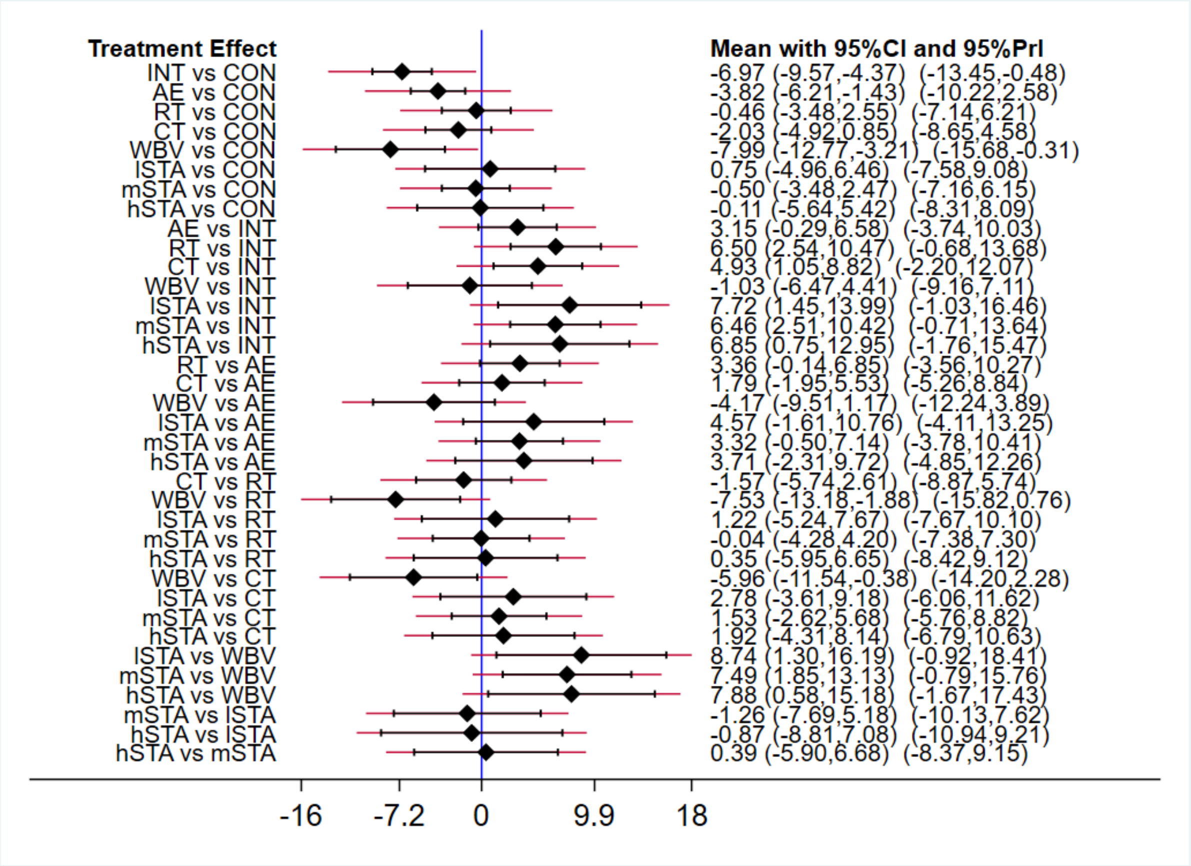

Appendix 10.2 Forest plots of eligible comparisons of Systolic Blood Pressure.

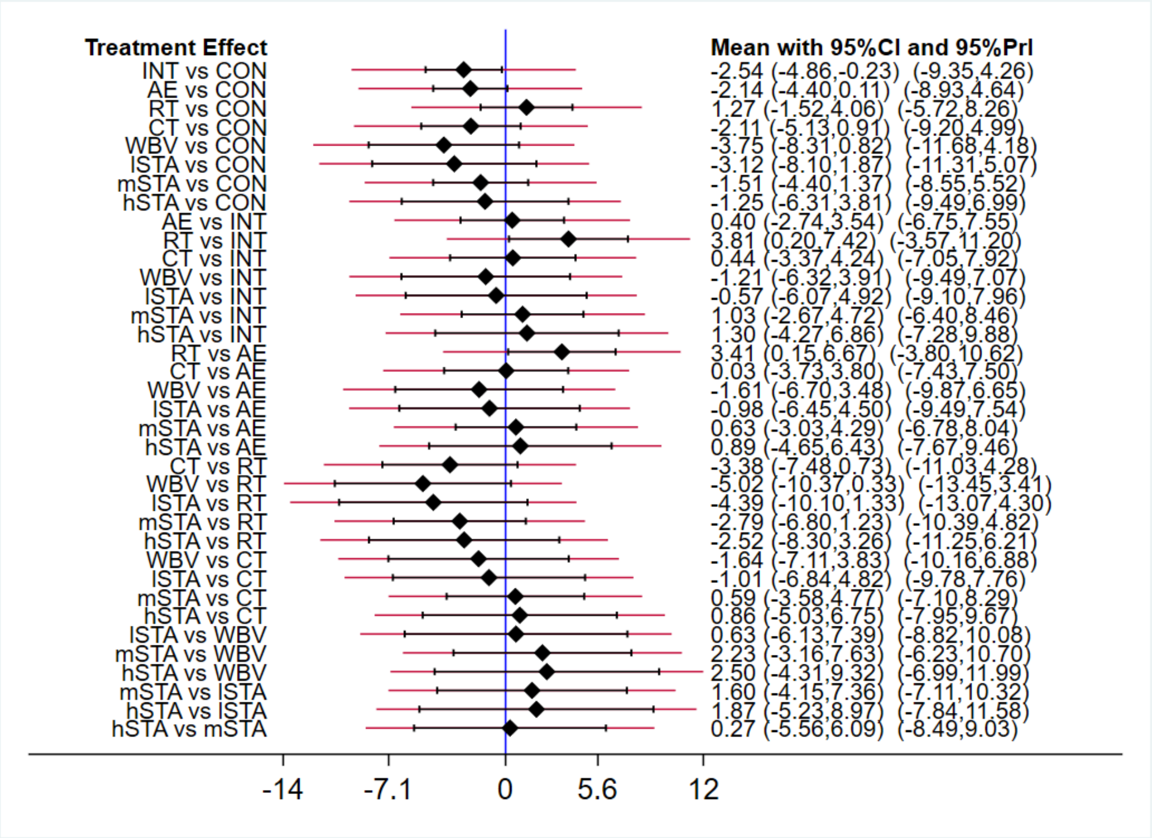

Appendix 10.3 Forest plots of eligible comparisons of Diastolic Blood Pressure.

Appendix 11. The funnel plot graphics of Pulse Wave Velocity, Systolic Blood Pressure, and Diastolic Blood Pressure in NMA.

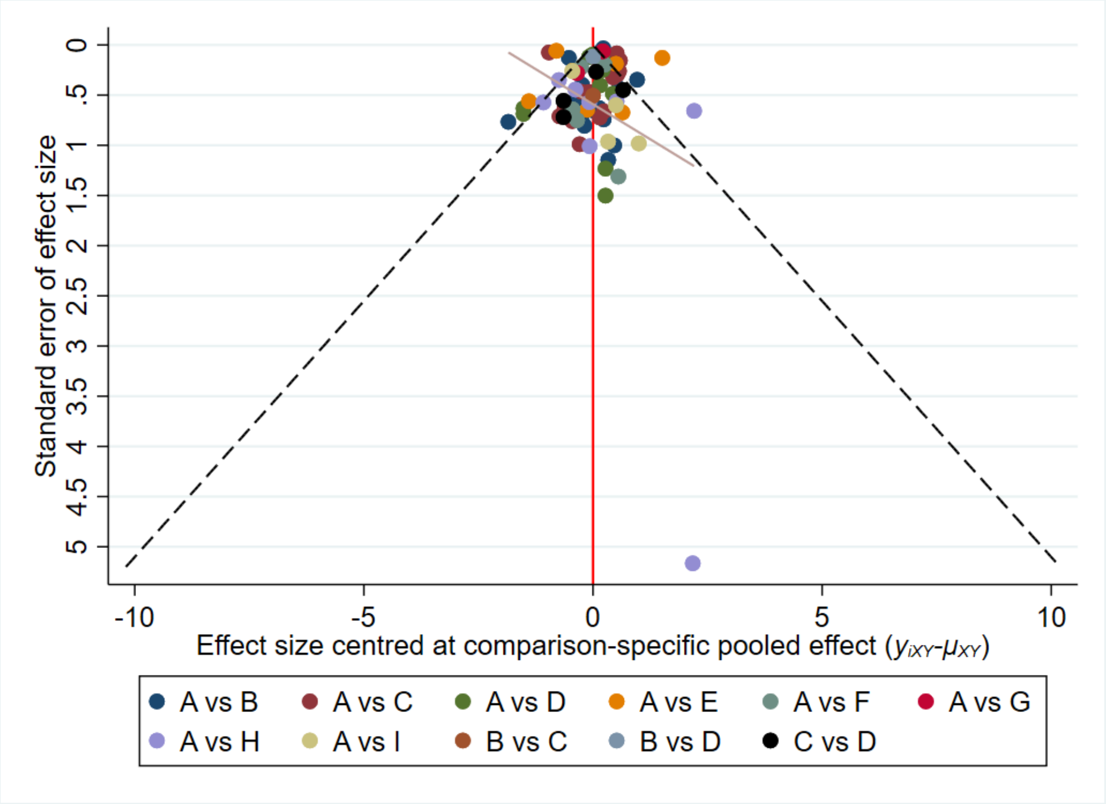

Appendix 11.1 The funnel plot graphics of Pulse Wave Velocity.

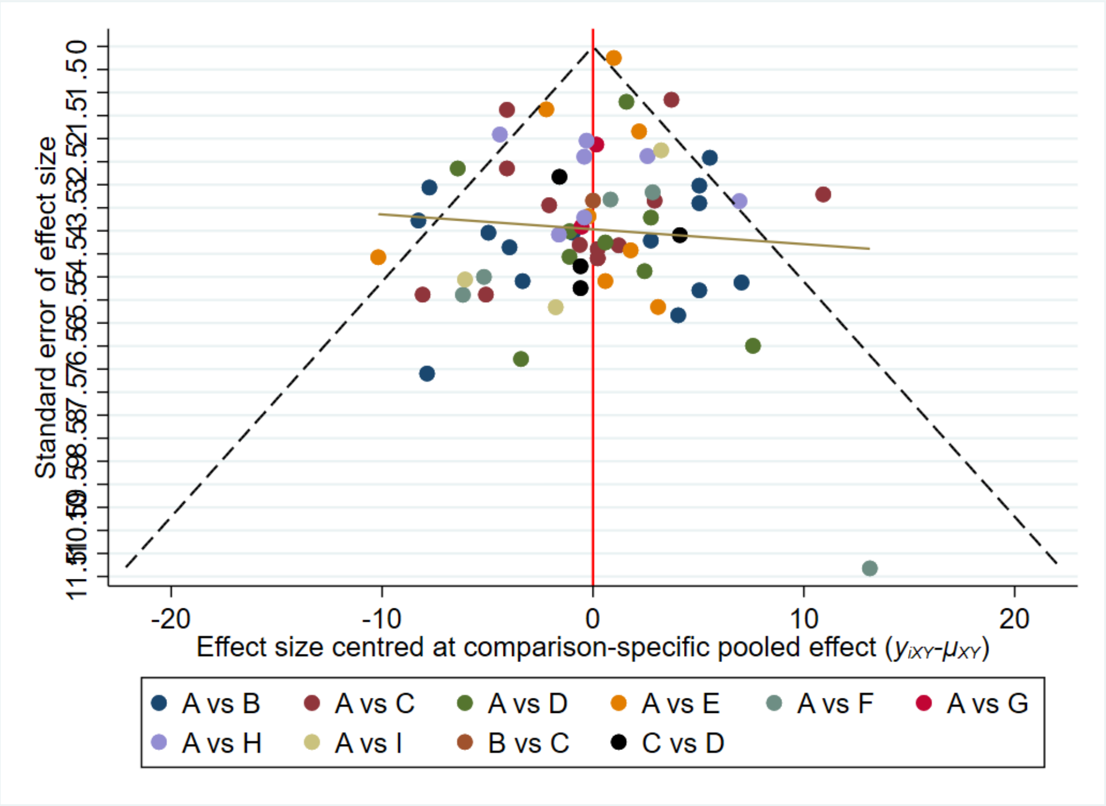

Appendix 11.2 The funnel plot graphics of Systolic Blood Pressure.

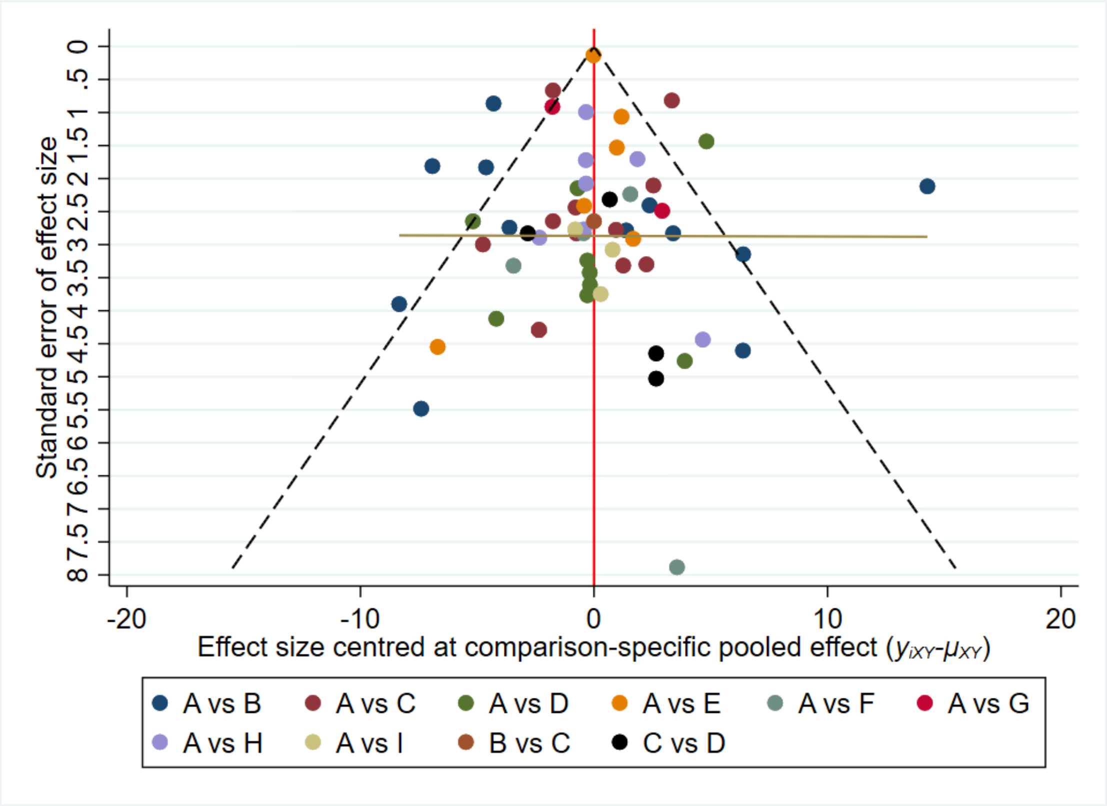

Appendix 11.3 The funnel plot graphics of Diastolic Blood Pressure.

Note. A means control group; B means interval training; C means aerobic exercise; D means resistance training; E means combined training; F means whole-body vibration training; G means low doses of statins; H means moderate doses of statins; I means high doses of statins.

Appendix 12. Area under the curve for cumulative ranking probability of each intervention on Pulse Wave Velocity, Systolic Blood Pressure, and Diastolic Blood Pressure.

Note. CON, control group; INT, interval training; AE, means aerobic exercise; RT, resistance training; CT, means combined training; WBV, means whole-body vibration training; lSTA, means low doses of statins; mSTA, means moderate doses of statins; hSTA, means high doses of statins.

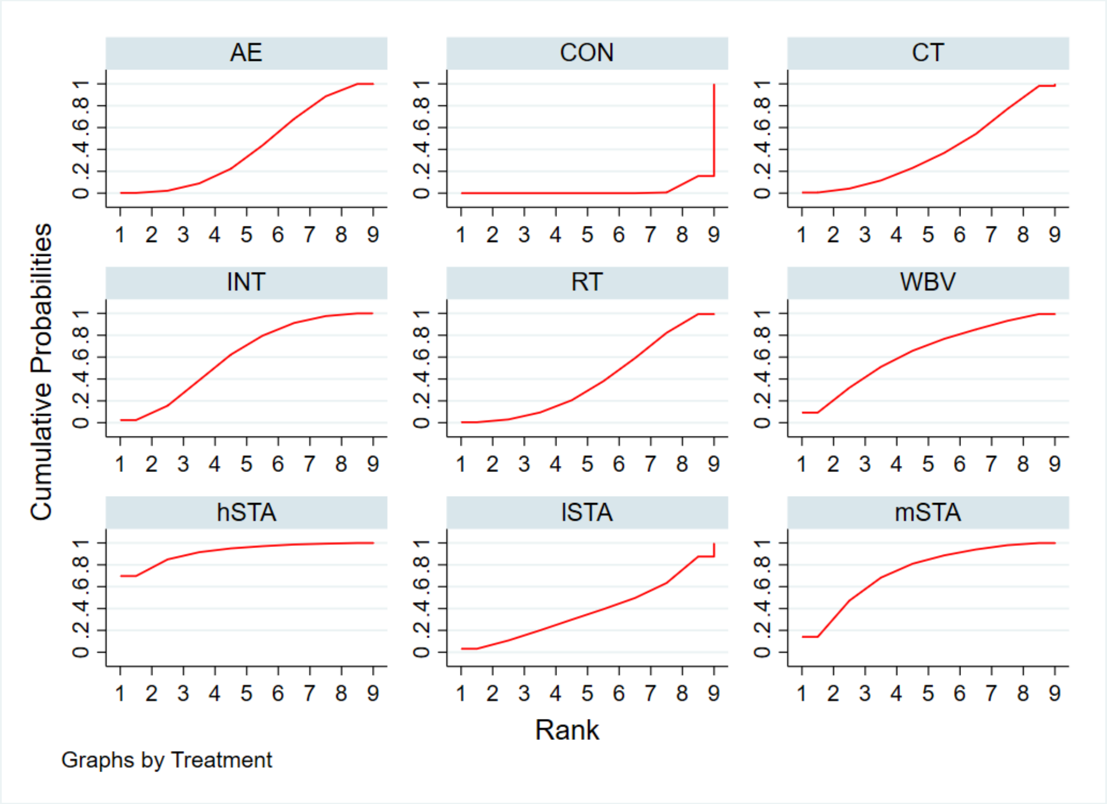

Appendix 12.1 The cumulative ranking probability plot of each intervention on Pulse Wave Velocity.

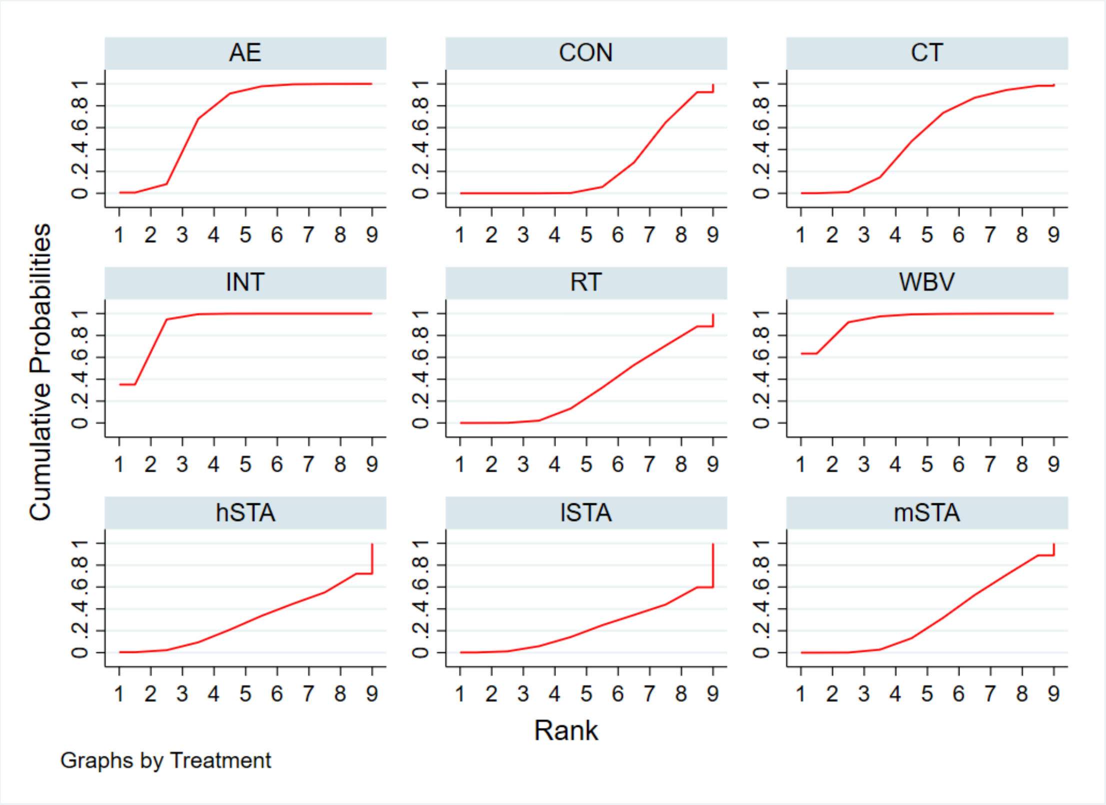

Appendix 12.2 The cumulative ranking probability plot of each intervention on Systolic Blood Pressure.

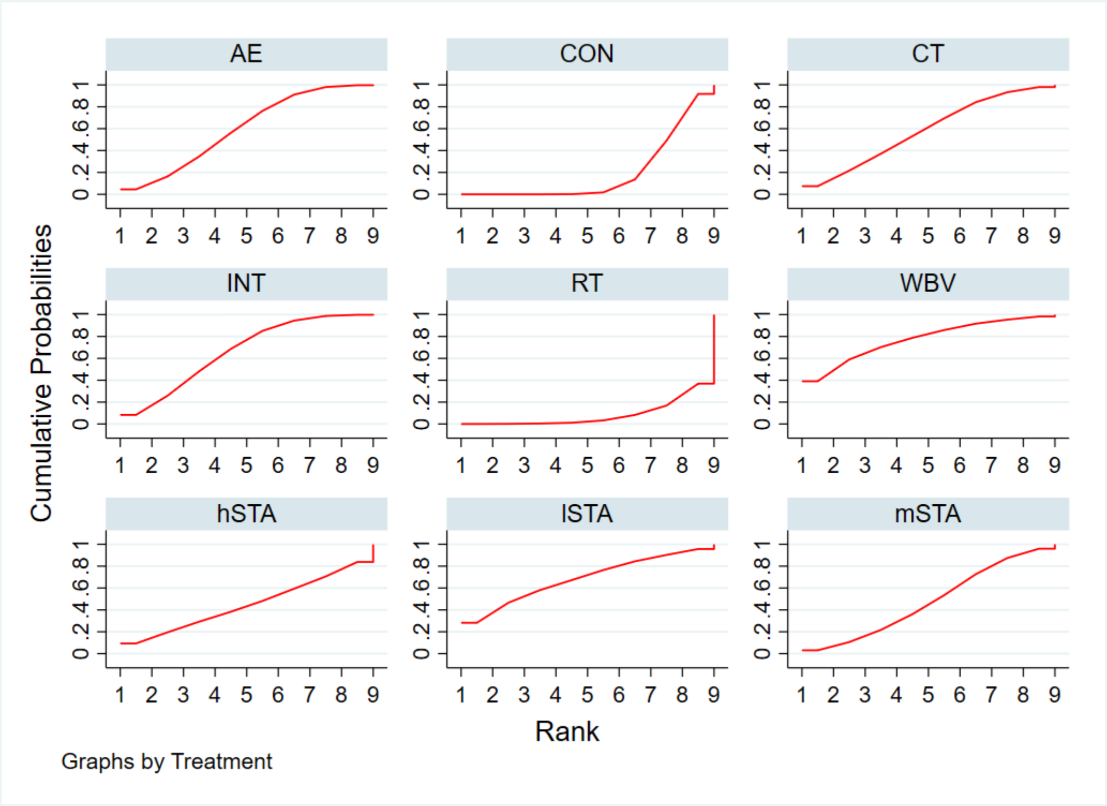

Appendix 12.3 The cumulative ranking probability plot of each intervention on Diastolic Blood Pressure.

Appendix 12.4 Ranking of exercise interventions in order of effectiveness.

PWV (65 studies, N=2931)			
**Treatment**	SUCRA	PrBest (%)	Mean Rank
**hSTA**	92	69.7	1.6
**mSTA**	73.9	14.1	3.1
**WBV**	64.1	9.3	3.9
**INT**	60.9	2.4	4.1
**AE**	41.8	0.2	5.7
**RT**	39	0.5	5.9
**CT**	38.2	0.6	5.9
**lSTA**	38	3.2	6
**CON**	2	0	8.8
**SBP (55 studies, N=2438)**			
**Treatment**	SUCRA	PrBest (%)	Mean Rank
**WBV**	94	63.4	1.5
**INT**	91.2	35.1	1.7
**AE**	70.7	0.7	3.3
**CT**	52.1	0.1	4.8
**mSTA**	32.6	0	6.4
**RT**	32.5	0	6.4
**hSTA**	29.9	0.4	6.6
**CON**	23.9	0	7.1
**lSTA**	23.1	0.2	7.2
**DBP (54studies, N=2373)**			
**Treatment**	SUCRA	PrBest (%)	Mean Rank
**WBV**	77.3	39.1	2.8
**lSTA**	68.4	28.2	3.5
**INT**	66.2	8.3	3.7
**AE**	59.6	4.5	4.2
**CT**	58.1	7.4	4.4
**mSTA**	47.7	3.1	5.2
**hSTA**	44.8	9.4	5.4
**CON**	19.6	0	7.4
**RT**	8.4	0	8.3

Appendix 13. Network of eligible comparisons for Pulse Wave Velocity, Systolic Blood Pressure, and Diastolic Blood Pressure.

Note. CON, control group; INT, interval training; AE, means aerobic exercise; RT, resistance training; CT, combined training; WBV, whole-body vibration training; lSTA, low doses of statins; mSTA, moderate doses of statins; hSTA, high doses of statins.

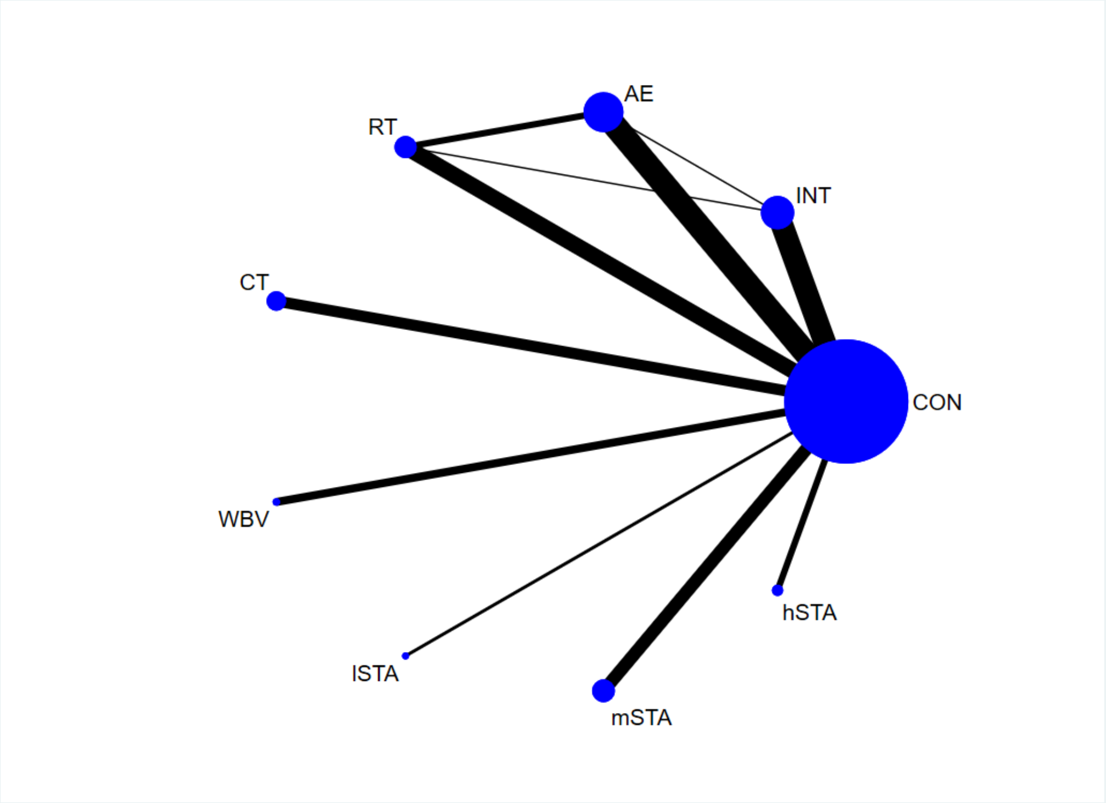

Appendix 13.1 Network of eligible comparisons for Pulse Wave Velocity.

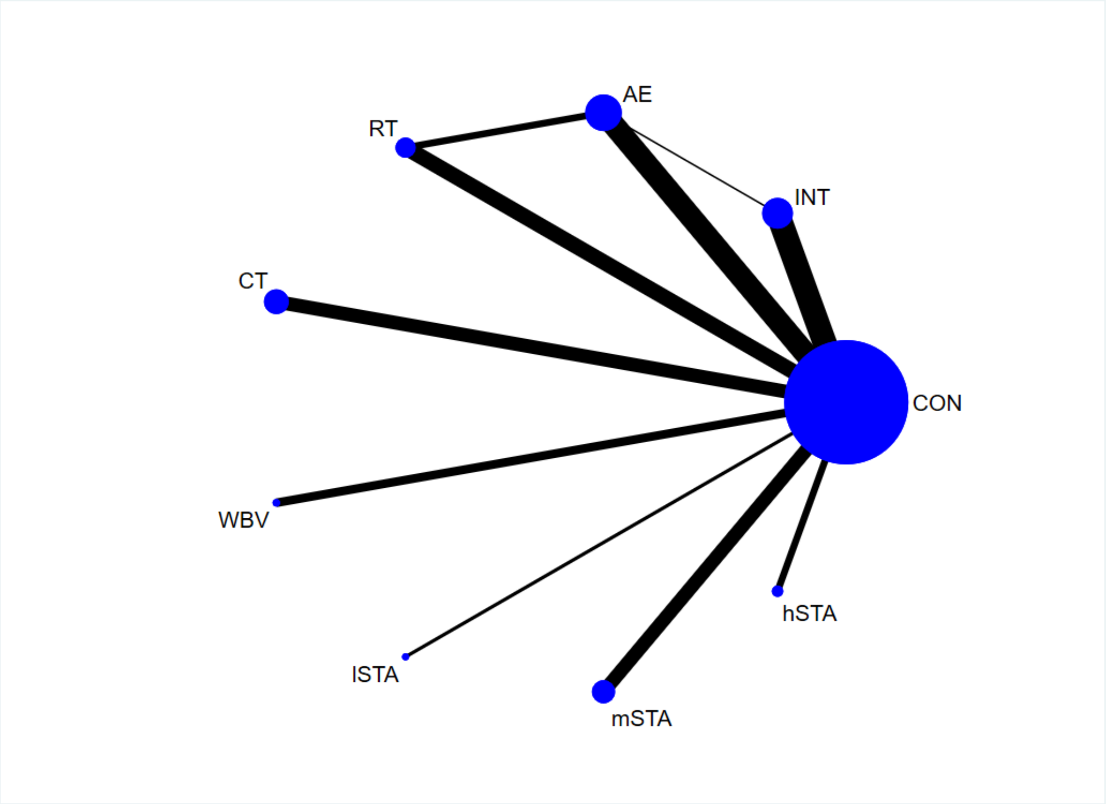

Appendix 13.2 Network of eligible comparisons for Systolic Blood Pressure.

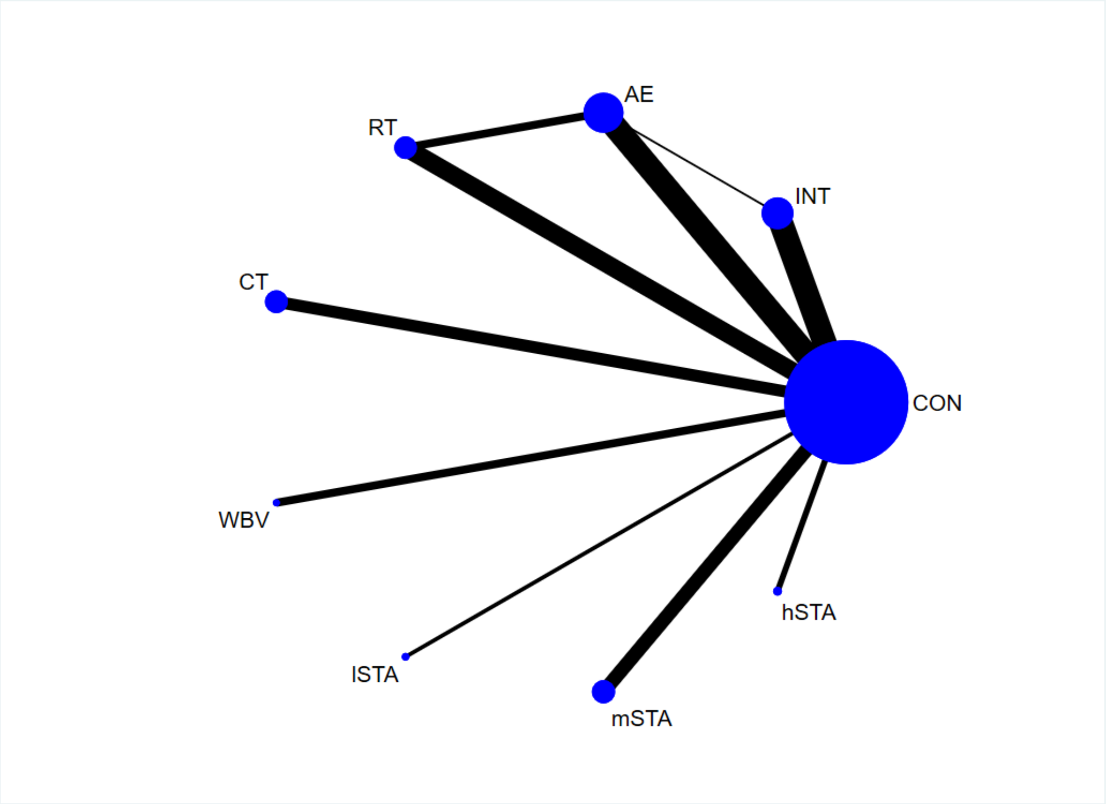

Appendix 13.3 Network of eligible comparisons for Diastolic Blood Pressure.

Appendix 14. GRADE for Pulse Wave Velocity, Systolic Blood Pressure, and Diastolic Blood Pressure.

Appendix 14.1 Summary of study limitations of the included studies.

The colors of the line then indicate the summative RoB assessment of each comparison based on the above information – low RoB comparison [green] and moderate RoB comparison [yellow]. There was no comparison which was judged to be at high RoB.

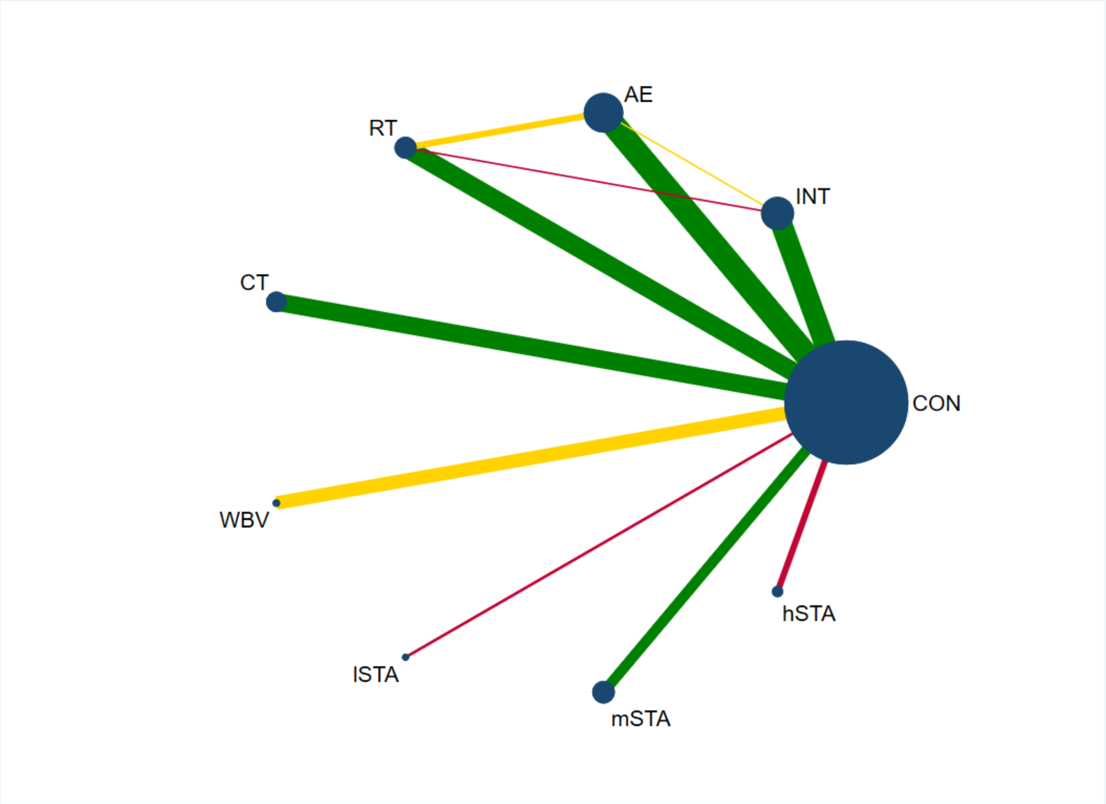

Appendix 14.1.1 Network of literature quality comparisons for Pulse Wave Velocity

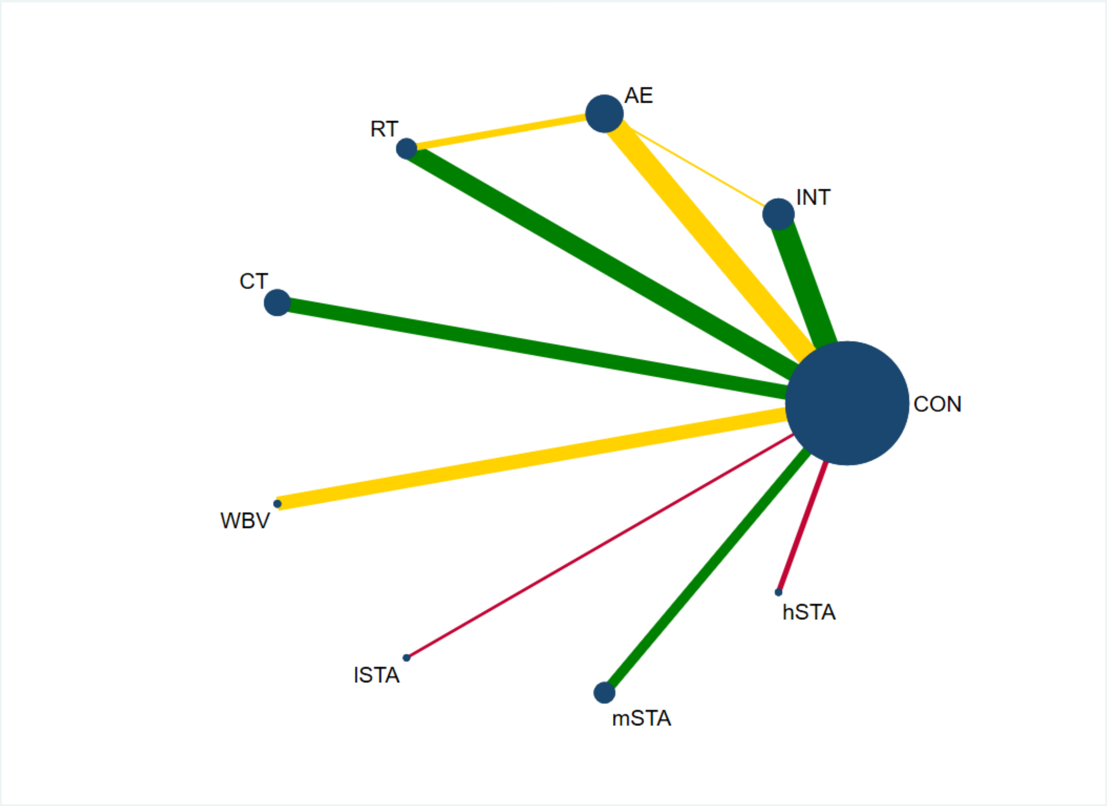

Appendix 14.1.2 Network of literature quality comparisons for Systolic Blood Pressure

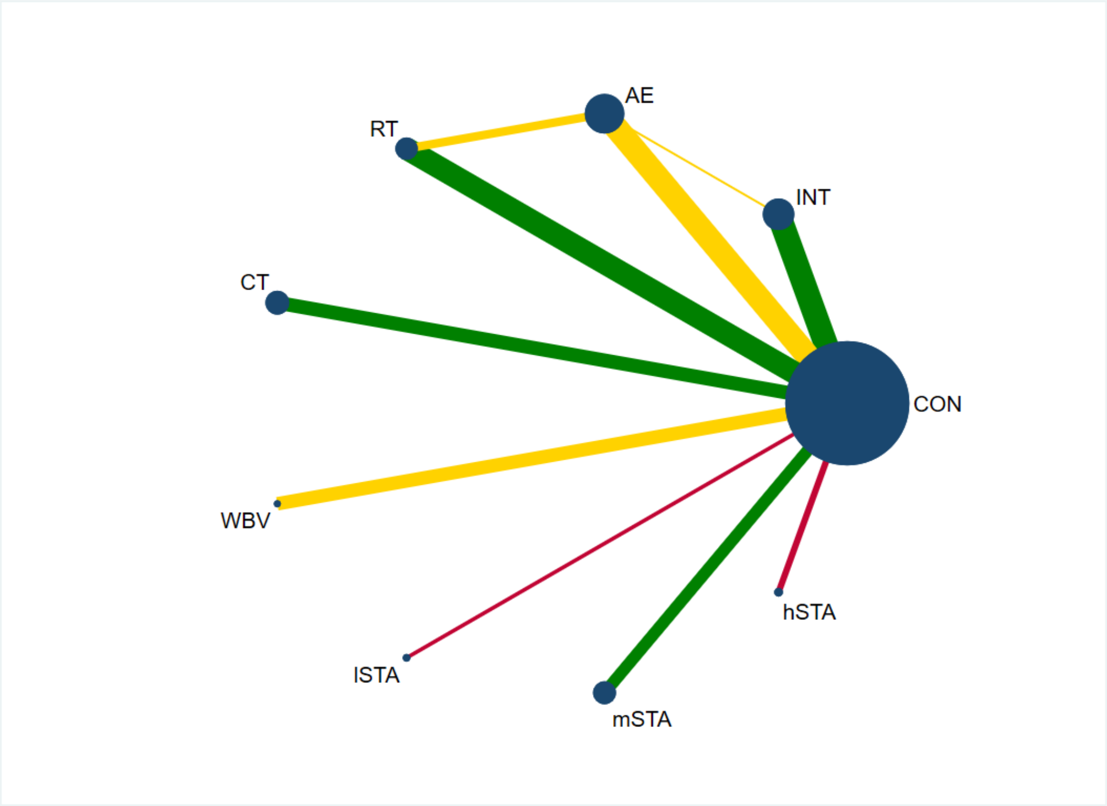

Appendix 14.1.3 Network of literature quality comparisons for Diastolic Blood Pressure

Appendix 14.2 Contribution of ROB comparisons to each network estimate.

Based on the above assessment of RoB for each comparison and the contribution matrix detailing contribution of each direct comparison to all network estimates, the following bar graphs show the percentage of low or moderate RoB contributions for each network estimate.

The judgements about study limitations in each direct comparison is shown at the beginning of the graph.

Each bar corresponds to a NMA relative treatment effect and shows how much information comes from comparisons at low risk of bias [green] or moderate risk of bias [yellow].

Note. A means control group; B means interval training; C means aerobic exercise; D means resistance training; E means combined training; F means whole-body vibration training; G means low doses of statins; H means moderate doses of statins; I means high doses of statins.

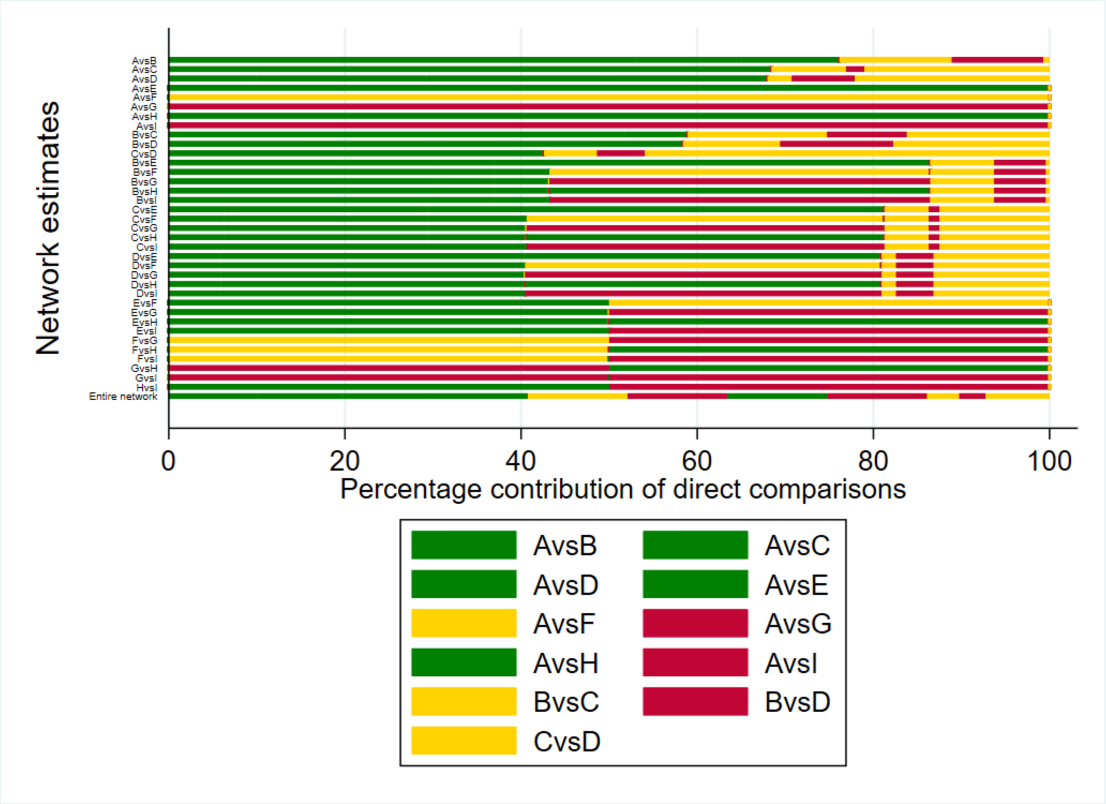

Appendix 14.2.1 Contribution of ROB comparisons to each network estimate for Pulse Wave Velocity.

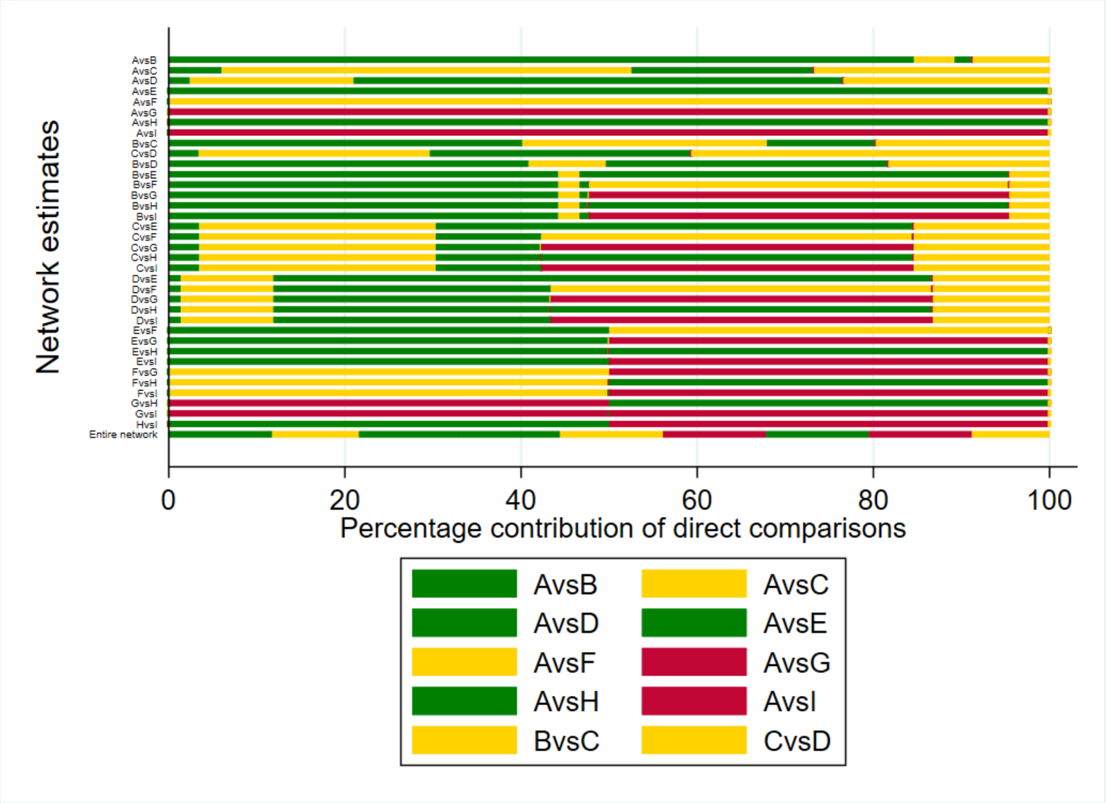

Appendix 14.2.2 Contribution of ROB comparisons to each network estimate for Systolic Blood Pressure

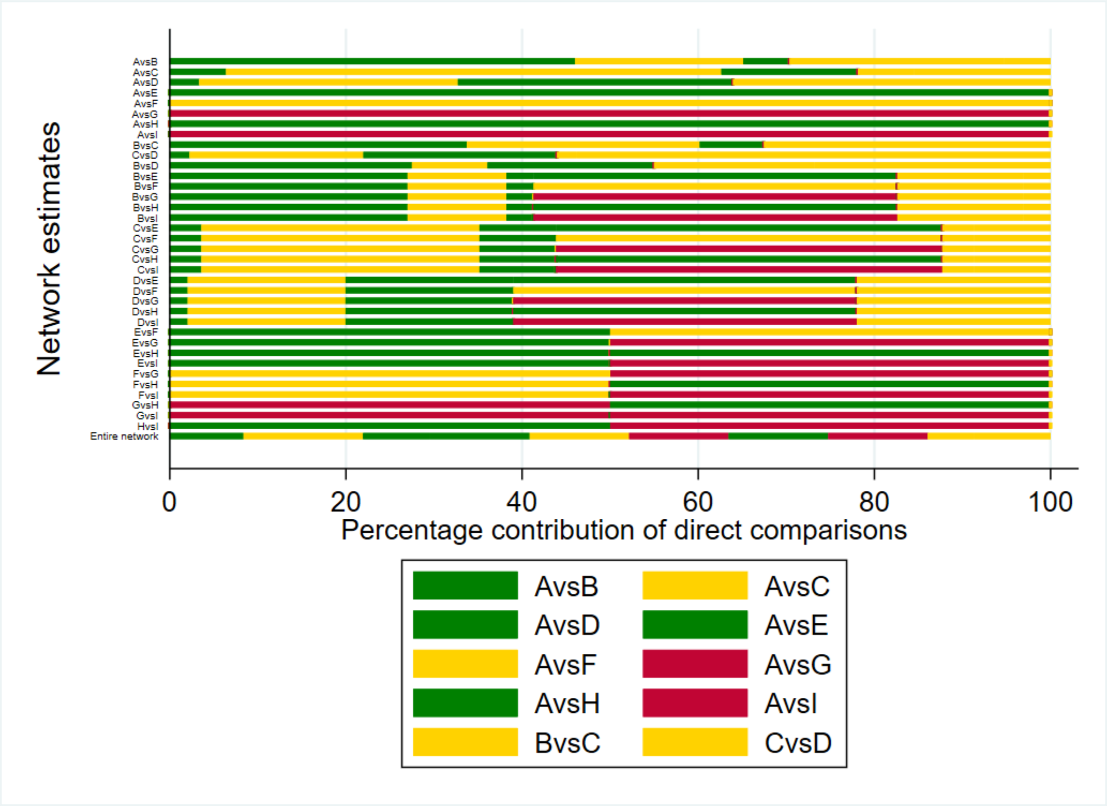

Appendix 14.2.2 Contribution of ROB comparisons to each network estimate for Diastolic Blood Pressure

Appendix 14.3 Table of reasons for downgrading.

Based on all the above information, we GRADE each network estimate according to the following criteria.

(1) Study limitations

Step 1, we summatively rated the overall RoB of each study as follow(low/moderate/high):

Studies were classified as having low risk of bias if none of the domains above was rated as high risk of bias and three or less were rated as unclear risk; moderate if one was as rated as high risk of bias or none was rated as high risk of bias but four or more were rated as unclear risk , and all other cases were assumed to pertain to high risk of bias.(Cipriani 2018)

Step 2, Different weights of 0/-1/-2 are assigned to low/moderate/high, respectively, and the grade of each mixed comparison (NMA) is calculated based on the evidence contribution plot, such as NMA grade CD = AB* weight1 + AC * weight2+ AD * weight3+ BC* weight4+ BD* weight5 + CD * weight6

Step 3, Downgrade according to the calculation results of step 2, less than -1 downgraded by one level, less than -2 downgraded by two levels. (Salanti2014)

(2) Indirectness

First, we judged whether the results of each subgroup in the subgroup analysis were consistent, and the inconsistency should be downgraded by one level, and the consistency should not be downgraded (No subgroups No demote required); Secondly, the results in the contribution graph only comes from indirect comparisons and needs to be downgraded by one level; Third, sorting does not consider downgrade.

(3) Inconsistency

Heterogeneity was judged by looking at the prediction interval plot , and downgraded by one level when there was a significant difference between the fixed-effect model and the random-effects model , and no downgrade when there was no significant difference； Inconsistencies are judged by the p-value of Loop specific and Node

splitting , which need to be downgraded one level when the p-value < 0.05 , and not by a level when the p-value > 0.05； The consistency of the sorting results is judged by the p-value of Global inconsistency, which needs to be downgraded one level when the p-value < 0.05, and not by a level when the p-value > 0.05.

(4) Imprecision

First, imprecision is assessed by sample size, which is downgraded by one level when the sample size < 400, and not downgraded when the sample size > 400. Second, assess the imprecision of ranking results based on the gap between the ranking values. If the gap between the rank values for each treatment is small, indicating poor stability, it is downgraded by one level (Salanti et al ,2014)

(5) Publication bias

Publication bias is judged by observing the comparison-adjusted funnel plot, which need to be downgraded by one level when the number of files on both sides of the vertical line is unevenly distributed.

Appendix 14.3.1 Summary of our confidence in effect estimates and ranking of treatments.

Comparison	Nature of the evidence	Confidence	Downgrading due to
		**Pulse Wave Velocity**	
AB:	Mixed	Moderate	Inconsistency[3]
AC:	Mixed	Moderate	Inconsistency[3]
AD:	Mixed	Moderate	Inconsistency[3]
AE:	Mixed	Moderate	Inconsistency[3]
AF:	Mixed	Very Low	Inconsistency[3]; Study limitations[1]; Inconsistency[3]
AG:	Mixed	Low	Study limitations[1]; Inconsistency[3;]
AH:	Mixed	Low	Inconsistency[3]; Publication bias[5]
AI:	Mixed	Low	Inconsistency[3]; Publication bias[5]
BC:	Mixed	Moderate	Publication bias[5]
BD:	Mixed	High	
CD:	Mixed	Low	Imprecision[4]; Publication bias[5];

BE:	Indirect	Low	Indirectness[2]
BF:	Indirect	Low	Indirectness[2]
BG:	Indirect	Low	Indirectness[2]
BH:	Indirect	Low	Indirectness[2]
BI:	Indirect	Low	Indirectness[2]
CE:	Indirect	Low	Indirectness[2]
CF:	Indirect	Low	Indirectness[2]
CG:	Indirect	Low	Indirectness[2]
CH:	Indirect	Low	Indirectness[2]
CI:	Indirect	Low	Indirectness[2]
DE:	Indirect	Low	Indirectness[2]; Imprecision[4]
DF:	Indirect	Moderate	Indirectness[2]
DG:	Indirect	Low	Indirectness[2]; Imprecision[4]
DH:	Indirect	Low	Indirectness[2]
DI:	Indirect	Low	Indirectness[2]
EF:	Indirect	Low	Indirectness[2]; Imprecision[4]
EG:	Indirect	Very Low	Study limitations[1]; Indirectness[2]; Imprecision[4]
EH:	Indirect	Low	Indirectness[2]
EI:	Indirect	Low	Indirectness[2]
EI:	Indirect	Low	Indirectness[2]
FG:	Indirect	Very Low	Indirectness[2]; Study limitations[1]; Indirectness[2]
FH:	Indirect	Low	Indirectness[2]
FI:	Indirect	Low	Indirectness[2]
GH:	Indirect	Low	Study limitations[1]; Indirectness[2]
GI:	Indirect	Low	Study limitations[1]; Indirectness[2]
HI:	Indirect	Moderate	Indirectness[2]
Ranking of treatments		Moderate	Imprecision[4]
**Systolic Blood Pressure**			
AB:	Mixed	Very Low	Indirectness[2]; Publication bias[5]; Publication bias[5]
AC:	Mixed	Low	Indirectness[2]; Inconsistency[3];
AD:	Mixed	Low	Indirectness[2]; Publication bias[5]
AE:	Mixed	Moderate	Indirectness[2]
AF:	Mixed	Low	Study limitations[1]; Indirectness[2]
AG:	Mixed	Low	Study limitations[1]; Indirectness[2] Imprecision[4]
AH:	Mixed	Low	Study limitations[1]; Publication bias[5]
AI:	Mixed	Moderate	Indirectness[2]
BC:	Mixed	Low	Indirectness[2]; Inconsistency[6]

CD:	Mixed	Low	Indirectness[2]; Inconsistency[6]
BD:	Indirect	Low	Indirectness[2]; Inconsistency[3]
BE:	Indirect	Low	Indirectness[2]; Inconsistency[3]
BF:	Indirect	Low	Indirectness[2]; Inconsistency[3]
BG:	Indirect	Very Low	Study limitations[1]; Indirectness[2]; Inconsistency[3]
BH:	Indirect	Low	Indirectness[2]; Inconsistency[3]
BI:	Indirect	Low	Indirectness[2]; Inconsistency[3]
CE:	Indirect	Moderate	Indirectness[2]
CF:	Indirect	Moderate	Indirectness[2]
CG:	Indirect	Moderate	Study limitations[1]
CH:	Indirect	Moderate	Indirectness[2]
CI:	Indirect	Moderate	Indirectness[2]
DE:	Indirect	Moderate	Indirectness[2]
DF:	Indirect	Low	Indirectness[2]; Inconsistency[3]
DG:	Indirect	Low	Study limitations[1]; Indirectness[2]
DH:	Indirect	Low	Indirectness[2]; Imprecision[4]
DI:	Indirect	Low	Indirectness[2]; Imprecision[4]
EF:	Indirect	Low	Indirectness[2]; Inconsistency[3]
EG:	Indirect	Low	Study limitations[1]; Indirectness[2]
EH:	Indirect	Moderate	Indirectness[2]
EI:	Indirect	Moderate	Indirectness[2]
EI:	Indirect	Moderate	Indirectness[2]
FG:	Indirect	Low	Study limitations[1]; Indirectness[2]; Inconsistency[3]
FH:	Indirect	Low	Indirectness[2]; Inconsistency[3]
FI:	Indirect	Low	Indirectness[2]; Inconsistency[3]
GH:	Indirect	Low	Indirectness[2]; Study limitations[1]
GI:	Indirect	Moderate	Indirectness[2]
HI:	Indirect	Low	Indirectness[2]; Imprecision[4]
Ranking of treatments		Moderate	Imprecision[4]
**Diastolic Blood Pressure**			
AB:	Mixed	Moderate	Inconsistency[3]
AB:	Mixed	High	
AC:	Mixed	High	
AD:	Mixed	Low	Publication bias[5]
AE:	Mixed	High	
AF:	Mixed	Moderate	Study limitations[1]
AG:	Mixed	Moderate	Study limitations[1]
AH:	Mixed	Moderate	Publication bias[5]
AI:	Mixed	High	

BC:	Mixed	High	
CD:	Mixed	Moderate	Inconsistency[3]
BD:	Indirect	Low	Indirectness[2]; Inconsistency[3]
BE:	Indirect	Moderate	Indirectness[2]
BF:	Indirect	Moderate	Indirectness[2]
BG:	Indirect	Moderate	Study limitations[1]; Indirectness[2]; Imprecision[4]
BH:	Indirect	Moderate	Indirectness[2]
BI:	Indirect	Moderate	Indirectness[2]
CE:	Indirect	Low	Indirectness[2]; Imprecision[4]
CF:	Indirect	Low	Study limitations[1]; Indirectness[2]
CG:	Indirect	Low	Study limitations[1]; Indirectness[2]
CH:	Indirect	Moderate	Indirectness[2]
CI:	Indirect	Moderate	Indirectness[2]
DE:	Indirect	Moderate	Indirectness[2]
DF:	Indirect	Moderate	Indirectness[2]
DG:	Indirect	Low	Study limitations[1]; Indirectness[2]
DH:	Indirect	Moderate	Indirectness[2]
DI:	Indirect	Moderate	Indirectness[2]
EF:	Indirect	Moderate	Indirectness[2];
EG:	Indirect	Low	Study limitations[1]; Indirectness[2]
EH:	Indirect	Moderate	Indirectness[2]
EI:	Indirect	Moderate	Indirectness[2]
EI:	Indirect	Moderate	Indirectness[2]
FG:	Indirect	Moderate	Study limitations[1]; Indirectness[2]
FH:	Indirect	Moderate	Indirectness[2]
FI:	Indirect	Moderate	Indirectness[2]
GH:	Indirect	Low	Study limitations[1]; Indirectness[2]
GI:	Indirect	Moderate	Indirectness[2]
HI:	Indirect	Moderate	Indirectness[2]
Ranking of treatments		Moderate	Imprecision[4]

[1]Dominated by evidence at high or moderate risk of bias.

[2]No convincing evidence for the plausibility of the transitivity assumption.

[3]Predictive intervals for treatment effect include effects that would have different interpretations (there is additionally no convincing evidence for the plausibility of the transitivity assumption).

[4]Confidence intervals include values favouring either treatment.

[5]60% of the information is from studies at moderate risk of bias.

[6]Moderate level of heterogeneity, and some evidence of inconsistency in the network.

Note. A means control group; B means interval training; C means aerobic exercise; D means resistance training; E means combined training; F means whole-body vibration training; G means statin drug

Table3 Network meta-analysis matrix of PWV and secondary outcomes.

**PWV, m/s**								
hSTA	0.43 (-0.59,1.45)	0.91 (-0.27,2.09)	0.55 (-0.52,1.62)	0.86 (-0.11,1.84)	0.86 (-0.08,1.79)	0.82 (-0.09,1.73)	0.62 (-0.30,1.55)	**1.39 (0.56,2.22)**
-0.43 (-1.45,0.59)	mSTA	0.47 (-0.55,1.50)	0.12 (-0.78,1.02)	0.43 (-0.35,1.21)	0.43 (-0.31,1.16)	0.39 (-0.31,1.08)	0.19 (-0.53,0.91)	**0.96 (0.36,1.55)**
-0.91 (-2.09,0.27)	-0.47 (-1.50,0.55)	lSTA	-0.36 (-1.43,0.72)	-0.04 (-1.01,0.93)	-0.05 (-0.99,0.89)	-0.09 (-0.99,0.82)	-0.28 (-1.21,0.64)	0.48 (-0.35,1.32)
-0.55 (-1.62,0.52)	-0.12 (-1.02,0.78)	0.36 (-0.72,1.43)	WBV	0.31 (-0.53,1.15)	0.31 (-0.49,1.11)	0.27 (-0.50,1.03)	0.07 (-0.71,0.86)	**0.84 (0.16,1.51)**
-0.86 (-1.84,0.11)	-0.43 (-1.21,0.35)	0.04 (-0.93,1.01)	-0.31 (-1.15,0.53)	CT	-0.01 (-0.66,0.65)	-0.04 (-0.66,0.57)	-0.24 (-0.88,0.40)	**0.52 (0.03,1.02)**
-0.86 (-1.79,0.08)	-0.43 (-1.16,0.31)	0.05 (-0.89,0.99)	-0.31 (-1.11,0.49)	0.01 (-0.65,0.66)	RT	-0.04 (-0.54,0.46)	-0.23 (-0.79,0.32)	**0.53 (0.11,0.96)**
-0.82 (-1.73,0.09)	-0.39 (-1.08,0.31)	0.09 (-0.82,0.99)	-0.27 (-1.03,0.50)	0.04 (-0.57,0.66)	0.04 (-0.46,0.54)	AE	-0.20 (-0.71,0.32)	**0.57 (0.21,0.93)**
-0.62 (-1.55,0.30)	-0.19 (-0.91,0.53)	0.28 (-0.64,1.21)	-0.07 (-0.86,0.71)	0.24 (-0.40,0.88)	0.23 (-0.32,0.79)	0.20 (-0.32,0.71)	INT	**0.77 (0.36,1.17)**
**-1.39 (-2.22,-0.56)**	**-0.96 (-1.55,-0.36)**	-0.48 (-1.32,0.35)	**-0.84 (-1.51,-0.16)**	**-0.52 (-1.02,-0.03)**	**-0.53 (-0.96,-0.11)**	**-0.57 (-0.93,-0.21)**	**-0.77 (-1.17,-0.36)**	CON
**SBP, mm/Hg**								
hSTA	-0.39 (-6.68,5.90)	0.87 (-7.08,8.81)	**-7.88 (-15.18,-0.58)**	-1.92 (-8.14,4.31)	-0.35 (-6.65,5.95)	-3.71 (-9.72,2.31)	**-6.85 (-12.95,-0.75)**	0.11 (-5.42,5.64)
0.39 (-5.90,6.68)	mSTA	1.26 (-5.18,7.69)	**-7.49 (-13.13,-1.85)**	-1.53 (-5.68,2.62)	0.04 (-4.20,4.28)	-3.32 (-7.14,0.50)	**-6.46 (-10.42,-2.51)**	0.50 (-2.47,3.48)
-0.87 (-8.81,7.08)	-1.26 (-7.69,5.18)	lSTA	**-8.74 (-16.19,-1.30)**	-2.78 (-9.18,3.61)	-1.22 (-7.67,5.24)	-4.57 (-10.76,1.61)	**-7.72 (-13.99,-1.45)**	-0.75 (-6.46,4.96)
**7.88 (0.58,15.18)**	**7.49 (1.85,13.13)**	**8.74 (1.30,16.19)**	WBV	**5.96 (0.38,11.54)**	**7.53 (1.88,13.18)**	4.17 (-1.17,9.51)	1.03 (-4.41,6.47)	**7.99 (3.21,12.77)**
1.92 (-4.31,8.14)	1.53 (-2.62,5.68)	2.78 (-3.61,9.18)	**-5.96 (-11.54,-0.38)**	CT	1.57 (-2.61,5.74)	-1.79 (-5.53,1.95)	**-4.93 (-8.82,-1.05)**	2.03 (-0.85,4.92)
0.35 (-5.95,6.65)	-0.04 (-4.28,4.20)	1.22 (-5.24,7.67)	**-7.53 (-13.18,-1.88)**	-1.57 (-5.74,2.61)	RT	-3.36 (-6.85,0.14)	**-6.50 (-10.47,-2.54)**	0.46 (-2.55,3.48)
3.71 (-2.31,9.72)	3.32 (-0.50,7.14)	4.57 (-1.61,10.76)	-4.17 (-9.51,1.17)	1.79 (-1.95,5.53)	3.36 (-0.14,6.85)	AE	-3.15 (-6.58,0.29)	**3.82 (1.43,6.21)**
**6.85 (0.75,12.95)**	**6.46 (2.51,10.42)**	**7.72 (1.45,13.99)**	-1.03 (-6.47,4.41)	**4.93 (1.05,8.82)**	**6.50 (2.54,10.47)**	3.15 (-0.29,6.58)	INT	**6.97 (4.37,9.57)**
-0.11 (-5.64,5.42)	-0.50 (-3.48,2.47)	0.75 (-4.96,6.46)	**-7.99 (-12.77,-3.21)**	-2.03 (-4.92,0.85)	-0.46 (-3.48,2.55)	**-3.82 (-6.21,-1.43)**	**-6.97 (-9.57,-4.37)**	CON
**DBP, mm/Hg**								
hSTA	-0.27 (-6.09,5.56)	-1.87 (-8.97,5.23)	-2.50 (-9.32,4.31)	-0.86 (-6.75,5.03)	2.52 (-3.26,8.30)	-0.89 (-6.43,4.65)	-1.30 (-6.86,4.27)	1.25 (-3.81,6.31)

0.27 (-5.56,6.09)	**mSTA**	-1.60 (-7.36,4.15)	-2.23 (-7.63,3.16)	-0.59 (-4.77,3.58)	2.79 (-1.23,6.80)	-0.63 (-4.29,3.03)	-1.03 (-4.72,2.67)	1.51 (-1.37,4.40)
1.87 (-5.23,8.97)	1.60 (-4.15,7.36)	**lSTA**	-0.63 (-7.39,6.13)	1.01 (-4.82,6.84)	4.39 (-1.33,10.10)	0.98 (-4.50,6.45)	0.57 (-4.92,6.07)	3.12 (-1.87,8.10)
2.50 (-4.31,9.32)	2.23 (-3.16,7.63)	0.63 (-6.13,7.39)	**WBV**	1.64 (-3.83,7.11)	5.02 (-0.33,10.37)	1.61 (-3.48,6.70)	1.21 (-3.91,6.32)	3.75 (-0.82,8.31)
0.86 (-5.03,6.75)	0.59 (-3.58,4.77)	-1.01 (-6.84,4.82)	-1.64 (-7.11,3.83)	**CT**	3.38 (-0.73,7.48)	-0.03 (-3.80,3.73)	-0.44 (-4.24,3.37)	2.11 (-0.91,5.13)
-2.52 (-8.30,3.26)	-2.79 (-6.80,1.23)	-4.39 (-10.10,1.33)	-5.02 (-10.37,0.33)	-3.38 (-7.48,0.73)	**RT**	**-3.41 (-6.67,-0.15)**	**-3.81 (-7.42,-0.20)**	-1.27 (-4.06,1.52)
0.89 (-4.65,6.43)	0.63 (-3.03,4.29)	-0.98 (-6.45,4.50)	-1.61 (-6.70,3.48)	0.03 (-3.73,3.80)	**3.41 (0.15,6.67)**	**AE**	-0.40 (-3.54,2.74)	2.14 (-0.11,4.40)
1.30 (-4.27,6.86)	1.03 (-2.67,4.72)	-0.57 (-6.07,4.92)	-1.21 (-6.32,3.91)	0.44 (-3.37,4.24)	**3.81 (0.20,7.42)**	0.40 (-2.74,3.54)	**INT**	**2.54 (0.23,4.86)**
-1.25 (-6.31,3.81)	-1.51 (-4.40,1.37)	-3.12 (-8.10,1.87)	-3.75 (-8.31,0.82)	-2.11 (-5.13,0.91)	1.27 (-1.52,4.06)	-2.14 (-4.40,0.11)	**-2.54 (-4.86,-0.23)**	**CON**

Efficacy (response rate) Comparison Acceptability (dropout rate) Group

Note: Effects are expressed as the effect size [95% CI] between interventions. Bold indicates that the data are significant, light cyan areas indicate the effect of the longitudinal versus the lateral intervention, pink areas indicate the effect of the lateral versus the longitudinal intervention, grey areas represent the intervention category, and black areas represent the group. For example, "-0.77 (-1.17,-0.36)" (column 8, row 8), which indicates that INT (longitudinal intervention) significantly reduces PWV compared with CON (transverse intervention).

Note. CON, control group; INT, interval training; AE, aerobic exercise; RT, resistance training; CT, combined training; WBV, whole-body vibration training; lSTA, low doses of statins; mSTA, moderate doses of statins; hSTA, high doses of statins.

Appendix 15. Subgroup analysis of Pulse Wave Velocity in intensity

Appendix 15.1 Forest plot of pairwise meta-analysis for pulse wave velocity in different intensity.

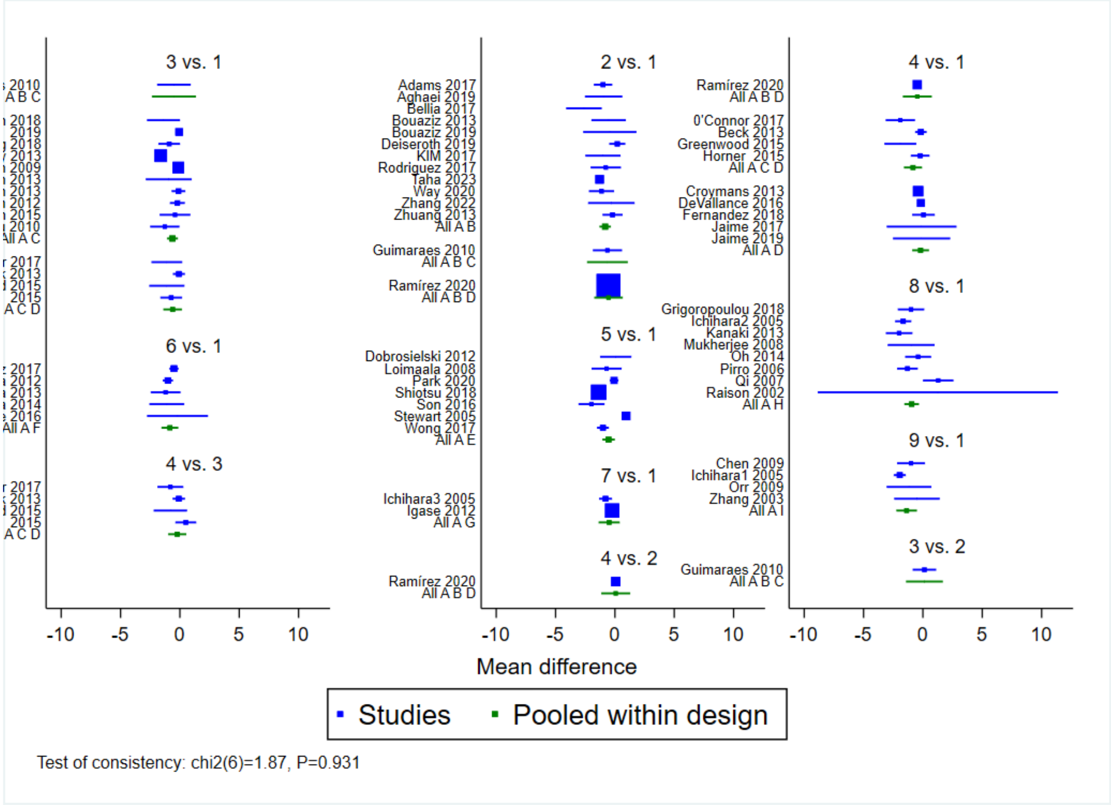

Appendix 15.1.1 Forest plot of pairwise meta-analysis for pulse wave velocity in high intensity

Note. 1 means interval training; 2 means aerobic exercise; 3 means resistance exercise; 4 means combined training; 5 means control group; 6 means whole-body vibration training; 7 means low doses of statins; 8 means moderate doses of statins; 9 means high doses of statins.

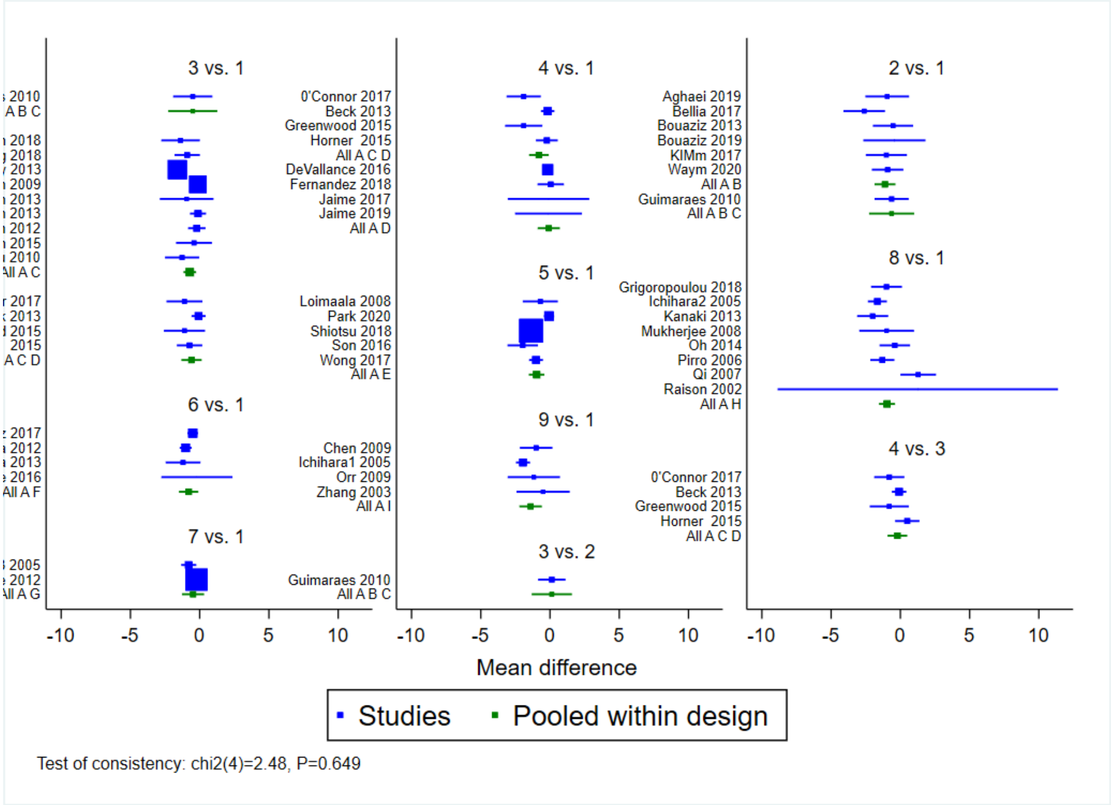

Appendix 15.1.2 Forest plot of pairwise meta-analysis for pulse wave velocity in moderate intensity

Note. 1 means interval training; 2 means aerobic exercise; 3 means resistance exercise; 4 means combined training; 5 means control group; 6 means whole-body vibration training; 7 means low doses of statins; 8 means moderate doses of statins; 9 means high doses of statins.

Appendix 15.2 Network of eligible comparisons for pulse wave velocity in different intensity.

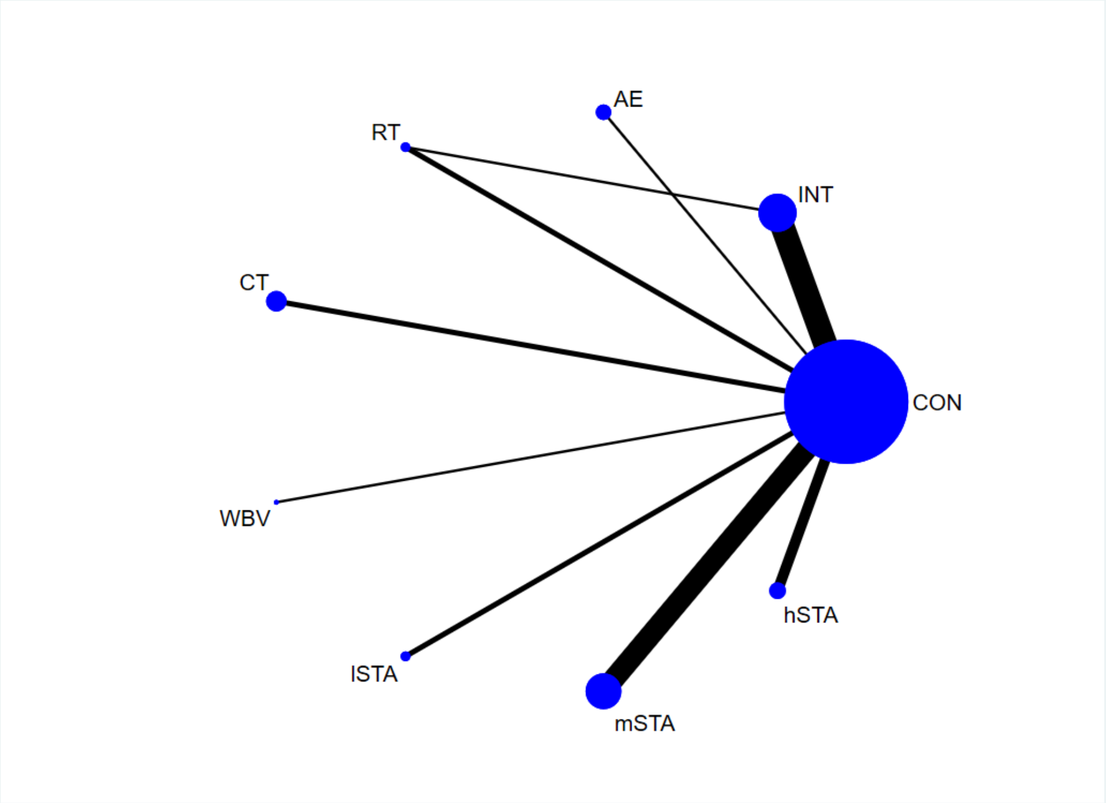

Appendix 15.2.1 Network of eligible comparisons for pulse wave velocity in high intensity

Note. CON, control group; INT, interval training; AE, means aerobic exercise; RT, resistance training; CT, combined training; WBV, whole-body vibration training; lSTA, low doses of statins; mSTA, moderate doses of statins; hSTA, high doses of statins.

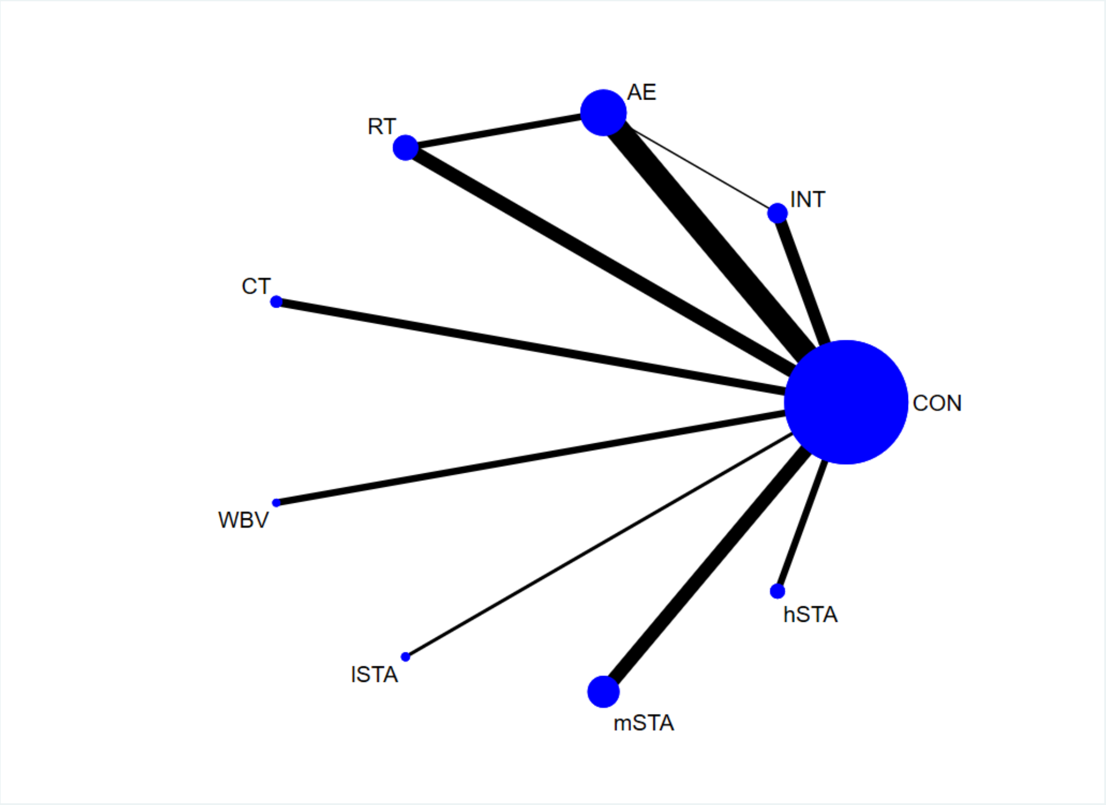

Appendix 15.2.2 Network of eligible comparisons for pulse wave velocity in moderate intensity

Note. CON, control group; INT, interval training; AE, aerobic exercise; RT, resistance training; CT, combined training; WBV, whole-body vibration training; lSTA, low doses of statins; mSTA, moderate doses of statins; hSTA, high doses of statins.

Appendix 15.3 Inconsistency model for Pulse Wave Velocity in different intensity.

	Pulse Wave Velocity<br>High intensity	Pulse Wave Velocity<br>Low intensity
chi2	0.07	2.48
Prob > chi2	0.9658	6.4890

No evidence for the existence of significant global inconsistency

Appendix 15.4 Loop-specific heterogeneity for pulse wave velocity in different intensity.

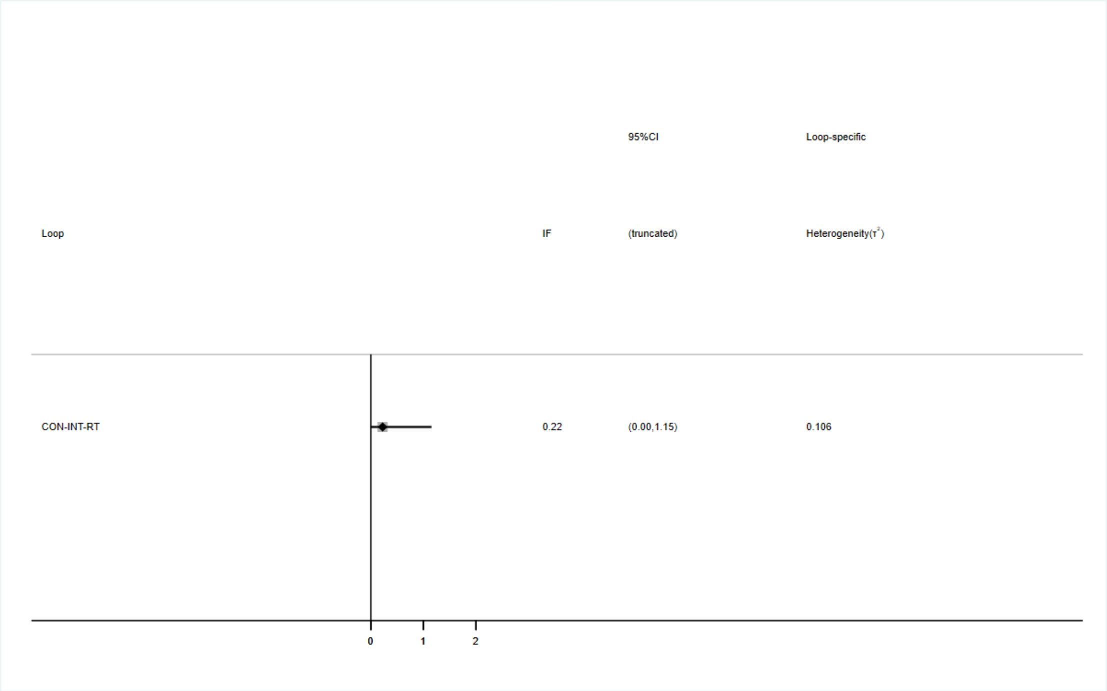

Appendix 15.4.1 Loop-specific heterogeneity for pulse wave velocity.

Note. CON means non-exercise control; RT means resistance training; INT means interval training.

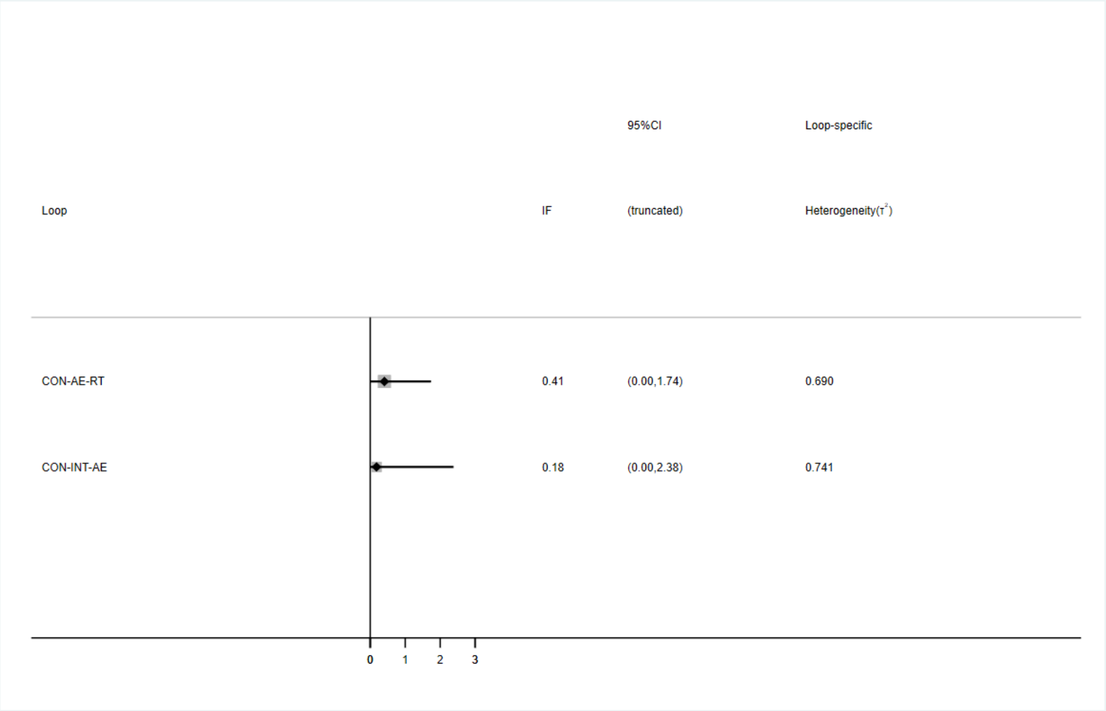

Appendix 15.4.2 Loop-specific heterogeneity for pulse wave velocity.

Note. CON means non-exercise control; AE, aerobic exercise RT means resistance training; INT means interval training.

Appendix 15.5 Local inconsistency test for Pulse Wave Velocity in different intensity

Side	Direct Coef.	Std. Err.	Indirect Coef.	Std. Err.	Difference Coef.	Std.Err.	P>z
			High intensity				
A B	-0.651	0.229	-0.400	1.426	-0.252	1.444	0.862
A D	-0.435	0.377	-0.744	1.042	0.309	1.103	0.779
B D	0.069	0.534	0.290	0.553	-0.221	0.765	0.773
			Moderate intensity				
A B	-1.026	0.347	-0.722	1.436	-0.304	1.509	0.841
A C	-0.673	0.189	0.104	0.832	-0.777	0.855	0.363
A D	-0.487	0.266	-1.082	0.762	0.595	0.812	0.464
B C	0.100	0.728	0.461	0.421	-0.361	0.847	0.670
C D	-0.218	0.351	0.519	0.426	-0.737	0.555	0.184

Appendix 15.6 Prediction interval forest plots for Pulse Wave Velocity in different intensity

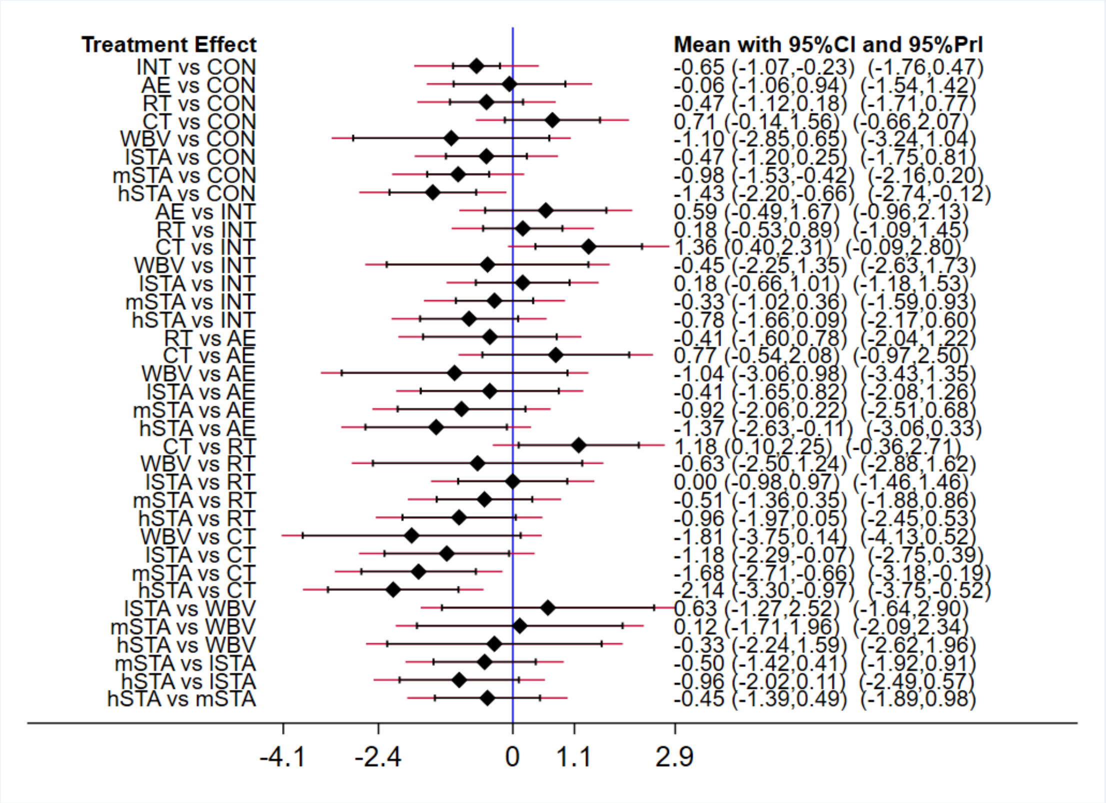

Appendix 15.6.1 Prediction interval forest plots for Pulse Wave Velocity in high intensity

Note. CON, control group; INT, interval training; AE, aerobic exercise; RT, resistance training; CT, combined training; WBV, whole-body vibration training; lSTA, low doses of statins; mSTA, moderate doses of statins; hSTA, high doses of statins.

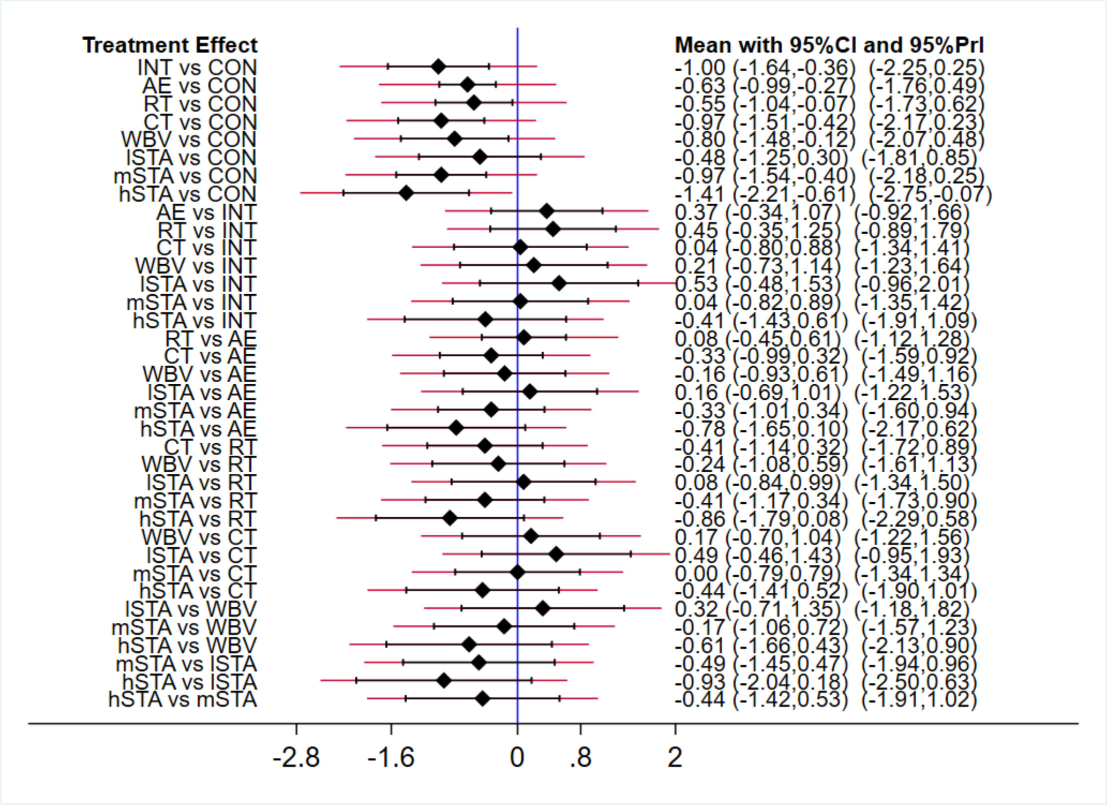

Appendix 15.6.2 Prediction interval forest plots for Pulse Wave Velocity in modrate intensity

Note. CON, control group; INT, interval training; AE, aerobic exercise; RT, resistance training; CT, combined training; WBV, whole-body vibration training; lSTA, low doses of statins; mSTA, moderate doses of statins; hSTA, high doses of statins.

Appendix 15.7 Contributions of direct and indirect comparisons to NMA and the number of studies of each direct comparison of Pulse Wave Velocity in different intensity.

Note. A means control group; B means interval training; C means aerobic exercise; D means resistance training; E means combined training; F means whole-body vibration training; G means low doses of statins; H means moderate doses of statins; I means high doses of statins.

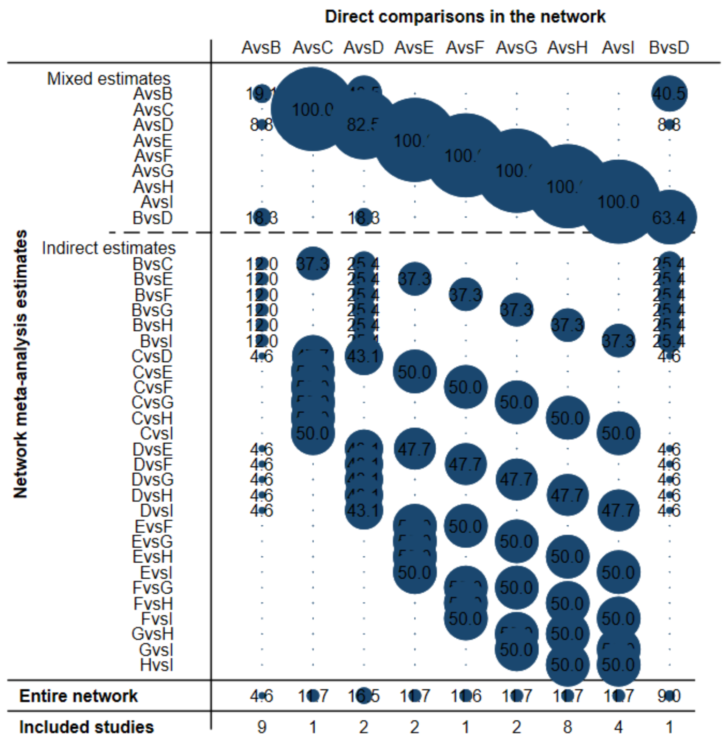

Appendix 15.7.1 Contribution plot for pulse wave velocity in high intensity.

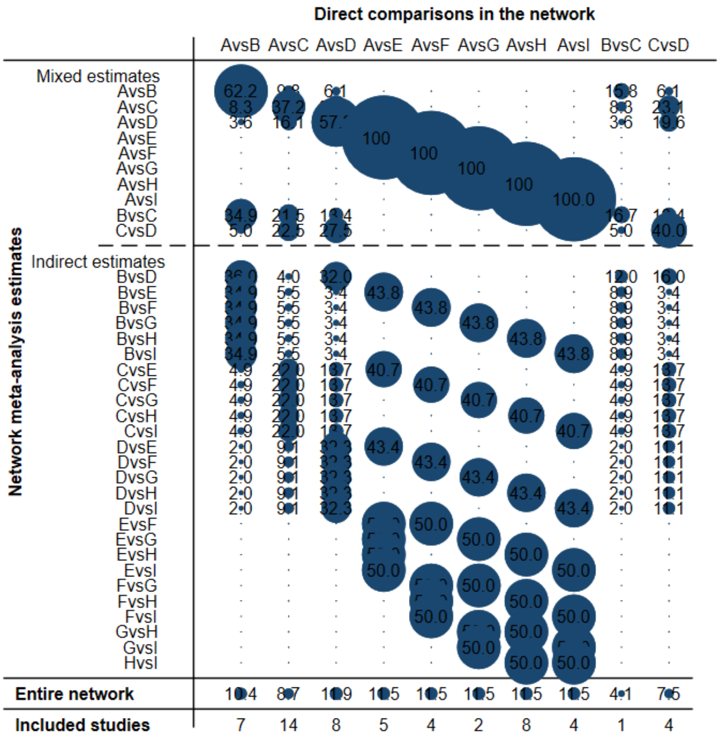

Appendix 15.7.2 Contribution plot for pulse wave velocity in moderate intensity.

Appendix 15.8 The funnel plot graphics of Pulse Wave Velocity in indifferent intensity.

Note. A means control group; B means interval training; C means aerobic exercise; D means resistance training; E means combined training; F means whole-body vibration training; G means low doses of statins; H means moderate doses of statins; I means high doses of statins.

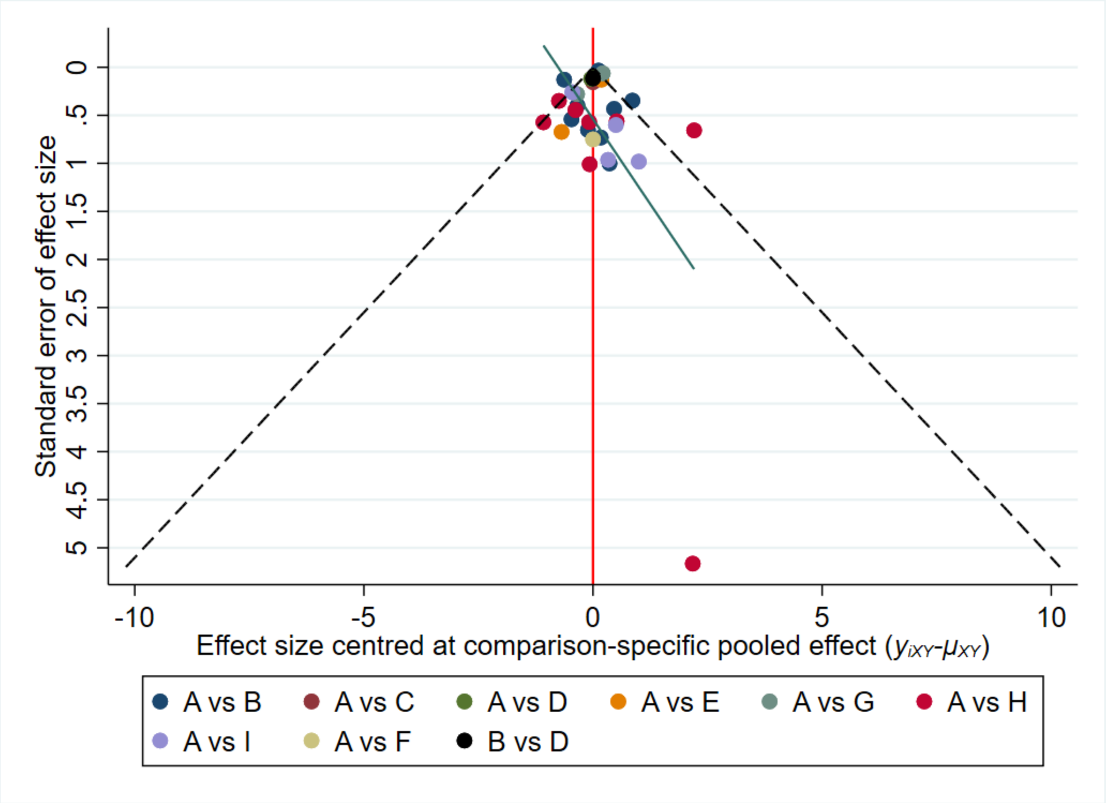

Appendix 15.8.1 Comparison-adjusted funnel plot for pulse wave velocity in high intensity

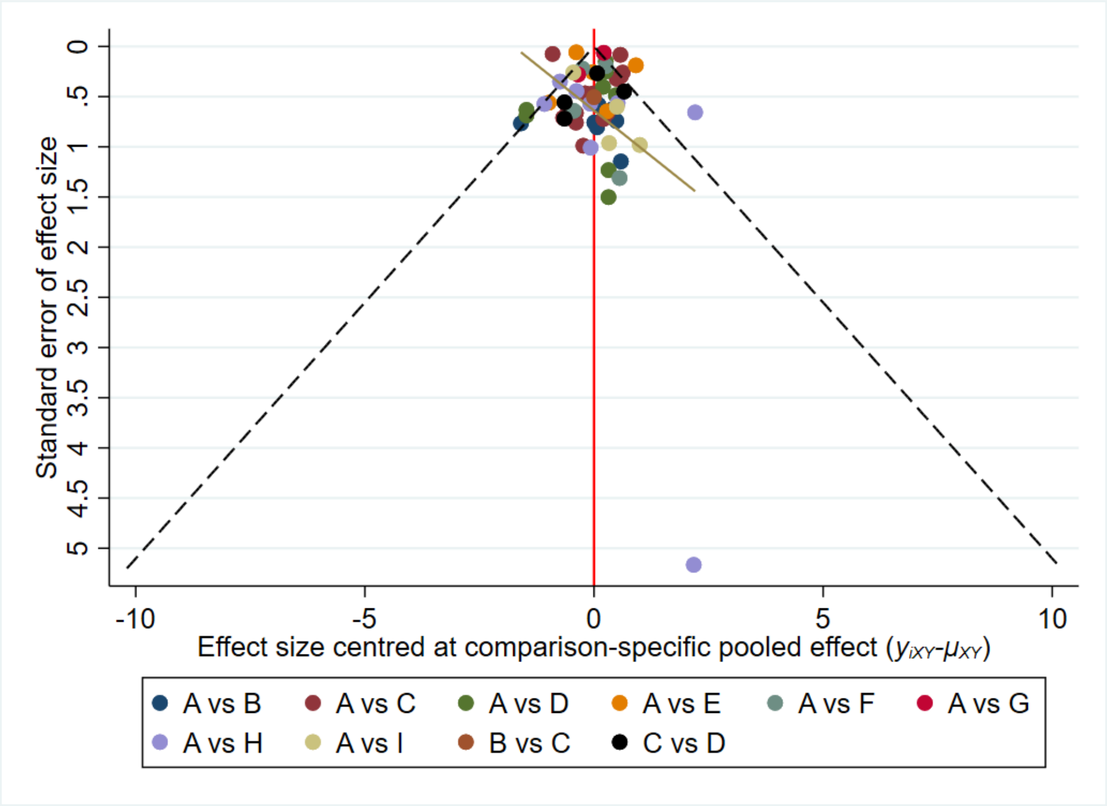

Appendix 15.8.2 Comparison-adjusted funnel plot for pulse wave velocity in moderate intensity

Appendix 15.9 Ranking of the effects of different types of exercise on pulse wave velocity in different intensity

Note. CON, control group; INT, interval training; AE, aerobic exercise; RT, resistance training; CT, combined training; WBV, whole-body vibration training; lSTA, low doses of statins; mSTA, moderate doses of statins; hSTA, high doses of statins.

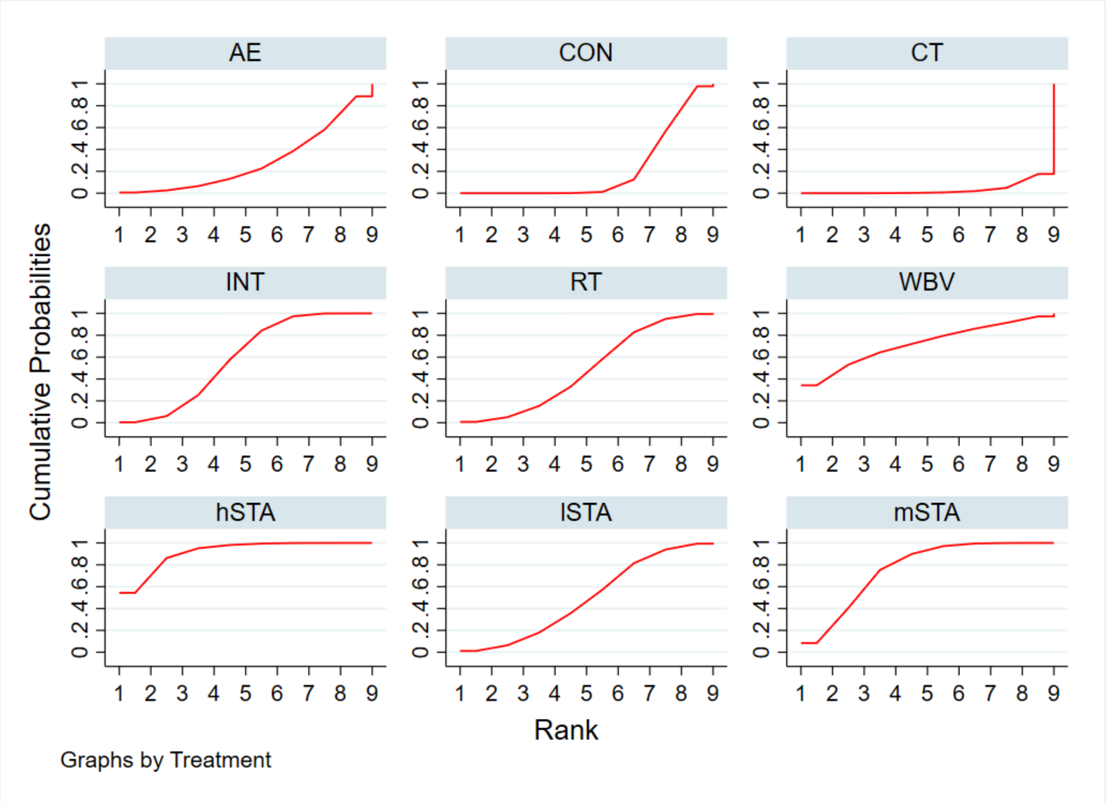

Appendix 15.9.1 Ranking of the effects of different types of exercise on pulse wave velocity in high intensity

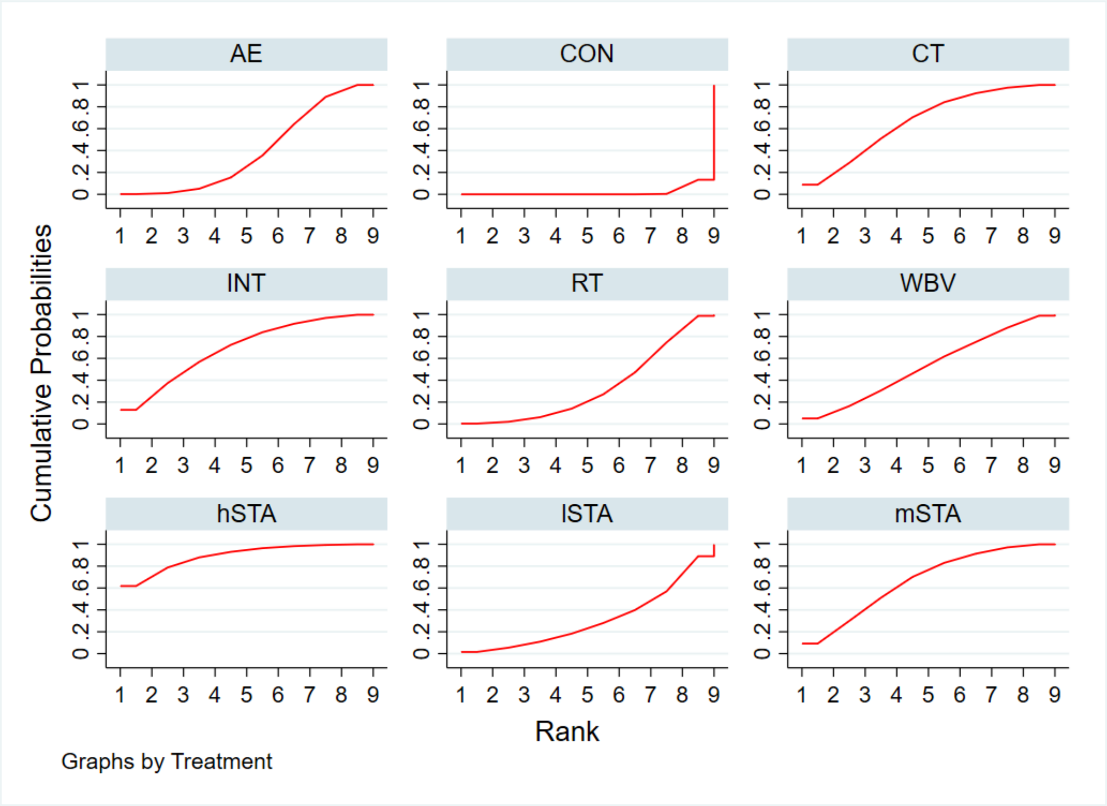

Appendix 15.9.2 Ranking of the effects of different types of exercise on pulse wave velocity in moderate intensity

Appendix 15.10 The ranking of effects of different exercise on pulse wave velocity indifferent intensity.

**High intensity (49studies, N=1892)**			
**Treatment**	SUCRA	PrBest (%)	Mean Rank
hSTA	91.6	54.3	1.7
mSTA	76.3	8.4	2.9
WBV	72.2	34.2	3.2
INT	58.9	0.4	4.3
lSTA	49.2	1.2	5.1
RT	48.7	0.8	5.1
AE	28.8	0.6	6.7
CON	21	0	7.3
CT	3.2	0	8.7
**Moderate intensity (24studies, N=1711)**			
**Treatment**	SUCRA	PrBest (%)	Mean Rank
hSTA	89.5	61.9	1.8
INT	69	12.9	3.5
CT	66.6	8.8	3.7
mSTA	66.5	9.2	3.7
WBV	52.8	5.1	4.8
AE	38.8	0.2	5.9
RT	33.8	0.3	6.3
lSTA	31.3	1.5	6.5
CON	1.7	0	8.9

Note. CON, control group; INT, interval training; AE, aerobic exercise; RT, resistance training; CT, combined training; WBV, whole-body vibration training; lSTA, low doses of statins; mSTA, moderate doses of statins; hSTA, high doses of statins.

Appendix 15.11 Network meta-analysis matrix of PWV in different intensity.

**PWV in High intensity, m/s**								
**hSTA**	0.45 (-0.49,1.39)	0.96 (-0.11,2.02)	0.33 (-1.59,2.24)	**2.14 (0.97,3.30)**	0.96 (-0.05,1.97)	**1.37 (0.11,2.63)**	0.78 (-0.09,1.66)	**1.43 (0.66,2.20)**
-0.45 (-1.39,0.49)	**mSTA**	0.50 (-0.41,1.42)	-0.12 (-1.96,1.71)	**1.68 (0.66,2.71)**	0.51 (-0.35,1.36)	0.92 (-0.22,2.06)	0.33 (-0.36,1.02)	**0.98(0.42,1.53)**
-0.96 (-2.02,0.11)	-0.50 (-1.42,0.41)	**lSTA**	-0.63 (-2.52,1.27)	**1.18 (0.07,2.29)**	0.00 (-0.97,0.98)	0.41 (-0.82,1.65)	-0.18 (-1.01,0.66)	0.47 (-0.25,1.20)
-0.33 (-2.24,1.59)	0.12 (-1.71,1.96)	0.63 (-1.27,2.52)	**WBV**	1.81 (-0.14,3.75)	0.63 (-1.24,2.50)	1.04 (-0.98,3.06)	0.45 (-1.35,2.25)	1.10 (-0.65,2.85)
**-2.14 (-3.30, -0.97)**	**-1.68 (-2.71, -0.66)**	**-1.18 (-2.29,-0.07)**	-1.81 (-3.75,0.14)	**CT**	-1.18 (-2.25,-0.10)	-0.77 (-2.08,0.54)	**-1.36 (-2.31, -0.40)**	-0.71 (-1.56,0.14)
-0.96 (-1.97,0.05)	-0.51 (-1.36,0.35)	-0.00 (-0.98,0.97)	-0.63 (-2.50,1.24)	1.18 (0.10,2.25)	**RT**	0.41 (-0.78,1.60)	-0.18 (-0.89,0.53)	0.47 (-0.18,1.12)
**-1.37 (-2.63, -0.11)**	-0.92 (-2.06,0.22)	-0.41 (-1.65,0.82)	-1.04 (-3.06,0.98)	0.77 (-0.54,2.08)	-0.41 (-1.60,0.78)	**AE**	-0.59 (-1.67,0.49)	0.06 (-0.94,1.06)
-0.78 (-1.66,0.09)	-0.33 (-1.02,0.36)	0.18 (-0.66,1.01)	-0.45 (-2.25,1.35)	**1.36 (0.40,2.31)**	0.18 (-0.53,0.89)	0.59 (-0.49,1.67)	**INT**	**0.65 (0.23,1.07)**
**-1.43 (-2.20, -0.66)**	**-0.98 (-1.53, -0.42)**	-0.47 (-1.20,0.25)	-1.10 (-2.85,0.65)	0.71 (-0.14,1.56)	-0.47 (-1.12,0.18)	-0.06 (-1.06,0.94)	**-0.65 (-1.07, -0.23)**	**CON**
**PWV in Moderate intensity, m/s**								
**hSTA**	0.44 (-0.53,1.42)	0.93 (-0.18,2.04)	0.61 (-0.43,1.66)	0.44 (-0.52,1.41)	0.86 (-0.08,1.79)	0.78 (-0.10,1.65)	0.41 (-0.61,1.43)	**1.41 (0.61,2.21)**
-0.44 (-1.42,0.53)	**mSTA**	0.49 (-0.47,1.45)	0.17 (-0.72,1.06)	0.00 (-0.79,0.79)	0.41 (-0.34,1.17)	0.33 (-0.34,1.01)	-0.04 (-0.89,0.82)	**0.97 (0.40,1.54)**
-0.93 (-2.04,0.18)	-0.49 (-1.45,0.47)	**lSTA**	-0.32 (-1.35,0.71)	-0.49 (-1.43,0.46)	-0.08 (-0.99,0.84)	-0.16 (-1.01,0.69)	-0.53 (-1.53,0.48)	0.48 (-0.30,1.25)
-0.61 (-1.66,0.43)	-0.17 (-1.06,0.72)	0.32 (-0.71,1.35)	**WBV**	-0.17 (-1.04,0.70)	0.24 (-0.59,1.08)	0.16 (-0.61,0.93)	-0.21 (-1.14,0.73)	**0.80 (0.12,1.48)**
-0.44 (-1.41,0.52)	-0.00 (-0.79,0.79)	0.49 (-0.46,1.43)	0.17 (-0.70,1.04)	**CT**	0.41 (-0.32,1.14)	0.33 (-0.32,0.99)	-0.04 (-0.88,0.80)	**0.97 (0.42,1.51)**
-0.86 (-1.79,0.08)	-0.41 (-1.17,0.34)	0.08 (-0.84,0.99)	-0.24 (-1.08,0.59)	-0.41 (-1.14,0.32)	**RT**	-0.08 (-0.61,0.45)	-0.45 (-1.25,0.35)	**0.55 (0.07,1.04)**
-0.78 (-1.65,0.10)	-0.33 (-1.01,0.34)	0.16 (-0.69,1.01)	-0.16 (-0.93,0.61)	-0.33 (-0.99,0.32)	0.08 (-0.45,0.61)	**AE**	-0.37 (-1.07,0.34)	**0.63 (0.27,0.99)**
-0.41 (-1.43,0.61)	0.04 (-0.82,0.89)	0.53 (-0.48,1.53)	0.21 (-0.73,1.14)	0.04 (-0.80,0.88)	0.45 (-0.35,1.25)	0.37 (-0.34,1.07)	**INT**	**1.00 (0.36,1.64)**
**-1.41 (-2.21,-0.61)**	**-0.97 (-1.54,-0.40)**	-0.48 (-1.25,0.30)	**-0.80 (-1.48,-0.12)**	**-0.97 (-1.51,-0.42)**	**-0.55 (-1.04,-0.07)**	**-0.63 (-0.99,-0.27)**	**-1.00 (-1.64,-0.36)**	**CON**

Efficacy (response rate) Comparison Acceptability (dropout rate) Group

Note: Effects are expressed as the effect size [95% CI] between interventions. Bold indicates that the data are significant, light cyan areas indicate the effect of the longitudinal versus the lateral intervention, pink areas indicate the effect of the lateral versus the longitudinal intervention, grey areas represent the intervention category, and black areas represent the group. For example, "-1.00 (-1.64,-0.36)" (column 8, row 8), which indicates that INT (longitudinal intervention) significantly reduces PWV compared with CON (transverse intervention).

Note. CON, control group; INT, interval training; AE, aerobic exercise; RT, resistance training; CT, combined training; WBV, whole-body vibration training; lSTA, low doses of statins; mSTA, moderate doses of statins; hSTA, high doses of statins.

Appendix 16 Subgroup analysis of Pulse Wave Velocity in intervention duration

Intervention Cycle Standards: short-term intervention: total intervention time <1000 mins; medium-term intervention: total intervention time 1000-2000 mins; long-term intervention: total intervention time >2000 mins; Whole-body vibration training: 60 mins, 180 mins, 360 mins; Statins therapy correspondence <12 weeks, 12-24 weeks, >24 weeks.

Appendix 16.1 Forest plot of pairwise meta-analysis for pulse wave velocity in different intervention duration.

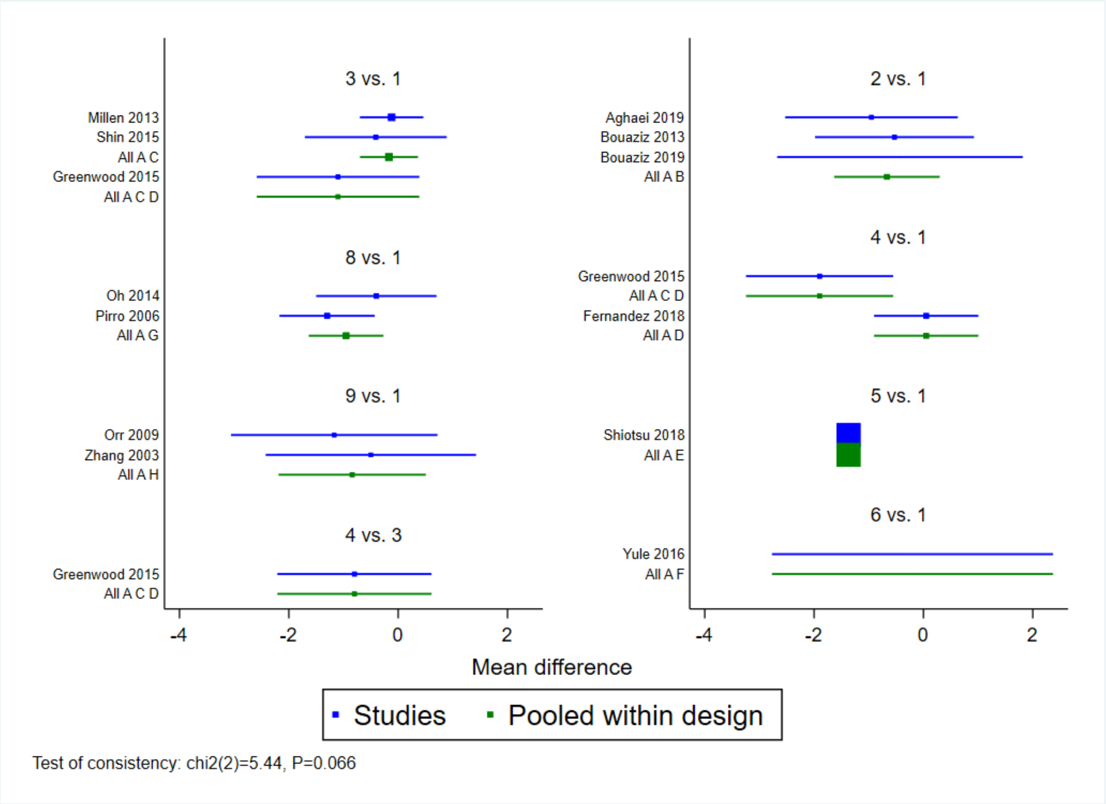

Appendix 16.1.1 Forest plot of pairwise meta-analysis for pulse wave velocity in short-term intervention

Note. 1 means interval training; 2 means aerobic exercise; 3 means resistance exercise; 4 means combined training; 5 means control group; 6 means whole-body vibration training; 8 means moderate doses of statins; 9 means high doses of statins.

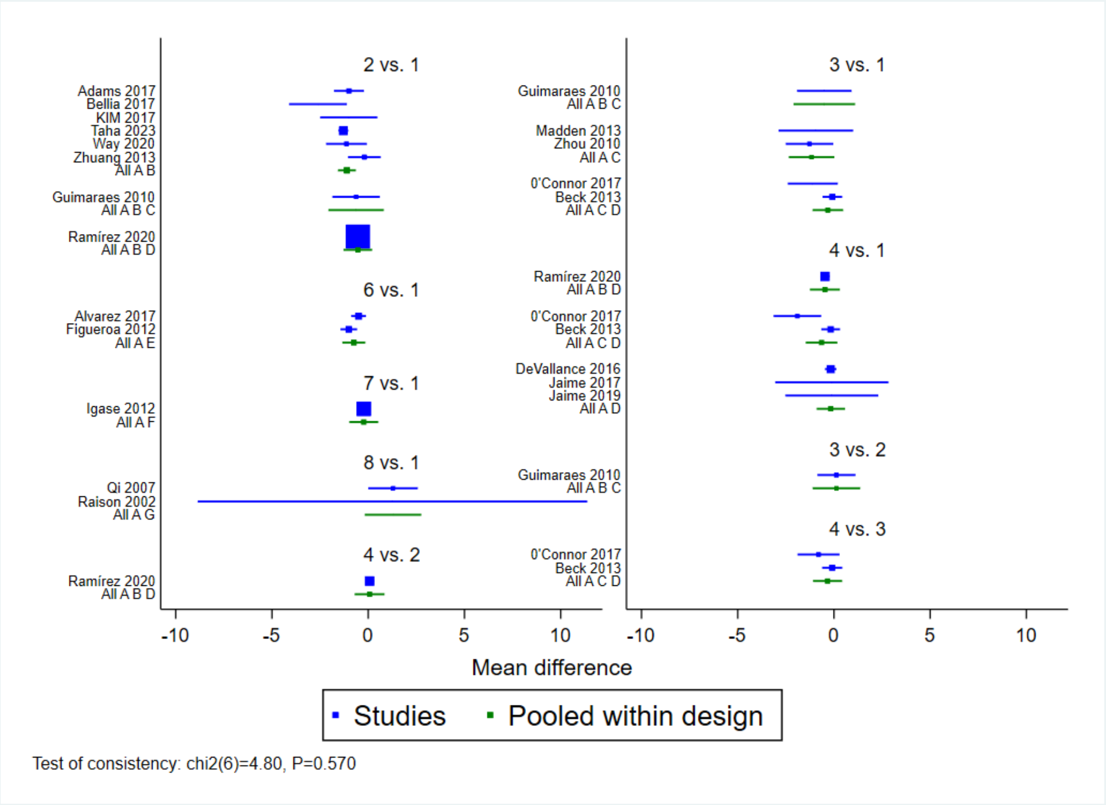

Appendix 16.1.2 Forest plot of pairwise meta-analysis for pulse wave velocity in medium-term intervention

Note. 1 means interval training; 2 means aerobic exercise; 3 means resistance exercise; 4 means combined training; 5 means control group; 6 means whole-body vibration training; 7 means low doses of statins; 8 means moderate doses of statins.

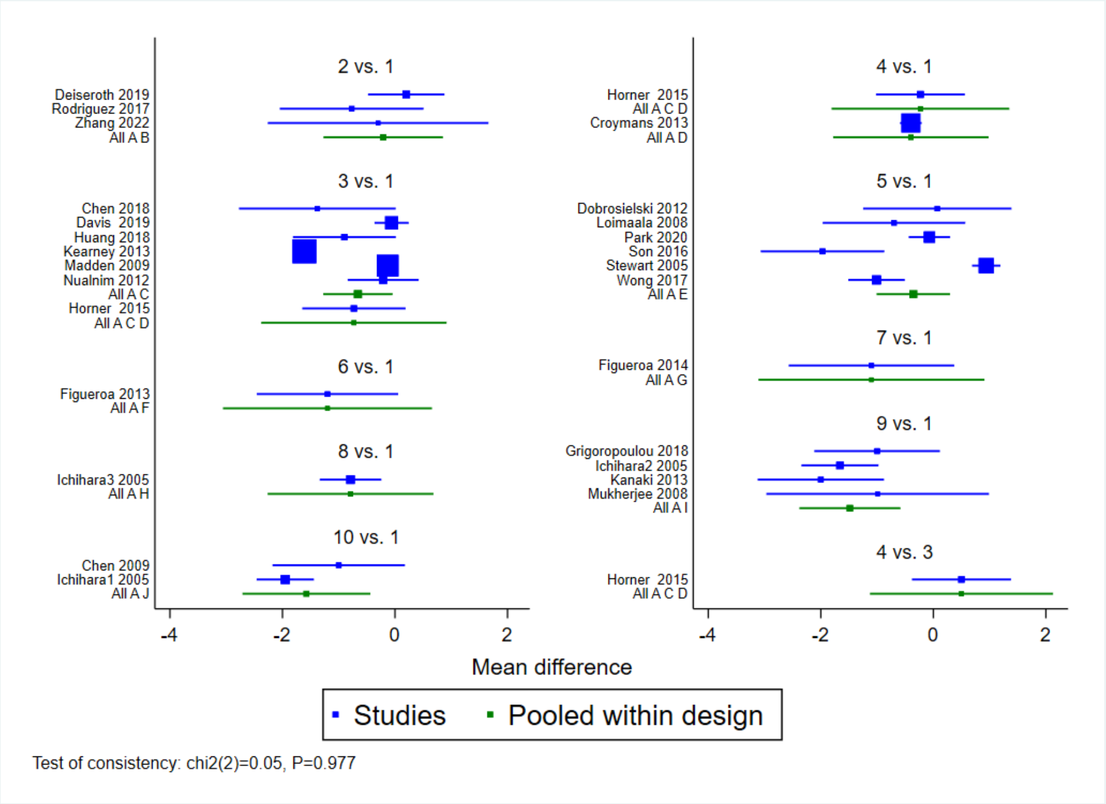

Appendix 16.1.2 Forest plot of pairwise meta-analysis forpulse wave velocity long-term intervention

Note. 1 means interval training; 2 means aerobic exercise; 3means resistance exercise; 4 means combined training; 5 means control group; 6 means low intensity whole-body vibration training; 7 means high intensity whole-body vibration training; 8 means low doses of statins; 9 means moderate doses of statins; 10 means high doses of statins.

Appendix 16.2 Network of eligible comparisons for pulse wave velocity in different intervene durations.

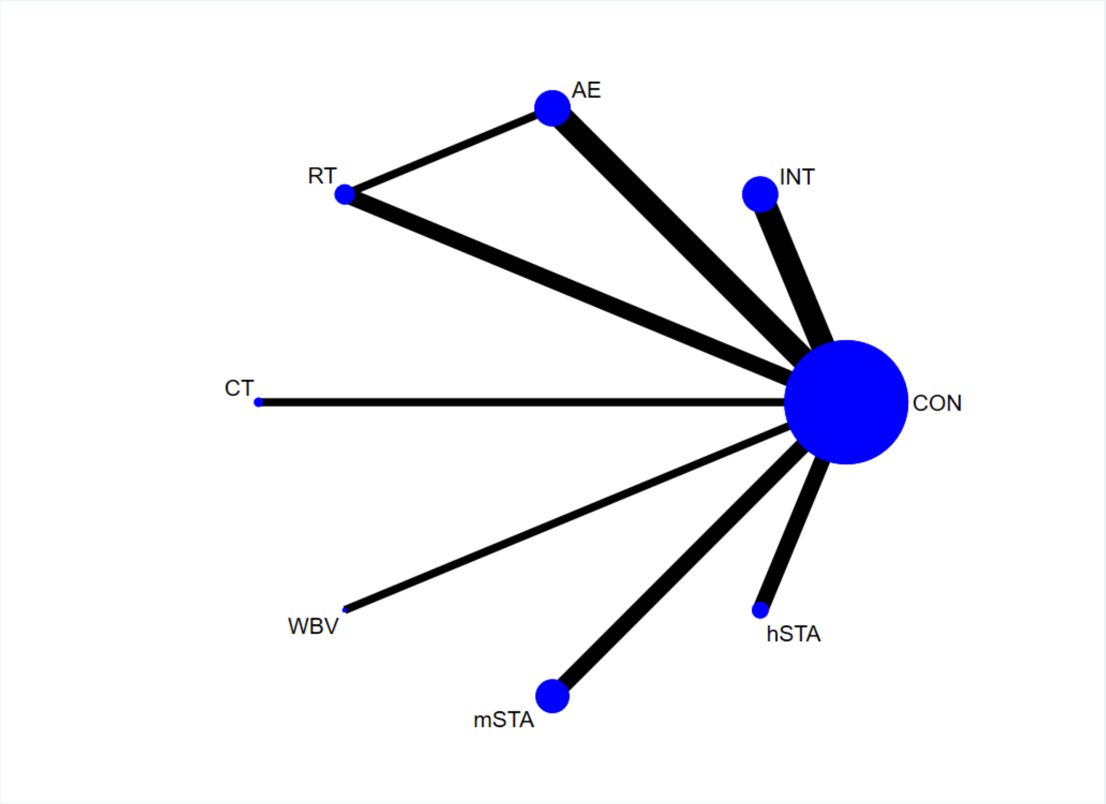

Appendix 16.2.1 Network of eligible comparisons for pulse wave velocity in low-term intervention

Note. CON, control group; INT, interval training; AE, means aerobic exercise; RT, resistance training; CT, combined training; WBV, whole-body vibration training; lSTA, moderate doses of statins; hSTA, high doses of statins.

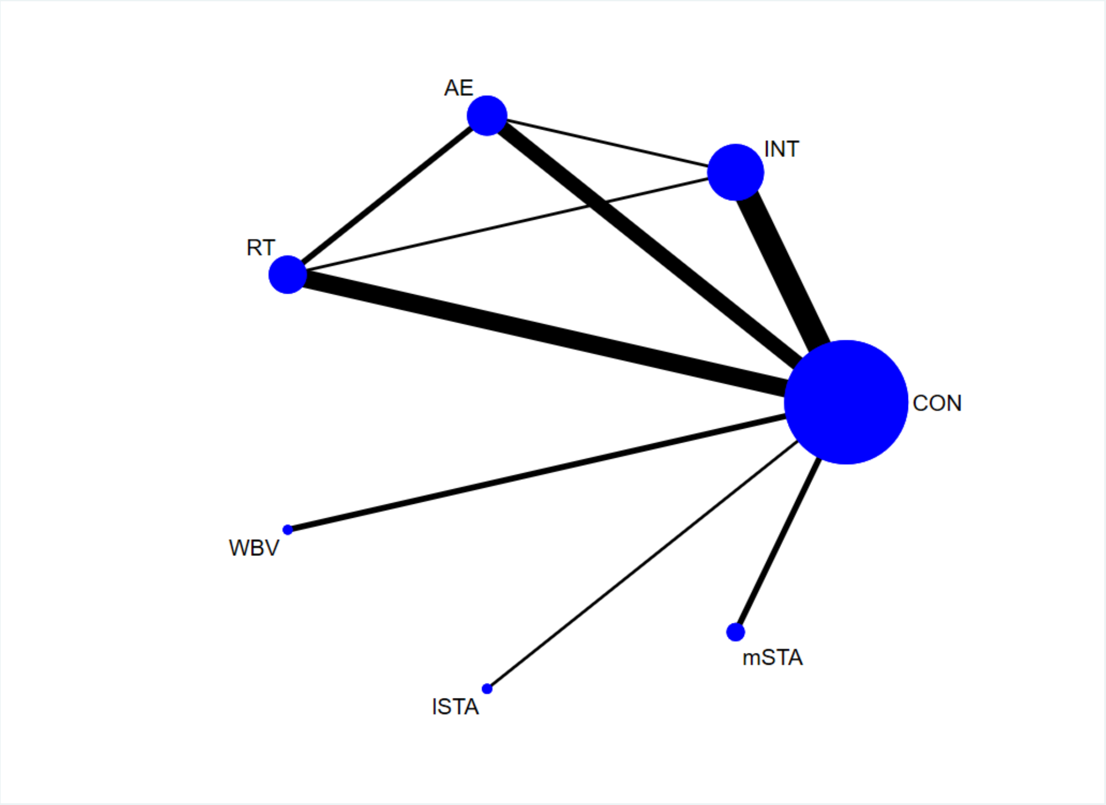

Appendix 16.2.2 Network of eligible comparisons for pulse wave velocity in medium-term intervention

Note. CON, control group; INT, interval training; AE, aerobic exercise; RT, resistance training; CT, combined training; WBV, whole-body vibration training; lSTA, low doses of statins; mSTA, moderate doses of statins.

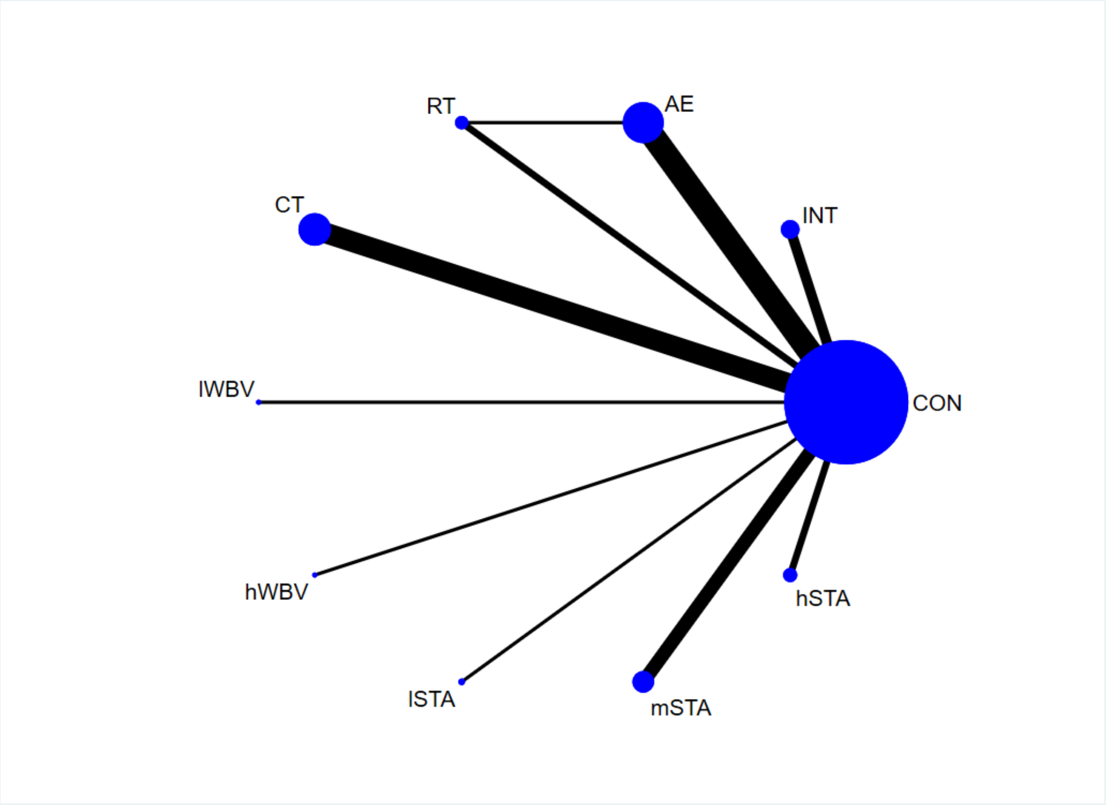

Appendix 16.2.2 Network of eligible comparisons for pulse wave velocity in long-term intervention

Note. CON, control group; INT, interval training; AE, aerobic exercise; RT, resistance training; CT, combined training; lWBV, low intensity whole-body vibration training; hWBV means high intensity whole-body vibration training; lSTA, low doses of statins; mSTA, moderate doses of statins; hSTA means high doses of statins.

Appendix 16.3 Inconsistency model for Pulse Wave Velocity in different intervene duration.

	Pulse Wave Velocity	Systolic Blood Pressure	Diastolic Blood Pressure
chi2	5.44	4.8	0.05
Prob > chi2	0.0657	0.5702	0.9773

No evidence for the existence of significant global inconsistency

Appendix 16.4 Loop-specific heterogeneity for pulse wave velocity in different intervene duration.

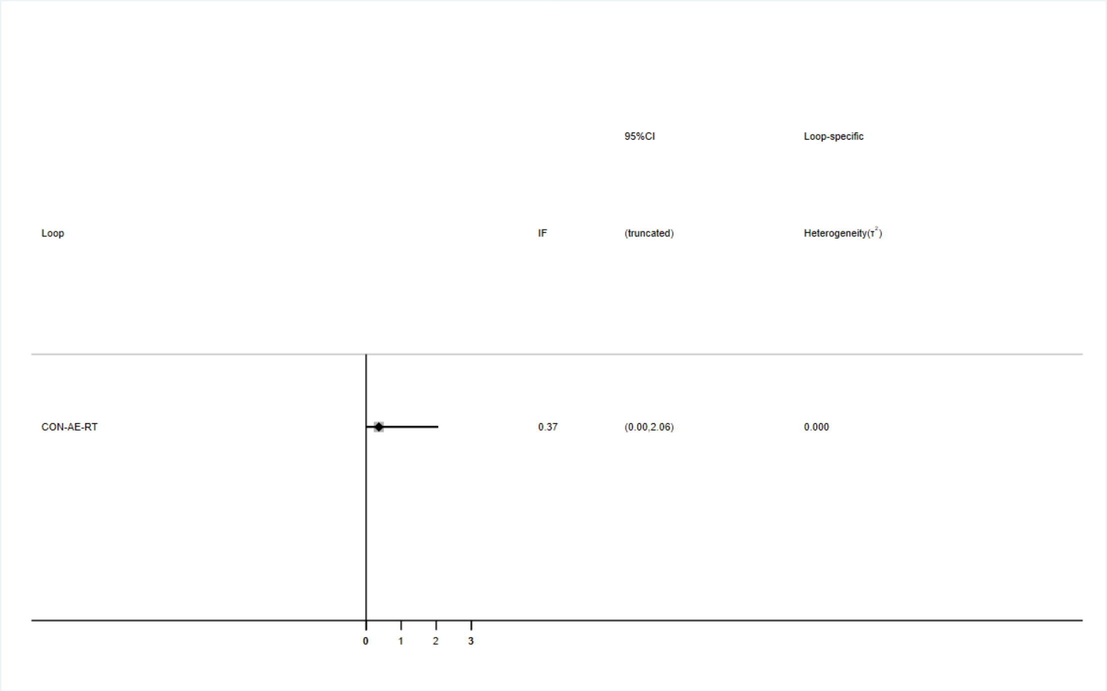

Appendix 16.4.1 Loop-specific heterogeneity for pulse wave velocity in short-term intervene..

Note. CON, non-exercise control; AE, aerobic exercise; INT, interval training.

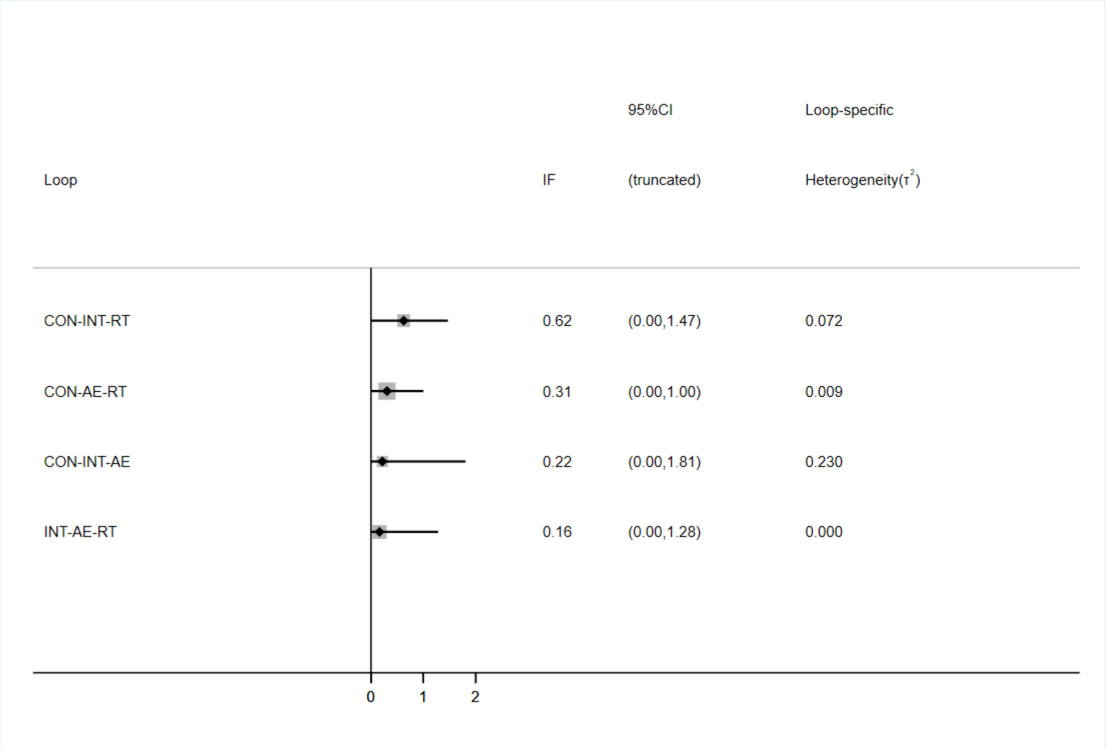

Appendix 15.4.2 Loop-specific heterogeneity for pulse wave velocity in medium-term intervene..

Note. CON means non-exercise control; AE, aerobic exercise RT means resistance training; INT means interval training.

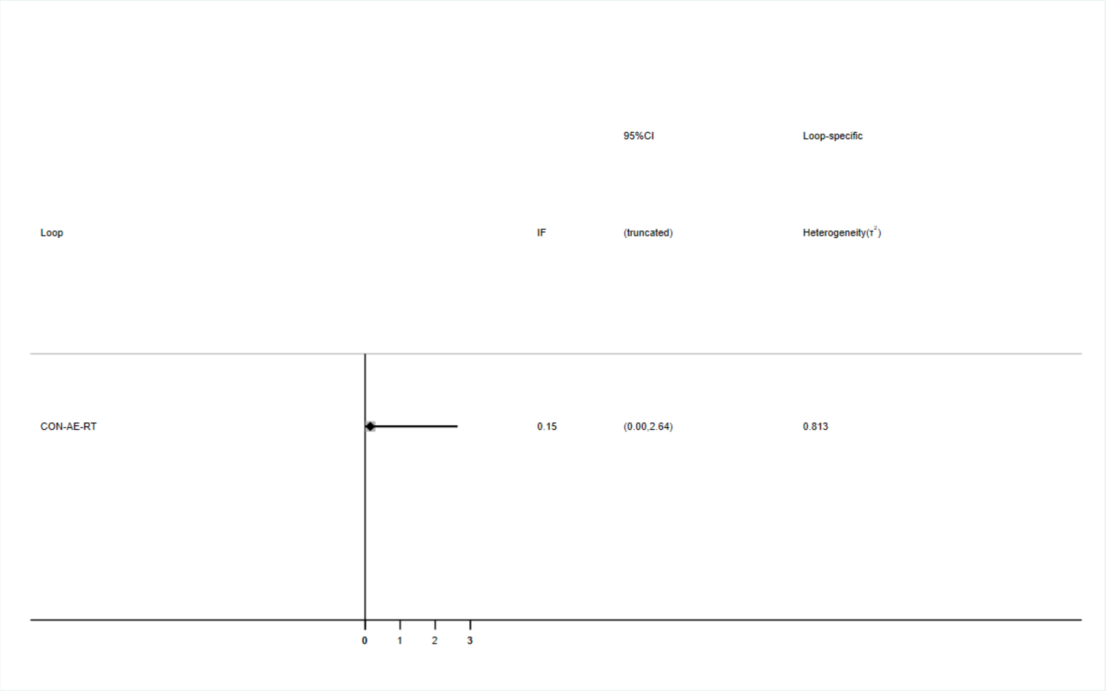

Appendix 15.4.3 Loop-specific heterogeneity for pulse wave velocity in long-term intervene.

Note. CON means non-exercise control; AE, aerobic exercise RT means resistance training.

Appendix 16.5 Local inconsistency test for Pulse Wave Velocity in different intervene duration.

Side	Direct Coef.	Std. Err.	Indirect Coef.	Std. Err.	Difference Coef.	Std.Err.	P>z
			Short-term intervene				
A C	-0.273	0.255	2.866	1.532	-3.140	1.550	0.043
A D	-0.710	0.519	-0.220	1.729	-0.490	1.816	0.787
C D	-0.922	0.796	0.008	0.698	-0.930	1.064	0.382
			Medium-term intervene				
A B	-0.921	0.191	-0.480	0.761	-0.441	0.785	0.574
A C	-0.571	0.315	-0.274	0.699	-0.297	0.780	0.703
A D	-0.370	0.145	-1.695	0.443	1.325	0.458	0.004
B C	0.114	0.618	0.461	0.348	-0.347	0.704	0.622
B D	0.054	0.327	0.605	0.321	-0.551	0.460	0.231
CD	-0.322	0.379	0.436	0.477	-0.758	0.612	0.215
			Long-term intervene				
A C	-0.666	0.286	-1.066	1.933	0.401	1.952	0.837
A D	-0.327	0.513	-0.080	1.549	-0.247	1.637	0.880
C D	0.502	0.808	0.271	0.707	0.231	1.082	0.831

Appendix 16.6 Prediction interval forest plots for Pulse Wave Velocity in different intervene duration.

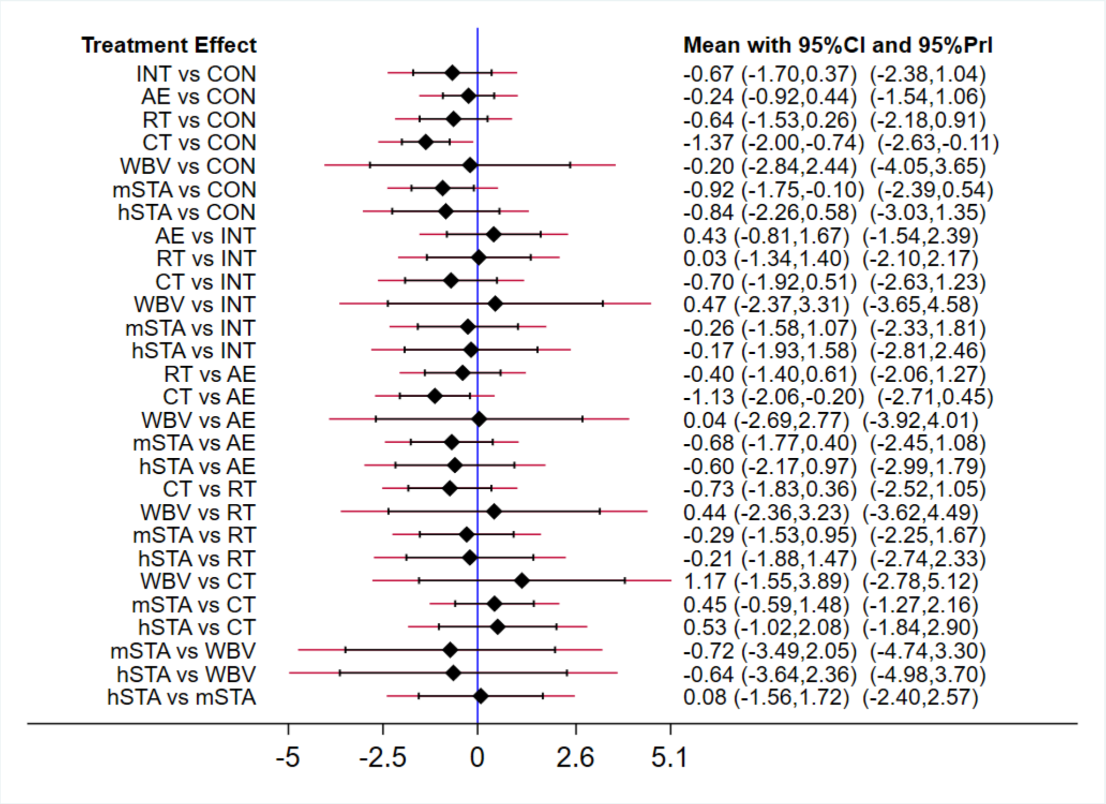

Appendix 16.6.1 Prediction interval forest plots for Pulse Wave Velocity in short-term intervene

Note. CON, control group; INT, interval training; AE, aerobic exercise; RT, resistance training; CT, combined training; WBV, whole-body vibration training; mSTA, moderate doses of statins; hSTA, high doses of statins.

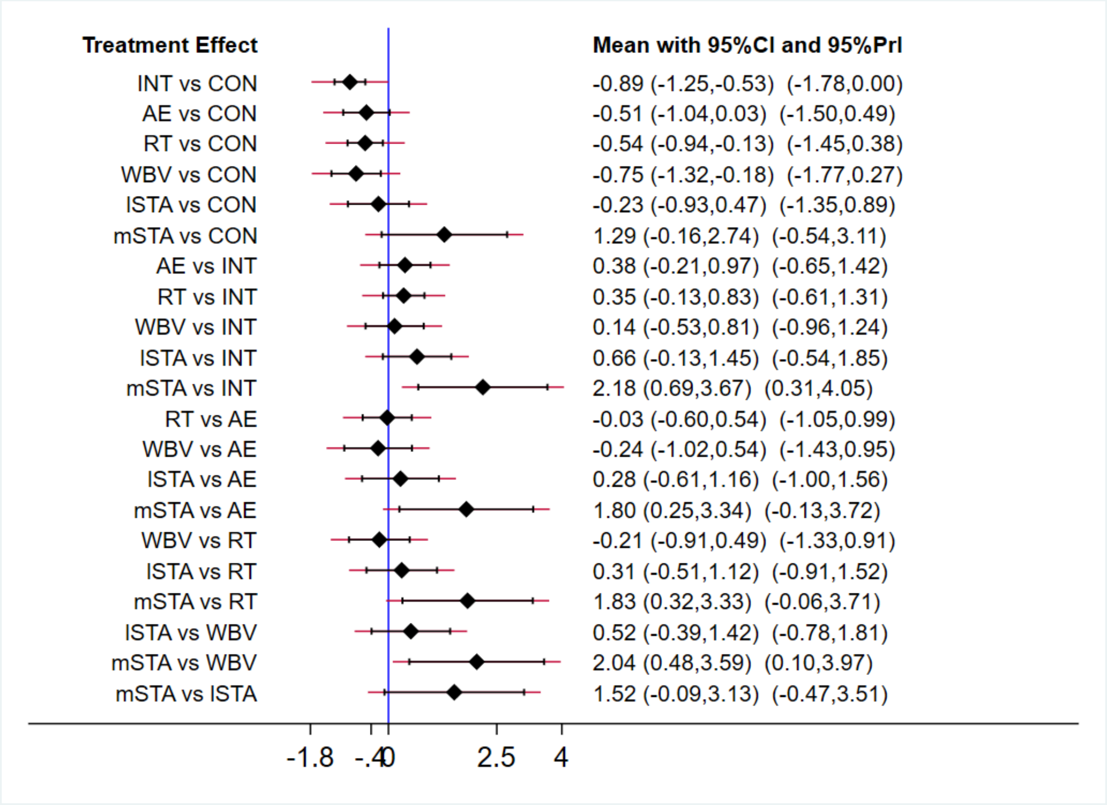

Appendix 16.6.2 Prediction interval forest plots for Pulse Wave Velocity in medium-term intervene

Note. CON, control group; INT, interval training; AE, aerobic exercise; RT, resistance training; CT, combined training; WBV, whole-body vibration training; lSTA, low doses of statins; mSTA, moderate doses of statins.

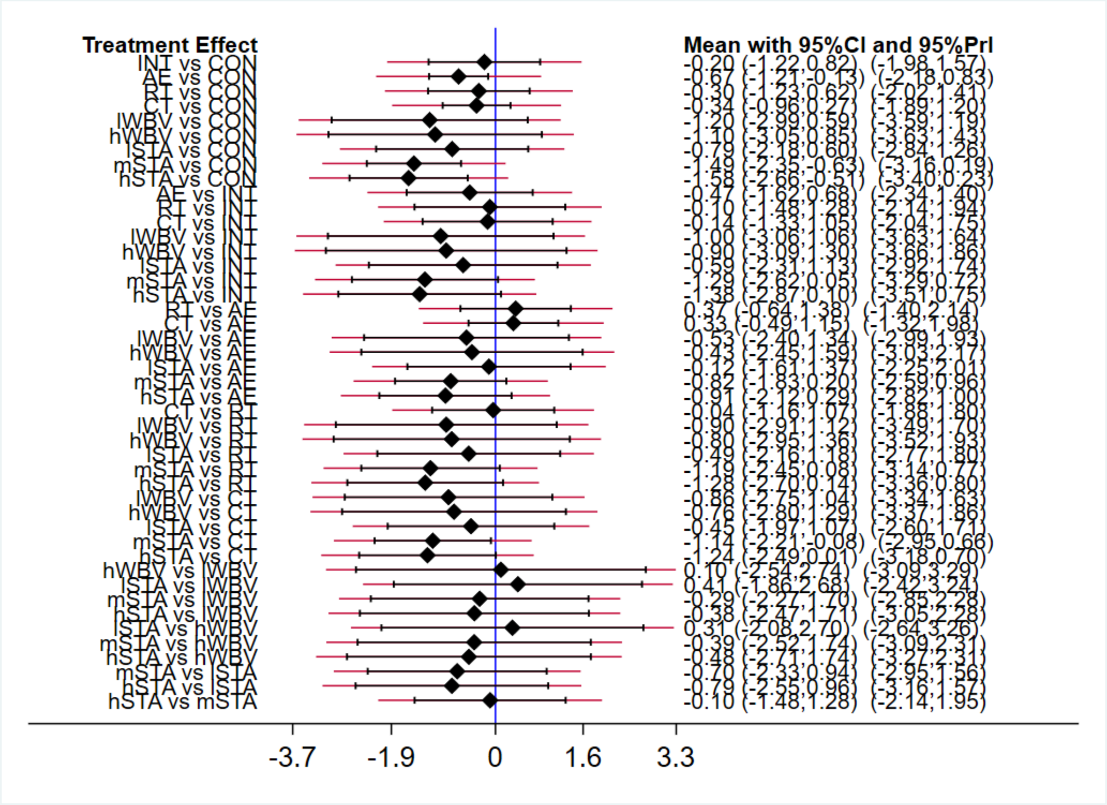

Appendix 16.6.2 Prediction interval forest plots for Pulse Wave Velocity in long-term intervene

Note. CON, control group; INT, interval training; AE, aerobic exercise; RT, resistance training; CT, combined training; lWBV, low intensity whole-body vibration training; hWBV means high intensity whole-body vibration training; lSTA, low doses of statins; mSTA, moderate doses of statins; hSTA means high doses of statins.

Appendix 16.7 Contributions of direct and indirect comparisons to NMA and the number of studies of each direct comparison of Pulse Wave Velocity in different intervene duration.

Note. A means control group; B means interval training; C means aerobic exercise; D means resistance training; E means combined training; F means whole-body vibration training; G means low doses of statins; H means moderate doses of statins; I means high doses of statins.

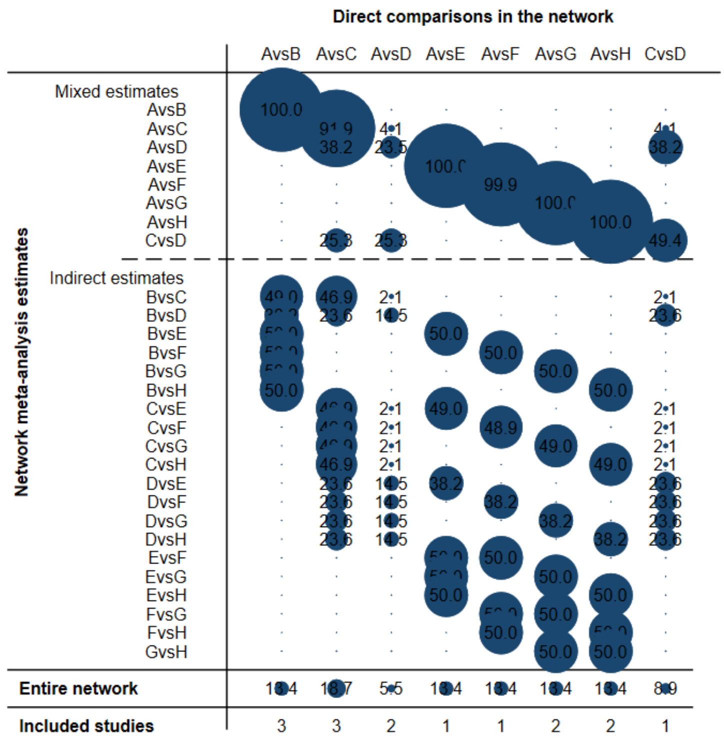

Appendix 16.7.1 Contribution plot for pulse wave velocity in short-term intervene.

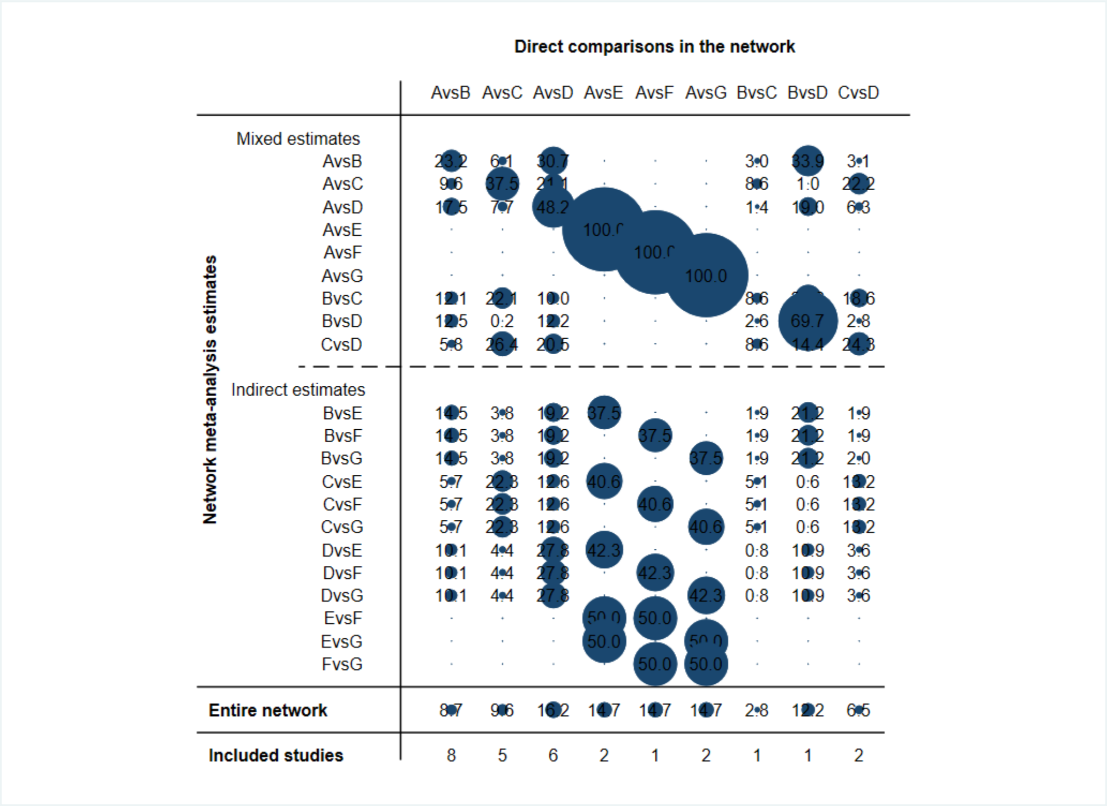

Appendix 16.7.2 Contribution plot for pulse wave velocity in medium-term intervene.

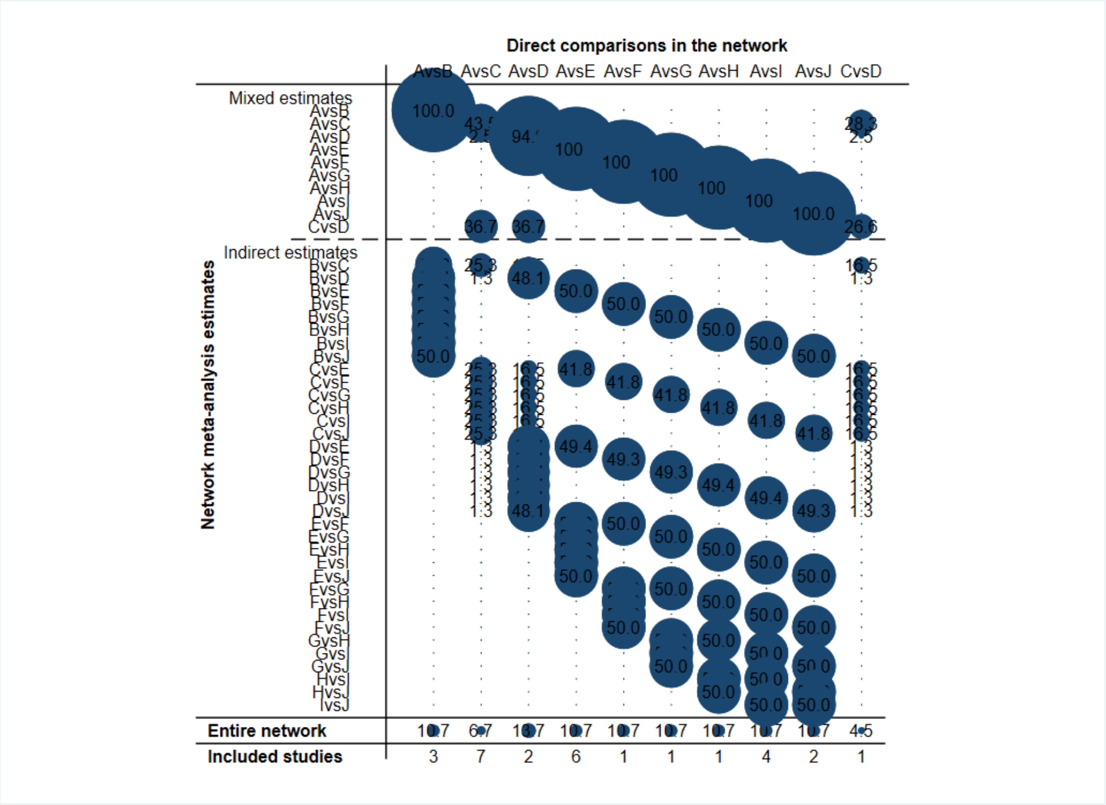

Appendix 16.7.3 Contribution plot for pulse wave velocity in long-term intervene.

Appendix 15.8 The funnel plot graphics of Pulse Wave Velocity in indifferent intervene duration.

Note. A means control group; B means interval training; C means aerobic exercise; D means resistance training; E means combined training; F means whole-body vibration training; G means low doses of statins; H means moderate doses of statins; I means high doses of statins.

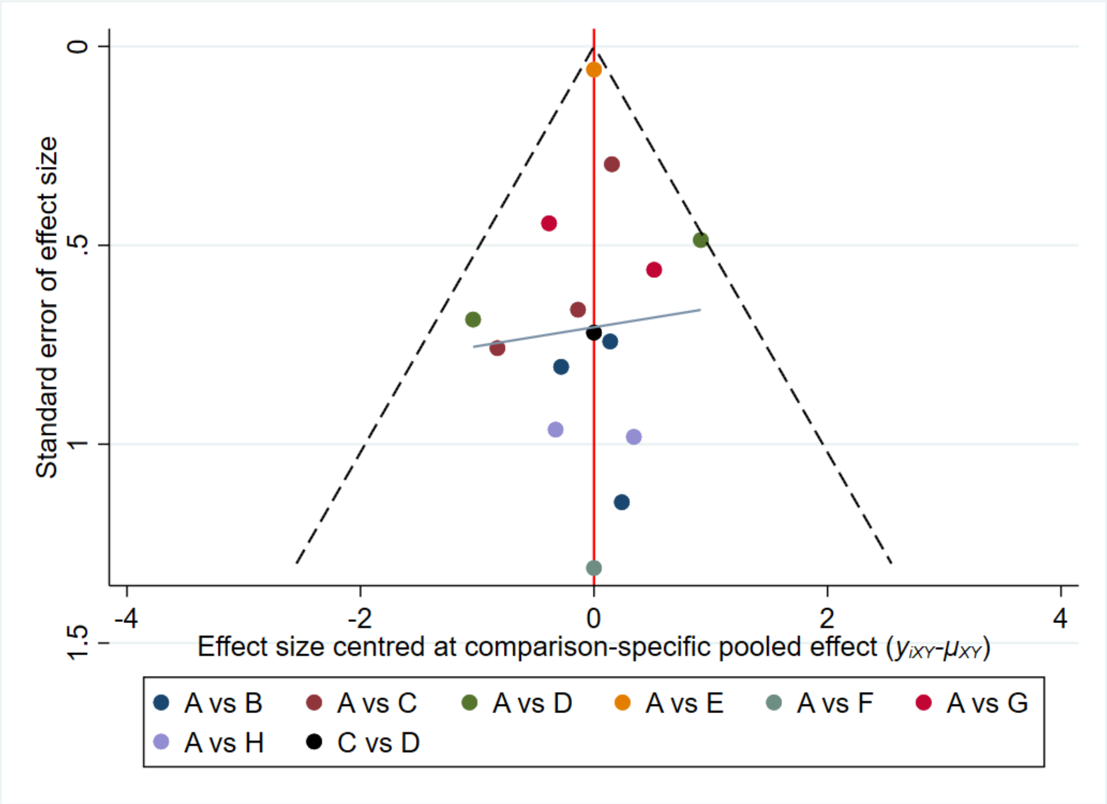

Appendix 16.8.1 Comparison-adjusted funnel plot for pulse wave velocity in short-term intervene

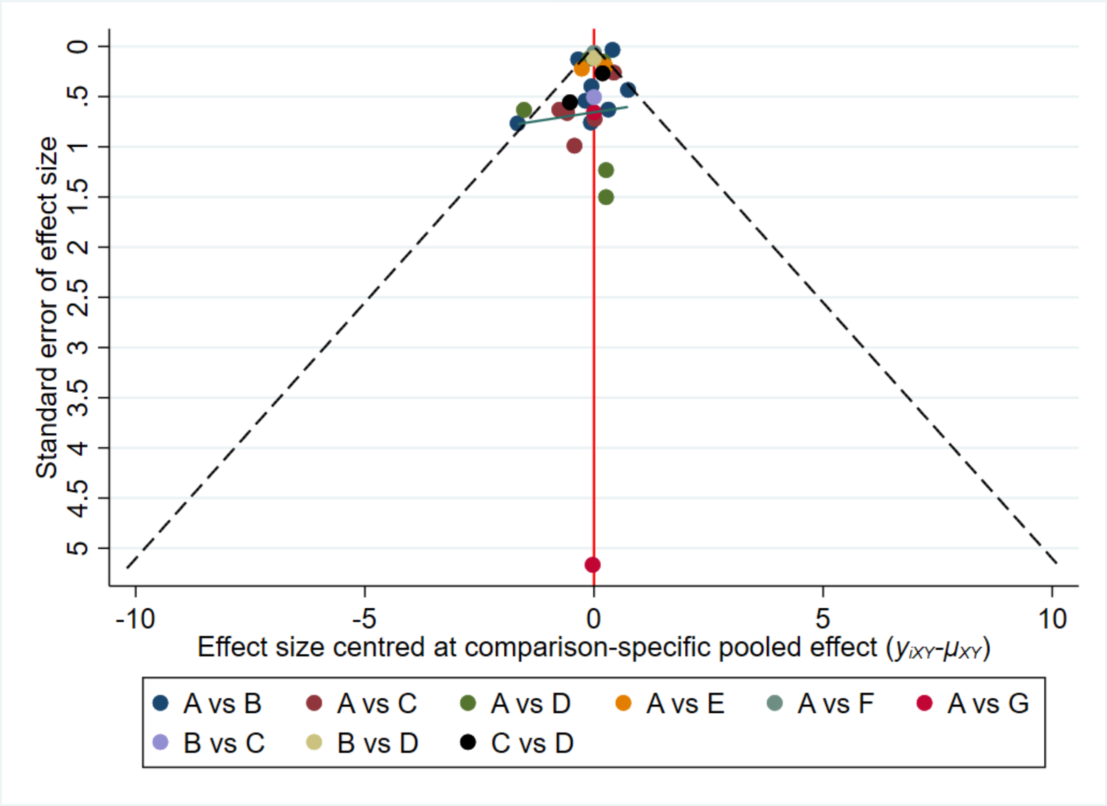

Appendix 16.8.2 Comparison-adjusted funnel plot for pulse wave velocity in medium-term intervene

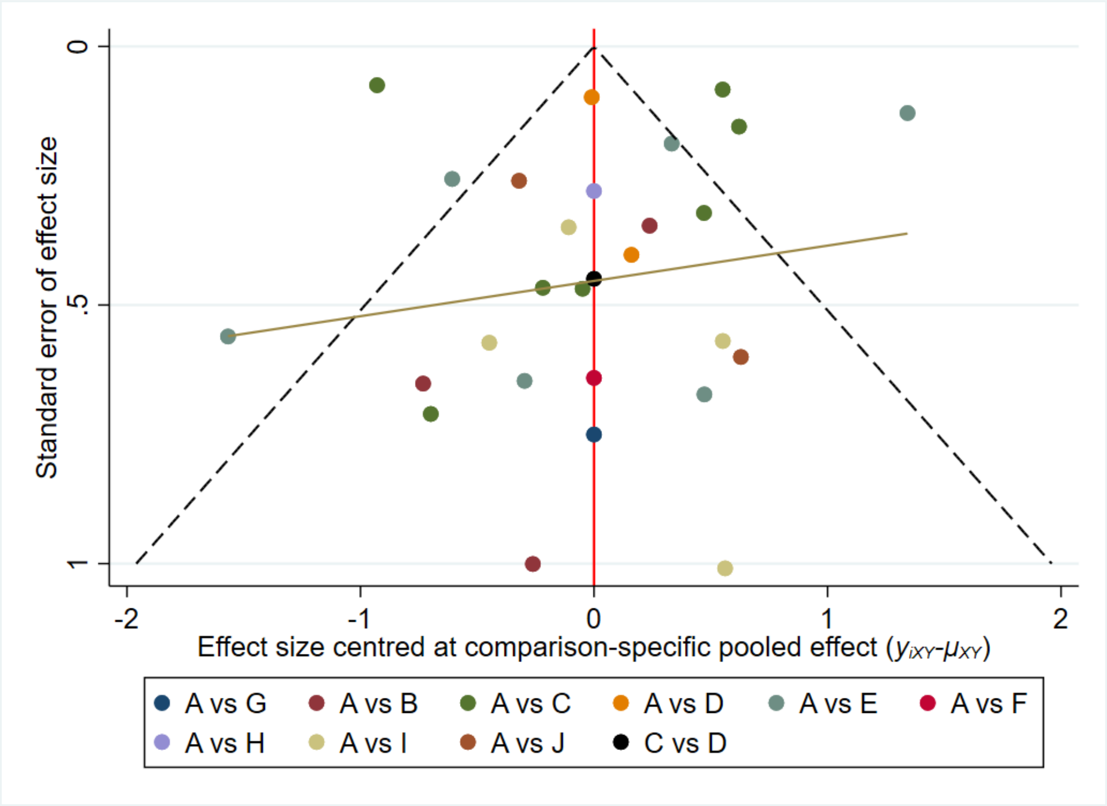

Appendix 16.8.3 Comparison-adjusted funnel plot for pulse wave velocity in long-term intervene

Appendix 15.9 Ranking of the effects of different types of exercise on pulse wave velocity in different intervene duration.

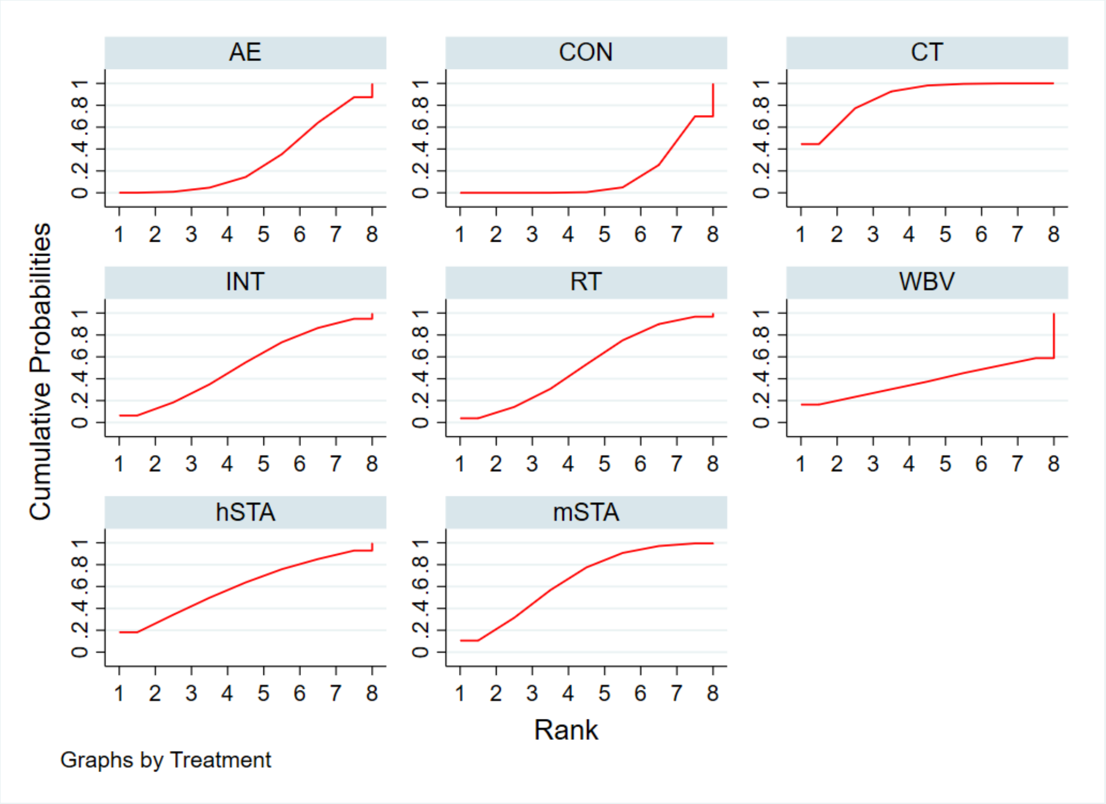

Appendix 16.9.1 Ranking of the effects of different types of exercise on pulse wave velocity in short-term intervene

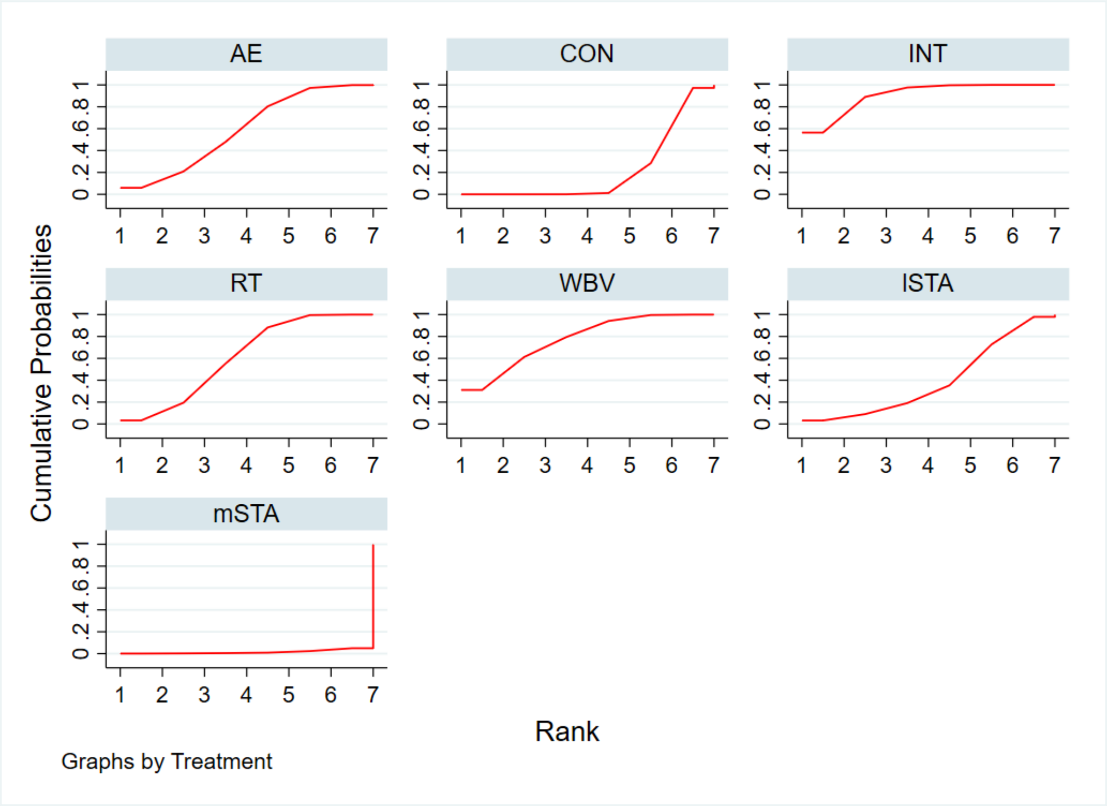

Appendix 16.9.2 Ranking of the effects of different types of exercise on pulse wave velocity in medium-term intervene

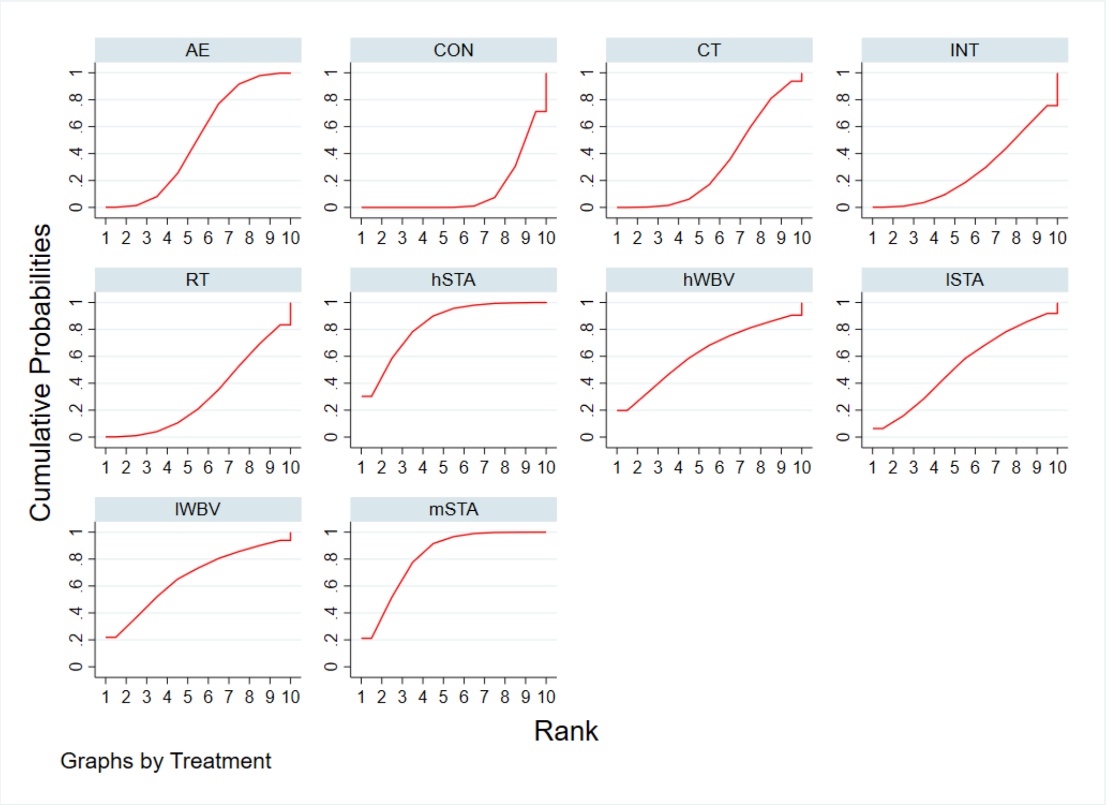

Appendix 16.9.3 Ranking of the effects of different types of exercise on pulse wave velocity in long-term intervene

Appendix 16.10 The ranking of effects of different exercise on pulse wave velocity indifferent intervene duration.

**Short-term intervene (13studies, N=485)**			
**Treatment**	SUCRA	PrBest (%)	Mean Rank
CT	87.4	44.5	1.9
mSTA	66.2	10.6	3.4
hSTA	60	18.2	3.8
INT	52.7	6.4	4.3
RT	52	3.8	4.4
WBV	37.7	16.4	5.4
AE	29.6	0.1	5.9
CON	14.4	0	7
**medium-term intervene (22studies, N=819)**			
**Treatment**	SUCRA	PrBest (%)	Mean Rank
INT	90.5	56.4	1.6
WBV	77.6	31.1	2.3
RT	61	3.3	3.3
AE	58.7	5.9	3.5
lSTA	39.6	3.2	4.6
CON	21.2	0	5.7
mSTA	1.5	0.1	6.9
**long-term intervene (27studies, N=1508)**			
**Treatment**	SUCRA	PrBest (%)	Mean Rank
hSTA	83.3	30.3	2.5
mSTA	81.9	21.2	2.6
lWBV	66.6	21.9	4
hWBV	62.1	19.8	4.4
lSTA	53	6.4	5.2
AE	50.3	0.1	5.5
CT	32.8	0	7.1
RT	30.8	0.2	7.2
INT	26.9	0.2	7.6
CON	12.3	0	8.9

Note. CON, control group; INT, interval training; AE, aerobic exercise; RT, resistance training; CT, combined training; lWBV, low intensity whole-body vibration training; hWBV means high intensity whole-body vibration training; lSTA, low doses of statins; mSTA, moderate doses of statins; hSTA means high doses of statins.

Appendix 15.11 Network meta-analysis matrix of PWV in different intensity.

**PWV in short-term intervene, m/s**							
**hSTA**	-0.08 (-1.72,1.56)	0.64 (-2.36,3.64)	-0.53 (-2.08,1.02)	0.21 (-1.47,1.88)	0.60 (-0.97,2.17)	0.17 (-1.58,1.93)	0.84 (-0.58,2.26)
0.08 (-1.56,1.72)	**mSTA**	0.72 (-2.05,3.49)	-0.45 (-1.48,0.59)	0.29 (-0.95,1.53)	0.68 (-0.40,1.77)	0.26 (-1.07,1.58)	**0.92 (0.10,1.75)**
-0.64 (-3.64,2.36)	-0.72 (-3.49,2.05)	**WBV**	-1.17 (-3.89,1.55)	-0.44 (-3.23,2.36)	-0.04 (-2.77,2.69)	-0.47 (-3.31,2.37)	0.20 (-2.44,2.84)
0.53 (-1.02,2.08)	0.45 (-0.59,1.48)	1.17 (-1.55,3.89)	**CT**	0.73 (-0.36,1.83)	**1.13 (0.20,2.06)**	0.70 (-0.51,1.92)	**1.37 (0.74,2.00)**
-0.21 (-1.88,1.47)	-0.29 (-1.53,0.95)	0.44 (-2.36,3.23)	-0.73 (-1.83,0.36)	**RT**	0.40 (-0.61,1.40)	-0.03 (-1.40,1.34)	0.64 (-0.26,1.53)
-0.60 (-2.17,0.97)	-0.68 (-1.77,0.40)	0.04 (-2.69,2.77)	**-1.13 (-2.06,-0.20)**	-0.40 (-1.40,0.61)	**AE**	-0.43 (-1.67,0.81)	0.24 (-0.44,0.92)
-0.17 (-1.93,1.58)	-0.26 (-1.58,1.07)	0.47 (-2.37,3.31)	-0.70 (-1.92,0.51)	0.03 (-1.34,1.40)	0.43 (-0.81,1.67)	**INT**	0.67 (-0.37,1.70)
-0.84 (-2.26,0.58)	**-0.92 (-1.75,-0.10)**	-0.20 (-2.84,2.44)	**-1.37 (-2.00,-0.74)**	-0.64 (-1.53,0.26)	-0.24 (-0.92,0.44)	-0.67 (-1.70,0.37)	**CON**

**PWV in medium-term intervene, m/s**						
**mSTA**	-1.52 (-3.13,0.09)	**-2.04 (-3.59,-0.48)**	**-1.83 (-3.33,-0.32)**	**-1.80 (-3.34,-0.25)**	**-2.18 (-3.67,-0.69)**	-1.29 (-2.74,0.16)
1.52 (-0.09,3.13)	**lSTA**	-0.52 (-1.42,0.39)	-0.31 (-1.12,0.51)	-0.28 (-1.16,0.61)	-0.66 (-1.45,0.13)	0.23 (-0.47,0.93)
**2.04 (0.48,3.59)**	0.52 (-0.39,1.42)	**WBV**	0.21 (-0.49,0.91)	0.24 (-0.54,1.02)	-0.14 (-0.81,0.53)	**0.75 (0.18,1.32)**
**1.83 (0.32,3.33)**	0.31 (-0.51,1.12)	-0.21 (-0.91,0.49)	**RT**	0.03 (-0.54,0.60)	-0.35 (-0.83,0.13)	**0.54 (0.13,0.94)**
**1.80 (0.25,3.34)**	0.28 (-0.61,1.16)	-0.24 (-1.02,0.54)	-0.03 (-0.60,0.54)	**AE**	-0.38 (-0.97,0.21)	0.51 (-0.03,1.04)
**2.18 (0.69,3.67)**	0.66 (-0.13,1.45)	0.14 (-0.53,0.81)	0.35 (-0.13,0.83)	0.38 (-0.21,0.97)	**INT**	**0.89 (0.53,1.25)**
1.29 (-0.16,2.74)	-0.23 (-0.93,0.47)	**-0.75 (-1.32,-0.18)**	**-0.54 (-0.94,-0.13)**	-0.51 (-1.04,0.03)	**-0.89 (-1.25,-0.53)**	**CON**

**PWV in long-term intervene, m/s**										
**hSTA**	0.10 (-1.28,1.48)	0.79 (-0.96,2.55)	0.48 (-1.74,2.71)	0.38 (-1.71,2.47)	1.24 (-0.01,2.49)	1.28 (-0.14,2.70)	0.91 (-0.29,2.12)	1.38 (-0.10,2.87)	**1.58 (0.51,2.66)**
-0.10 (-1.48,1.28)	**mSTA**	0.70 (-0.94,2.33)	0.39 (-1.74,2.52)	0.29 (-1.70,2.27)	**1.14 (0.08,2.21)**	1.19 (-0.08,2.45)	0.82 (-0.20,1.83)	1.29 (-0.05,2.62)	**1.49 (0.63,2.35)**
-0.79 (-2.55,0.96)	-0.70 (-2.33,0.94)	**lSTA**	-0.31 (-2.70,2.08)	-0.41 (-2.68,1.86)	0.45 (-1.07,1.97)	0.49 (-1.18,2.16)	0.12 (-1.37,1.61)	0.59 (-1.13,2.31)	0.79 (-0.60,2.18)
-0.48 (-2.71,1.74)	-0.39 (-2.52,1.74)	0.31 (-2.08,2.70)	**hWBV**	-0.10 (-2.74,2.54)	0.76 (-1.29,2.80)	0.80 (-1.36,2.95)	0.43 (-1.59,2.45)	0.90 (-1.30,3.09)	1.10 (-0.85,3.05)
-0.38 (-2.47,1.71)	-0.29 (-2.27,1.70)	0.41 (-1.86,2.68)	0.10 (-2.54,2.74)	**lWBV**	0.86 (-1.04,2.75)	0.90 (-1.12,2.91)	0.53 (-1.34,2.40)	1.00 (-1.06,3.06)	1.20 (-0.59,2.99)
-1.24 (-2.49,0.01)	**-1.14 (-2.21,-0.08)**	-0.45 (-1.97,1.07)	-0.76 (-2.80,1.29)	-0.86 (-2.75,1.04)	**CT**	0.04 (-1.07,1.16)	-0.33 (-1.15,0.49)	0.14 (-1.05,1.33)	0.34 (-0.27,0.96)
-1.28 (-2.70,0.14)	-1.19 (-2.45,0.08)	-0.49 (-2.16,1.18)	-0.80 (-2.95,1.36)	-0.90 (-2.91,1.12)	-0.04 (-1.16,1.07)	**RT**	-0.37 (-1.38,0.64)	0.10 (-1.28,1.48)	0.30 (-0.62,1.23)
-0.91 (-2.12,0.29)	-0.82 (-1.83,0.20)	-0.12 (-1.61,1.37)	-0.43 (-2.45,1.59)	-0.53 (-2.40,1.34)	0.33 (-0.49,1.15)	0.37 (-0.64,1.38)	**AE**	0.47 (-0.68,1.62)	**0.67 (0.13,1.21)**
-1.38 (-2.87,0.10)	-1.29 (-2.62,0.05)	-0.59 (-2.31,1.13)	-0.90 (-3.09,1.30)	-1.00 (-3.06,1.06)	-0.14 (-1.33,1.05)	-0.10 (-1.48,1.28)	-0.47 (-1.62,0.68)	**INT**	0.20 (-0.82,1.22)
**-1.58 (-2.66,-0.51)**	**-1.49 (-2.35,-0.63)**	-0.79 (-2.18,0.60)	-1.10 (-3.05,0.85)	-1.20 (-2.99,0.59)	-0.34 (-0.96,0.27)	-0.30 (-1.23,0.62)	**-0.67 (-1.21,-0.13)**	-0.20 (-1.22,0.82)	**CON**

Efficacy (response rate) Comparison Acceptability (dropout rate) Group

Note: Effects are expressed as the effect size [95% CI] between interventions. Bold indicates that the data are significant, light cyan areas indicate the effect of the longitudinal versus the lateral intervention, pink areas indicate the effect of the lateral versus the longitudinal intervention, grey areas represent the intervention category, and black areas represent the group. For example, "-1.37 (-2.00,-0.74)" (column 7, row 4), which indicates that INT (longitudinal intervention) significantly reduces PWV compared with CON (transverse intervention).

Abbreviations: INT, means interval training; AE means aerobic exercise; RT means resistance exercise; CT means combined training; CON means control group; PWV means Pulse Wave Velocity; SBP means Systolic Blood Pressure; DBP means Diastolic Blood Pressure

Appendix 16. List of included studies.

1. Guimarães GV, Ciolac EG, Carvalho VO, D'Avila VM, Bortolotto LA, Bocchi EA. Effects of continuous vs. interval exercise training on blood pressure and arterial stiffness in treated hypertension. Hypertens Res. 2010; 33(6): 627-632. https://doi.org/10.1038/hr.2010.42.
2. Bouaziz W, Lang P-O, Schmitt E, Leprêtre P-M, Lefebvre F, Momas C, Effects of a short-term interval aerobic training program with recovery bouts on vascular function in sedentary aged 70 or over: A randomized controlled trial. Arch Gerontol Geriatr. 2019; 82: 217-225. https://doi.org/10.1016/j.archger.2019.02.017.
3. Aghaei Bahmanbeglou N, Ebrahim K, Maleki M, Nikpajouh A, Ahmadizad S. Short-Duration High-Intensity Interval Exercise Training Is More Effective Than Long Duration for Blood Pressure and Arterial Stiffness But Not for Inflammatory Markers and Lipid Profiles in Patients With Stage 1 Hypertension. J Cardiopulm Rehabil Prev. 2019; 39(1). https://doi.org/10.1097/HCR.0000000000000377.
4. Zhuang X , Xu H, Zheng J, Effect of high intensity interval training on vascular wall elasticity in the elderly. 2023; 45(05): 429-431. https:// doi.org/10.3760/cma.j.issn.0254-1424.2023.05.009.
5. Mora-Rodriguez R, Ramirez-Jimenez M, Fernandez-Elias VE, Guio de Prada MV, Morales-Palomo F, Pallares JG, Effects of aerobic interval training on arterial stiffness and microvascular function in patients with metabolic syndrome. J Clin Hypertens. 2018; 20(1): 11-18. https://doi.org/10.1111/jch.13130.
6. Deiseroth A, Streese L, Köchli S, Wüst RS, Infanger D, Schmidt-Trucksäss A, Exercise and arterial stiffness in the elderly: a combined cross-sectional and randomized controlled trial (EXAMIN AGE). Front Physiol. 2019; 10: 474298. https://doi.org/10.3389/fphys.2019.01119.
7. Zhang HM, Xie XF, Xiao Y, Effect of high-intensity interval training and

medium-intensity continuous training on vascular health in patients with type 2 diabetes mellitus. Journal of medical Graduate students. 2022; 35(01): 63-68. https:// doi.org/ 10.16571/j.cnki.1008-8199.2022.01.012.

8. Kim HK, Hwang CL, Yoo JK, Hwang MH, Handberg EM, Petersen JW, All-Extremity Exercise Training Improves Arterial Stiffness in Older Adults. Med Sci Sports Exerc. 2017; 49(7): 1404-1411. https://doi.org/10.1249/mss.0000000000001229.

9. Bellia A, Iellamo F, De Carli E, Andreadi A, Padua E, Lombardo M, Exercise individualized by TRIMPi method reduces arterial stiffness in early onset type 2 diabetic patients: A randomized controlled trial with aerobic interval training. Int J Cardiol. 2017; 248: 314-319. https://doi.org/10.1016/j.ijcard.2017.06.065.

10. Way KL, Sabag A, Sultana RN, Baker MK, Keating SE, Lanting S, The effect of low-volume high-intensity interval training on cardiovascular health outcomes in type 2 diabetes: A randomised controlled trial. Int J Cardiol. 2020; 320: 148-154. https://doi.org/10.1016/j.ijcard.2020.06.019.

11. Adams SC, DeLorey DS, Davenport MH, Stickland MK, Fairey AS, North S, Effects of high-intensity aerobic interval training on cardiovascular disease risk in testicular cancer survivors: A phase 2 randomized controlled trial. Cancer. 2017; 123(20): 4057-4065. https://doi.org/10.1002/cncr.30859.

12. Taha MM, Aneis YM, Hasanin ME, Felaya EE, Aldhahi MI, Abdeen HAA. Effect of high intensity interval training on arterial stiffness in obese hypertensive women: a randomized controlled trial. Eur Rev Med Pharmacol Sci. 2023; 27(9): 4069-4079. https://doi.org/10.26355/eurrev_202305_32314.

13. Bouaziz W, Lang PO, Schmitt E, Leprêtre PM, Lefebvre F, Momas C, Effects of a short-term interval aerobic training program with recovery bouts on vascular function in sedentary aged 70 or over: A randomized controlled trial. Arch Gerontol Geriatr. 2019; 82: 217-225. https://doi.org/10.1016/j.archger.2019.02.017.

14. Millen AME, Norton GR, Avidon I, Woodiwiss AJ. Effects of short-term exercise-training on aortic systolic pressure augmentation in overweight and obese individuals. Eur J Appl Physiol. 2013; 113(7): 1793-1803. https://doi.org/10.1007/s00421-013-2610-2.
15. Kearney TM, Murphy MH, Davison GW, O'Kane MJ, Gallagher AM. Accumulated brisk walking reduces arterial stiffness in overweight adults: Evidence from a randomized control trial. J Am Soc Hypertens. 2014; 8(2): 117-126. https://doi.org/10.1016/j.jash.2013.10.001.
16. Nualnim N, Parkhurst K, Dhindsa M, Tarumi T, Vavrek J, Tanaka H. Effects of Swimming Training on Blood Pressure and Vascular Function in Adults >50 Years of Age. Am J Cardiol. 2012; 109(7): 1005-1010. https://doi.org/10.1016/j.amjcard.2011.11.029.
17. Zhou LY, Wu ZZ, Hong LF. Effect of 12-week aerobic exercise training on brachial-ankle, pulse wave velocity in elderly. 2010; 32(05): 356-358. https:// doi.org/ :10.3760/ema. j. issn. 0254-1424.2010.05.009
18. Madden KM, Lockhart C, Cuff D, Potter TF, Meneilly GS. Aerobic training-induced improvements in arterial stiffness are not sustained in older adults with multiple cardiovascular risk factors. J Hum Hypertens. 2013; 27(5): 335-339. https://doi.org/10.1038/jhh.2012.38.
19. Horner K, Barinas-Mitchell E, DeGroff C, Kuk JL, Drant S, Lee S. Effect of aerobic versus resistance exercise on pulse wave velocity, intima media thickness and left ventricular mass in obese adolescents. Pediatr Exerc Sci. 2015; 27(4): 494-502. https://doi.org/10.1123/pes.2015-0067.
20. Huang CY, Pan ML, Zhang L,　Effect of maximum fat oxidation intensity exercise on arterial stiffness in overweight/obese young men. 2018; 37(01): 3-9. https:// doi.org/ 10.16038/j.1000-6710.2018.01.001.
21. Shin J-H, Lee Y, Kim SG, Choi BY, Lee H-S, Bang S-Y. The beneficial effects of Tai Chi exercise on endothelial function and arterial stiffness in elderly women

with rheumatoid arthritis. Arthritis Res Ther. 2015; 17(1): 380. https://doi.org/10.1186/s13075-015-0893-x.

22. Davis CL, Litwin SE, Pollock NK, Waller JL, Zhu H, Dong Y, Exercise effects on arterial stiffness and heart health in children with excess weight: The SMART RCT. Int J Obes. 2020; 44(5): 1152-1163. https://doi.org/10.1038/s41366-019-0482-1.
23. Chen TT, Li lC, Yin XL, Study on the influence of Taijiquan on PWV, ABL and ADP in middle-aged and elderly people. 2018; 35(18): 134-135+150. https:// doi.org/10.3969/j.issn.1674-151x.2018.18.067
24. Madden KM, Lockhart C, Cuff D, Potter TF, Meneilly GS. Short-term aerobic exercise reduces arterial stiffness in older adults with type 2 diabetes, hypertension, and hypercholesterolemia. Diabetes Care. 2009; 32(8): 1531-1535. https://doi.org/10.2337/dc09-0149.
25. O'Connor EM, Koufaki P, Mercer TH, Lindup H, Nugent E, Goldsmith D, Long-term pulse wave velocity outcomes with aerobic and resistance training in kidney transplant recipients - A pilot randomised controlled trial. PLoS One. 2017; 12(2): e0171063. https://doi.org/10.1371/journal.pone.0171063.
26. Jaime SJ. The Effects of Twelve Weeks of Whole-Body Vibration Training and Low-Intensity Resistance Exercise Training on Arterial Function, Muscle Strength, and Physical Performance in Dynapenic Postmenopausal Women. 2017.
27. Croymans DM, Krell SL, Oh CS, Katiraie M, Lam CY, Harris RA, Effects of resistance training on central blood pressure in obese young men. Randomized Controlled Trial. 2014; 28(3): 157-164. https://doi.org/10.1038/jhh.2013.81.
28. DeVallance E, Fournier S, Lemaster K, Moore C, Asano S, Bonner D, The effects of resistance exercise training on arterial stiffness in metabolic syndrome. Eur J Appl Physiol. 2016; 116(5): 899-910. https://doi.org/10.1007/s00421-016-3348-4.
29. Greenwood SA, Koufaki P, Mercer TH, Rush R, O'Connor E, Tuffnell R,

Aerobic or Resistance Training and Pulse Wave Velocity in Kidney Transplant Recipients: A 12-Week Pilot Randomized Controlled Trial (the Exercise in Renal Transplant [ExeRT] Trial). Am J Kidney Dis. 2015; 66(4): 689-698. https://doi.org/10.1053/j.ajkd.2015.06.016.

30. Beck DT, Martin JS, Casey DP, Braith RW. Exercise Training Reduces Peripheral Arterial Stiffness and Myocardial Oxygen Demand in Young Prehypertensive Subjects. American Journal of Hypertension. 2013; 26(9): 1093-1102. https://doi.org/10.1093/ajh/hpt080.
31. Fernandez-del-Valle M, Gonzales JU, Kloiber S, Mitra S, Klingensmith J, Larumbe-Zabala E. Effects of resistance training on MRI-derived epicardial fat volume and arterial stiffness in women with obesity: a randomized pilot study. Eur J Appl Physiol. 2018; 118(6): 1231-1240. https://doi.org/10.1007/s00421-018-3852-9.
32. Jaime SJ, Maharaj A, Alvarez-Alvarado S, Figueroa A. Impact of low-intensity resistance and whole-body vibration training on aortic hemodynamics and vascular function in postmenopausal women. Hypertens Res. 2019; 42(12): 1979-1988. https://doi.org/10.1038/s41440-019-0328-1.
33. Ramírez-Vélez R, Castro-Astudillo K, Correa-Bautista JE, González-Ruíz K, Izquierdo M, García-Hermoso A, The Effect of 12 Weeks of Different Exercise Training Modalities or Nutritional Guidance on Cardiometabolic Risk Factors, Vascular Parameters, and Physical Fitness in Overweight Adults: Cardiometabolic High-Intensity Interval Training-Resistance Training Randomized Controlled Study. J Strength Cond Res. 2020; 34(8). https://doi.org/10.1519/JSC.0000000000003533.
34. Wong A, Sanchez-Gonzalez MA, Son W-M, Kwak Y-S, Park S-Y. The effects of a 12-week combined exercise training program on arterial stiffness, vasoactive substances, inflammatory markers, metabolic profile, and body composition in obese adolescent girls. Pediatr Exerc Sci. 2018; 30(4): 480-486.

https://doi.org/10.1123/pes.2017-0198.

35. Lee YH, Park SH, Yoon ES, Lee C-D, Wee SO, Fernhall B, Effects of Combined Aerobic and Resistance Exercise on Central Arterial Stiffness and Gait Velocity in Patients with Chronic Poststroke Hemiparesis. Am J Phys Med Rehabil. 2015; 94(9). https://doi.org/10.1097/PHM.0000000000000233.
36. Shiotsu Y, Watanabe Y, Tujii S, Yanagita M. Effect of exercise order of combined aerobic and resistance training on arterial stiffness in older men. Exp Gerontol. 2018; 111: 27-34. https://doi.org/10.1016/j.exger.2018.06.020.
37. Stewart KJ, Bacher AC, Turner KL, Fleg JL, Hees PS, Shapiro EP, Effect of Exercise on Blood Pressure in Older Persons: A Randomized Controlled Trial. Arch Intern Med. 2005; 165(7): 756-762. https://doi.org/10.1001/archinte.165.7.756.
38. Park W, Jung W-S, Hong K, Kim Y-Y, Kim S-W, Park H-Y. Effects of Moderate Combined Resistance- and Aerobic-Exercise for 12 Weeks on Body Composition, Cardiometabolic Risk Factors, Blood Pressure, Arterial Stiffness, and Physical Functions, among Obese Older Men: A Pilot Study. Int J Environ Res Public Health. 2020; 17(19): 7233. https://doi.org/10.3390/ijerph17197233.
39. Son W-M, Sung K-D, Cho J-M, Park S-Y. Combined exercise reduces arterial stiffness, blood pressure, and blood markers for cardiovascular risk in postmenopausal women with hypertension. Menopause. 2017; 24(3): 262-268. https://doi.org/10.1097/gme.0000000000000765.
40. Dobrosielski DA, Gibbs BB, Ouyang P, Bonekamp S, Clark JM, Wang N-Y, Effect of Exercise on Blood Pressure in Type 2 Diabetes: A Randomized Controlled Trial. J Gen Intern Med. 2012; 27(11): 1453-1459. https://doi.org/10.1007/s11606-012-2103-8.
41. Loimaala A, Groundstroem K, Rinne M, Nenonen A, Huhtala H, Parkkari J, Effect of Long-Term Endurance and Strength Training on Metabolic Control and Arterial Elasticity in Patients With Type 2 Diabetes Mellitus. Am J Cardiol. 2009;

103(7): 972-977. https://doi.org/10.1016/j.amjcard.2008.12.026.

42. Figueroa A, Kalfon R, Madzima TA, Wong A. Whole-body vibration exercise training reduces arterial stiffness in postmenopausal women with prehypertension and hypertension. Menopause. 2014; 21(2). https://doi.org/10.1097/gme.0b013e318294528c.
43. Figueroa A, Gil R, Wong A, Hooshmand S, Park SY, Vicil F, et al. Whole-body vibration training reduces arterial stiffness, blood pressure and sympathovagal balance in young overweight/obese women. Hypertension Research. 2012; 35(6): 667-672. https://doi.org/10.1038/hr.2012.15.
44. Figueroa A, Kalfon R, Wong A. Whole-body vibration training decreases ankle systolic blood pressure and leg arterial stiffness in obese postmenopausal women with high blood pressure. Menopause. 2015; 22(4). https://doi.org/10.1097/GME.0000000000000332.
45. Alvarez-Alvarado S, Jaime SJ, Ormsbee MJ, Campbell JC, Post J, Pacilio J, et al. Benefits of whole-body vibration training on arterial function and muscle strength in young overweight/obese women. Hypertension Research. 2017; 40(5): 487-492. https://doi.org/10.1038/hr.2016.178.
46. Yule CE, Stoner L, Hodges LD, Cochrane DJ. Does short-term whole-body vibration training affect arterial stiffness in chronic stroke? A preliminary study. Journal of Physical Therapy Science. 2016; 28(3): 996-1002. https://doi.org/10.1589/jpts.28.996.
47. Raison J, Rudnichi A, Safar ME. Effects of atorvastatin on aortic pulse wave velocity in patients with hypertension and hypercholesterolaemia: a preliminary study. Journal of Human Hypertension. 2002; 16(10): 705-710. https://doi.org/10.1038/sj.jhh.1001470.
48. Kanaki AI, Sarafidis PA, Georgianos PI, Kanavos K, Tziolas IM, Zebekakis PE, et al. Effects of Low-Dose Atorvastatin on Arterial Stiffness and Central Aortic Pressure Augmentation in Patients With Hypertension and Hypercholesterolemia.

American Journal of Hypertension. 2013; 26(5): 608-616. https://doi.org/10.1093/ajh/hps098.

49. Orr JS, Dengo AL, Rivero JM, Davy KP. Arterial Destiffening With Atorvastatin in Overweight and Obese Middle-Aged and Older Adults. Hypertension. 2009; 54(4): 763-768. https://doi.org/10.1161/HYPERTENSIONAHA.109.138248.
50. Pirro M, Schillaci G, Mannarino MR, Savarese G, Vaudo G, Siepi D, et al. Effects of rosuvastatin on 3-nitrotyrosine and aortic stiffness in hypercholesterolemia. Nutrition, Metabolism and Cardiovascular Diseases. 2007; 17(6): 436-441. https://doi.org/10.1016/j.numecd.2006.02.009.
51. Igase M, Kohara K, Tabara Y, Nagai T, Ochi N, Kido T, et al. Low-dose rosuvastatin improves the functional and morphological markers of atherosclerosis in asymptomatic postmenopausal women with dyslipidemia. Menopause. 2012; 19(12): 1294-1299. https://doi.org/10.1097/gme.0b013e318259c04e.
52. Grigoropoulou P, Tentolouris A, Eleftheriadou I, Tsilingiris D, Vlachopoulos C, Sykara M, et al. Effect of 12-month intervention with low-dose atorvastatin on pulse wave velocity in subjects with type 2 diabetes and dyslipidaemia. Diab Vasc Dis Res. 2019; 16(1): 38-46. https://doi.org/10.1177/1479164118805320.
53. Qi Deqin, Chen Hong, Yurong L. Effect of atorvastatin on arterial elasticity and ankle-brachial index in elderly patients with hypertension. Journal of Chinese senile cardio-cerebrovascular diseases. 2008; 07: 510-512.
54. Chen Hezhong, Xiuhui Y. Effect of atorvastatin on pulse wave conduction velocity in patients with essential hypertension. Journal of Chinese Hpertension. 2009; 17(06): 553-554. https://doi.org/10.16439/j.cnki.1673-7245.2009.06.012.
55. Zhang Weizhong, Ding Yueyou, Huili Q. Journal of Hpertension. Clinical Study of Fluvastatin on Improvement of Pulse Pressure and Arterial Elasticity in Patients of Hypertension. 2003; (06): 12-15. https://doi.org/10.16439/j.cnki.1673-7245.2003.06.003.

56. Oh PC, Han SH, Koh KK, Lee K, Seo JG, Suh SY, et al. Rosuvastatin treatment improves arterial stiffness with lowering blood pressure in healthy hypercholesterolemic patients. International Journal of Cardiology. 2014; 176(3): 1284-1287. https://doi.org/10.1016/j.ijcard.2014.07.181.
57. Mukherjee S, Mukhopadhyay P, Pandit K, Mukherjee S, Chowdhury S. Atorvastatin improves arterial stiffness in normotensive normolipidaemic persons with type 2 diabetes. J Indian Med Assoc. 2008; 106(11): 716-719.
58. Ichihara A, Hayashi M, Koura Y, Tada Y, Kaneshiro Y, Saruta T. Long-term effects of statins on arterial pressure and stiffness of hypertensives. J Hum Hypertens. 2005; 19(2): 103-109. https://doi.org/10.1038/sj.jhh.1001786.
